# A cyclin-polarity feedback network ensures healthy cell proliferation

**DOI:** 10.1038/s41467-026-74596-7

**Published:** 2026-06-19

**Authors:** Landry Peyran, Charles Lefranc, Steven P. Gygi, Anne Royou, Derek McCusker

**Affiliations:** 1 Univ. Bordeaux, CNRS, Institut de Biochimie et Génétique Cellulaires, UMR 5095; 33077, Bordeaux, France; 2 Department of Cell Biology, Harvard Medical School, Boston, MA USA

**Keywords:** Cell division, Kinases, Cell growth, Cell signalling

## Abstract

Healthy proliferation requires the coordination of cell cycle progression with cell polarity. In budding yeast, polarity is established when G1-cyclin-Cdc28^Cdk1^ triggers Cdc42 activation to generate a cell pole that is used as an axis for growth and division. While polarity defects delay the cell cycle temporally, permitting error correction, it is unknown if Cdc28^Cdk1^ directly rectifies errant polarity. Here, we identify an adaptive response where G1-cyclin-Cdc28^Cdk1^ participates in error correction via the augmentation of its kinase activity towards substrates that activate Cdc42. The response involves temporal and spatial cell cycle reconfiguration via extended G1 cyclin expression, nucleocytoplasmic rerouting and signaling. However, this strategy has a cost: if the defect is irreparable, high G1-cyclin levels enforce inexorable cell cycle commitment in the absence of a daughter cell, generating multinucleate cells. G1-cyclins therefore not only trigger G1 events, but also monitor their execution, employing feedback to coordinate polarity with cell cycle progression.

## Abstract (web summary)

The mechanisms that coordinate cell polarity and the cell cycle are incompletely understood. Here, the authors show that defects in polarity trigger an adaptive response that reconfigures cyclin-dependent kinase activity both temporally and spatially to correct polarity defects.

## Introduction

The decision to commit to cell division involves the integration of growth signals within the cell cycle control system, leading to growth-dependent G1 cyclin synthesis. G1 events are perhaps best, though not thoroughly understood, in the budding yeast *Saccharomyces cerevisiae*. Here, G1 cyclin-associated Cdc28^Cdk1^ triggers decisive cell cycle commitment at the G1/S transition^1^. The irreversibility of cell cycle commitment is thought to be mediated by a positive feedback loop where G1-cyclin Cln1/2-Cdc28^Cdk1^ phosphorylate and inhibit Whi5, a transcriptional repressor of the G1 cyclins *CLN1* and *CLN2*^2–4^. When relieved of their repression, Cln1 and Cln2 activity results in nuclear export of Whi5, thereby stimulating more Cln1 and Cln2 expression and a burst of associated Cdc28^Cdk1^ activity^5,6^. In addition to driving an extensive G1 transcriptional program, or regulon, G1 cyclin Cdc28^Cdk1^ kinase also feeds into cell polarity, which at this point in the cell cycle is in a low activity state, reflected by the random organization of actin filaments. G1 cyclin-Cdc28^Cdk1^ ensures the activation of the Rho GTPase Cdc42 to a level compatible with rapid, stable polarity axis establishment and the polarization of the actin cytoskeleton, which supports the growth of a daughter cell called the bud^7^. This involves the nuclear export of the guanine exchange factor (GEF) for Cdc42, called Cdc24, into the cytoplasm^8,9^. Concomitantly, phosphorylation and inactivation of Cdc42 GTPase Activating Proteins (GAPs) including Rga2 occurs, together with phosphorylation of the GEF Cdc24 and Cdc42 scaffold-associated proteins, triggering polarity axis establishment^10–13^.

Importantly, G1 cyclin-Cdc28^Cdk1^ activity displays more than 10-fold substrate specificity towards these proteins compared to M-phase cyclin-Cdc28^Cdk1^ in vitro^11^. This substrate specificity, which is likely mediated via G1 cyclin docking motifs in substrates, could explain why budding in *S. cerevisiae* only occurs at G1/S phase in the cell cycle, and why it is not reinitiated subsequently as Cdc28^Cdk1^ activity rises during S-phase and mitosis^14,15^. The specific targeting of G1 cyclin activity towards the activation of the Cdc42 GTPase module is also borne out in vivo. *S. cerevisiae* strains in which all S- and M-phase cyclins are deleted can proliferate if a single engineered M-phase cyclin is provided, indicating that M-phase cyclins can drive S-phase events. However, the engineered M-phase cyclin cannot compensate for the loss of G1 cyclin activity, as it fails to drive polarity axis establishment and budding, indicative of G1 cyclin specificity towards polarity proteins in vivo^16^.

Early in G1, Cdc42 is localized on the plasma membrane due to prenylation and polybasic residues at its C-terminus^17,18^. These sequences anchor the protein to the plasma membrane, while the activation of the GTPase by its GEF Cdc24, and subsequent stabilization by the scaffold Bem1 drive its anisotropic activation during polarity axis establishment when G1 cyclin-Cdc28^Cdk1^ levels rise^19,20^. Cdc42 is not distributed homogeneously in the plane of the plasma membrane, but is rather clustered in diffraction-limited ensembles termed nanoclusters. Nanoclustering is a conserved feature of Rho GTPase organization in yeast, plants and mammals^21–24^. Moreover, it extends to the wider Ras-superfamily, where models propose that nanoclustering may digitize GTPase signaling by providing a high local concentration of GTPase module components^25^. During polarity establishment in budding yeast, anionic lipids including phosphatidylserine and phosphoinositols such as PI4P and PI(4,5)P2 are enriched in the interior leaflet of the plasma membrane where they become polarized^26–28^. These lipid species have also been demonstrated to play an important role in the nanoclustering of Ras and Rho-GTPases^22–24,29^. Multivalent electrostatic interactions between anionic lipids and basic residues in Bem1’s N-terminus and PX domain, together with the PH domain of Cdc24 are required for the recruitment of the proteins to the pole^30^. Here, Bem1 stimulates Cdc24 GEF activity and the generation of Cdc42-GTP^31,32^, which may trigger a positive feedback loop that recruits more Bem1 and Cdc24^19,33^. The outcome of this signaling is a sharp rise in the polarized activation of Cdc42, emanating from plasma membrane nanoclusters. Mutation of the basic residues in Bem1 and Cdc24 that mediate their interaction with anionic lipids results in a severe loss of the polarized organization of the GEF and scaffold from the plasma membrane at steady-state, together with defects in the size of Cdc42 nanoclusters and the ablation of PAK signaling^30^. However, the contribution of these multivalent lipid interactions to the dynamics of cell polarity and to ensuing cell cycle control has not been studied, and is the focus of this work.

Aberrant cell polarity and polarized growth are monitored by a cell cycle checkpoint in *S. cerevisiae* ^34,35^^,^. Defects in Cdc42 signaling or in actin organization early in the cell cycle result in Swe1^Wee1^ kinase activation, which phosphorylates and inhibits M-phase Cdc28^Cdk1^^36^, thus delaying nuclear division^37,38^. Swe1^Wee1^ activity interferes with both entry into mitosis and anaphase onset^39–43^. While Swe1^Wee1^ activation may provide a temporal window for polarity correction, it is unknown if Cdc28^Cdk1^ activity participates in the correction of polarity defects. The requirement for G1 cyclin-Cdc28^Cdk1^ in Cdc42 activation during polarity establishment in wild-type cells raised the possibility of its involvement in correcting polarity problems stemming from sub-critical Cdc42 activity.

In this work, the *bem1 cdc24* anionic lipid binding mutant, referred to as *bem1*^*lip*^*cdc24*^*lip*^, was used to investigate how interfering with Cdc42 nanoclustering affects the dynamics of cell polarity, enabling the identification of an unanticipated feedback relationship between GTPase signaling and G1 cyclins that is required for healthy cell proliferation.

## Results

### Polarity axis maintenance requires the lipid tethering of a Cdc42 GEF & scaffold to the plasma membrane

**Fig. 1:**
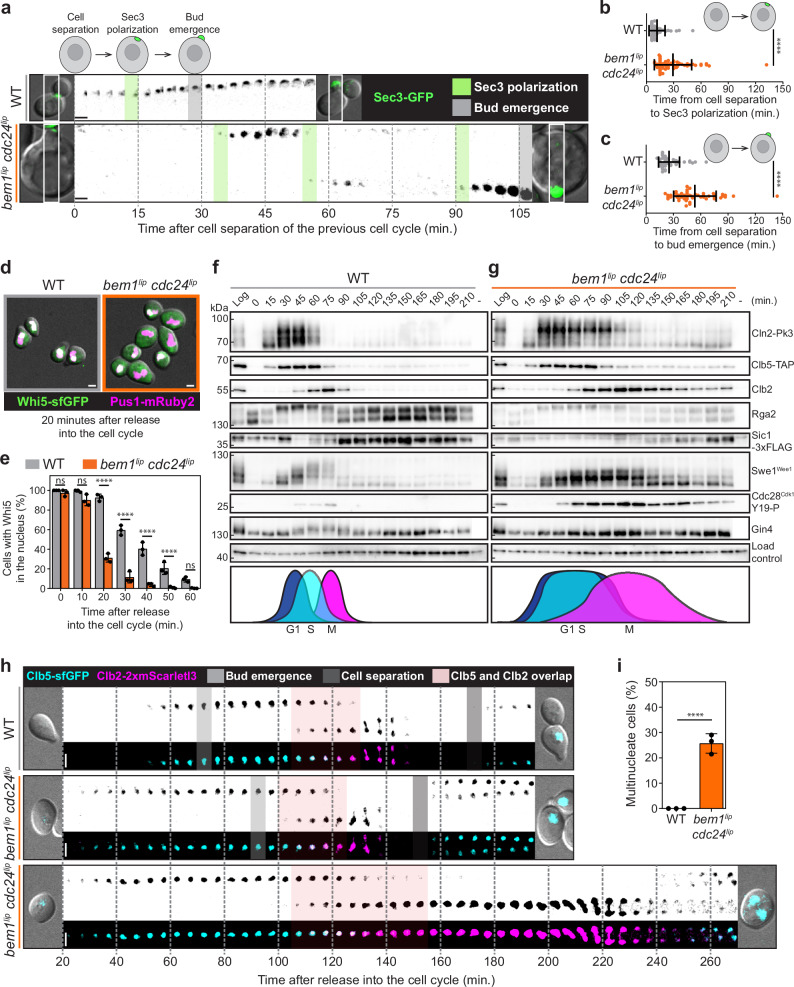
Lipid tethering defects in the *bem1*^*lip*^*cdc24*^*lip*^mutant impair polarity maintenance and the coordination of nuclear - cytoplasmic events. **a** Exocyst (Sec3-GFP) dynamics in WT and *bem1*^*lip*^ *cdc24*^*lip*^ cells. Images are maximum-intensity projected z-stacks. Time of cell separation in the previous cell cycle was used as reference (time = 0). Time of Sec3 polarization (green) and bud emergence (grey) are indicated. Number of biological replicates (*N*  =  4 for WT; *N*  =  8 for *bem1*^*lip*^ *cdc24*^*lip*^). Scale bars, 2 µm. **b** Time between cell separation and Sec3-GFP polarization in WT (*n* = 53 cells) and *bem1*^*lip*^ *cdc24*^*lip*^ (*n* = 61 cells). **c** Time between cell separation and bud emergence in WT (*n* = 52 cells) and *bem1*^*lip*^ *cdc24*^*lip*^ (*n* = 49 cells). **d** Whi5-sfGFP and Pus1-mRuby2 (nucleoplasm) 20 minutes after cell cycle entry. Scale bars, 2 µm. **e** Quantification of Whi5-sfGFP nuclear export (*n* > 100 per time point). Bars display mean +/- SD. Two-way ANOVA was performed (ns = not significant; ****p < 0.0001). Number of biological replicates (*N*  =  3, displayed as points). Exact *n* and *p* values are provided as a Source Data file. **f**, **g** Cell cycle progression in WT and *bem1*^*lip*^ *cdc24*^*lip*^ cells. Cells were arrested in G1 by alpha-factor treatment and released synchronously into the cell cycle. Images are representative of multiple biological replicates (*N*  =  3). The extended wave of cyclin expression in the mutant are displayed below in a schematic. **h** Dynamics of S-phase cyclin Clb5-sfGFP (top, cyan), M-phase cyclin Clb2-2xmScarletl3 (middle, magenta) and both (bottom) after arrest in G1 and release. Bud emergence, cell separation and cyclin overlap are highlighted. An example of cell that buds (middle) and that does not bud (bottom) is shown for the *bem1*^*lip*^ *cdc24*^*lip*^ mutant. Number of biological replicates (*N*  =  4 for WT; *N*  =  4 for *bem1*^*lip*^ *cdc24*^*lip*^). Scale bars, 2 µm. **i** Frequency of multinucleate cells in WT (*n* = 684 cells) and the *bem1*^*lip*^*cdc24*^*lip*^ mutant (*n* = 945 cells). Number of biological replicates (*N*  =  3 for WT; *N*  =  3 for *bem1*^*lip*^*cdc24*^*lip*^ (displayed as points)). In **b**, **c**,** i** bars display mean +/- SD, cells from multiple replicates have been pooled together and two-tailed unpaired *t-*tests with Welch’s correction were performed (****p  =  3.69e^−8^ (**b**); ****p  =  7.5e^−11^ (**c**); ****p  < 1e^−15^(**i**)). Source data are provided as a Source Data file.

To assess how anionic lipid tethering of the Cdc42 activators Cdc24 and Bem1 affect the dynamics of cell polarity, the exocyst subunit Sec3-GFP, whose localization depends on Cdc42 activity^44^, was monitored by time-lapse spinning disk microscopy in wild type and in *bem1*^*lip*^*cdc24*^*lip*^ mutant cells. In this mutant, charge-swap point mutations were introduced in anionic lipid binding sites in the Cdc24 PH domain, the Bem1 PX domain and in Bem1 N-terminal basic residues that were previously identified to be important for multivalent lipid interactions (Supplementary Fig. 1a)^30,45^. Cells were imaged from cell separation (defined from Sec3 fluorescence and DIC images as the time of daughter cell displacement, denoted time zero) to the time the daughter formed a medium-sized bud, which includes the period at the G1/S transition in which budding yeast establish the polarity axis used for growth and division (green shading in Fig. 1a). In wild type control cells, Sec3-GFP became polarized, delineating a single, stable polarity axis (Fig. 1a, upper panel and Supplementary Movie 1). In contrast, the time from cell separation to Sec3-GFP polarization was delayed in the *bem1*^*lip*^*cdc24*^*lip*^ mutant, indicative of a delay in polarity establishment (Fig. 1a, lower panel and Fig. 1b). In addition, the mutant exhibited a severe polarity maintenance defect, evident as an unstable pole (38% of cells). In the example shown, the cell made two attempts at Sec3-GFP polarization before successfully budding. As a result, the time from cell separation to bud emergence, and from Sec3-GFP polarization to bud emergence was delayed, or absent, in the mutant compared to wild type cells (Fig. 1c and Supplementary Fig. 1b, c and Supplementary Movie 2). Polarity establishment is a cytoplasmic event controlled by G1-cyclin Cdc28^Cdk1^ activity^11,46^. In order to determine if nuclear events driven by G1-cyclin Cdc28^Cdk1^ activity were also delayed in the mutant, we imaged Whi5-sfGFP, a transcriptional repressor acting at the G1/S transition whose nuclear export requires G1-cyclin Cdc28^Cdk1^ activity in a manner analogous to retinoblastoma (Rb) in metazoans^3^. Strikingly, Whi5-sfGFP nuclear export occurred more rapidly in a synchronized population of *bem1*^*lip*^*cdc24*^*lip*^ mutant cells compared to wild type cells, suggesting a loss of coordination between nuclear and cytoplasmic G1-cyclin Cdc28^Cdk1^–driven events in the polarity mutant (Fig. 1d, e). Whi5-sfGFP nuclear export also occurred more rapidly in the *bem1*^*lip*^*cdc24*^*lip*^ mutant cells compared to wild type cells in asynchronous cells (Supplementary Fig. 1d, e and Supplementary Movie 3).

In order to understand how nuclear and cytoplasmic events become uncoordinated in the *bem1*^*lip*^*cdc24*^*lip*^ mutant, progression through a single cell cycle was monitored in cells synchronized in G1 with alpha-factor (Supplementary Fig. 2a). The cell cycle in control cells was characterized by successive temporal waves of cyclin-Cdc28^Cdk1^ activity generated by rapid increases and subsequent decline in the cyclins Cln2 (G1), Clb5 (S-phase) and Clb2 (M-phase) (Fig. 1f). Abrupt Cln2 cyclin synthesis drives decisive polarity establishment and bud emergence (Supplementary Fig. 2b), via phosphorylation of G1 cyclin substrates including the Cdc42 GAP Rga2 (Fig. 1f). The characteristic switch-like properties of the cell cycle are reinforced by the rapid inhibition of Cdc28^Cdk1^ inhibitors Sic1, whose degradation is initiated at the G1/S transition in response to G1-cyclin Cdc28^Cdk1^ activity^47,48^, and Swe1^Wee1^, which undergoes multisite phosphorylation and inhibition at M-phase entry^49^. These mechanisms form part of a wider control network ensuring that cells enter mitosis decisively with low levels of inhibitory Cdc28^Cdk1^ Tyr19 phosphorylation and mitosis-specific phospho-regulation of proteins such as Gin4^50^.

In contrast, bud emergence was less switch-like in *bem1*^*lip*^*cdc24*^*lip*^ cells (Supplementary Fig. 2b), where live-imaging had shown the cells undergoing repeated attempts at polarity establishment. The G1 cyclin Cln2 exhibited an extended temporal window of expression (Cln2 started to decline after 45 minutes in control cells and after 90 minutes in *bem1*^*lip*^*cdc24*^*lip*^ cells, Fig. 1g) and exhibited rapid and quantitative hyperphosphorylation. Consistent with the extended window of Cln2 expression, a longer period of Rga2 phosphorylation and low Sic1 expression was observed. Both events require Cln-Cdc28^Cdk1^ activity, indicating that phosphorylated G1 cyclins observed in the mutant are active. All of the cyclins examined, Cln2, Clb5 and Clb2, displayed sluggish dynamics in the mutant, suggesting that the cell cycle’s switch-like properties were compromised. Moreover, three lines of evidence were indicative of Swe1^Wee1^-kinase activation in the mutant: (1) high levels of inhibitory Cdc28^Cdk1^ Tyr19 phosphorylation; (2) an intermediate phosphorylated species of Swe1^Wee1^ that has previously been identified as the active form of the kinase^49^, (3) the absence of mitosis-specific regulation of Gin4^51^.

To determine the consequences of Swe1^Wee1^ kinase activation on subsequent cell cycle progression, we monitored Clb5-sfGFP (S-phase) and Clb2-2xmScarlet-l3 (M-phase) cyclin dynamics during a cell cycle in wild type and *bem1*^*lip*^*cdc24*^*lip*^ mutant cells by dual-colour live-cell imaging (Fig. 1h). The single-cell data supported the conclusion from western blots that the expression of S- and M- phase cyclins were confined to a shorter, more discrete temporal window in wild type cells (Supplementary Movie 4), in contrast to the *bem1*^*lip*^*cdc24*^*lip*^ mutant, where loss of the cell cycle’s switch-like properties resulted in longer periods of cyclin expression (Fig. 1h and Supplementary Fig. 2c and d), and more extensive overlap of S- and M-phase cyclins (Fig. 1h and Supplementary Fig. 2e). Unbudded *bem1*^*lip*^ *cdc24*^*lip*^ mutant cells displayed prolonged Clb2-2xmScarletl3 expression during an extended mitotic delay preceding anaphase (Fig. 1h, and Supplementary Movie 4), consistent with elevated Swe1^Wee1^ kinase activity and high inhibitory Cdc28^Cdk1^ Tyr19 phosphorylation that was observed by western blotting. While Swe1^Wee1^-dependent Cdc28^Cdk1^ inhibition provided a temporal window for polarity correction, the transient nature of the delay meant that cells eventually underwent anaphase even if a daughter cell had not been generated. As a result, ~25% *bem1*^*lip*^ *cdc24*^*lip*^ mutant cells were multinucleate (Fig. 1i).

The strong polarity defects observed in synchronized *bem1*^*lip*^ *cdc24*^*lip*^ mutant cells were also observed in asynchronous cells. For example, the invariant order of Myo1 polarization, a type II myosin, then spindle pole body (SPB) separation and subsequent anaphase onset observed in WT cells was compromised in the mutant (Supplementary Fig. 3a–c, and Supplementary Movie 5). In addition, the mutant displayed a prolonged mitosis with high levels of Clb2 and the appearance of multinucleate cells, as observed in synchronized cells (Supplementary Fig. 3d, e; and Supplementary Movies 6 and 7).

In summary, the firm tethering of the Cdc42 regulators Bem1 and Cdc24 to anionic lipids resolves Cdc42 activation into a stable pole that is required for timely polarity establishment and maintenance. Compromising lipid binding results in an increased window of active G1 cyclin expression during the cell cycle and an accompanying loss of coordination between nuclear and cytoplasmic cell cycle events driven by G1 cyclin Cdc28^Cdk1^ activity. When all identified anionic lipid binding sites in Bem1 and Cdc24 were mutated, the resulting polarity defects also triggered a Swe1^Wee1^-dependent mitotic delay via inhibitory phosphorylation of Cdc28^Cdk1^ on Tyr19. These results underscore the importance of multivalent plasma membrane tethering of Cdc42 activators for normal cell cycle progression.

### The severity of the polarity defect governs the extent of the cell cycle delay

**Fig. 2:**
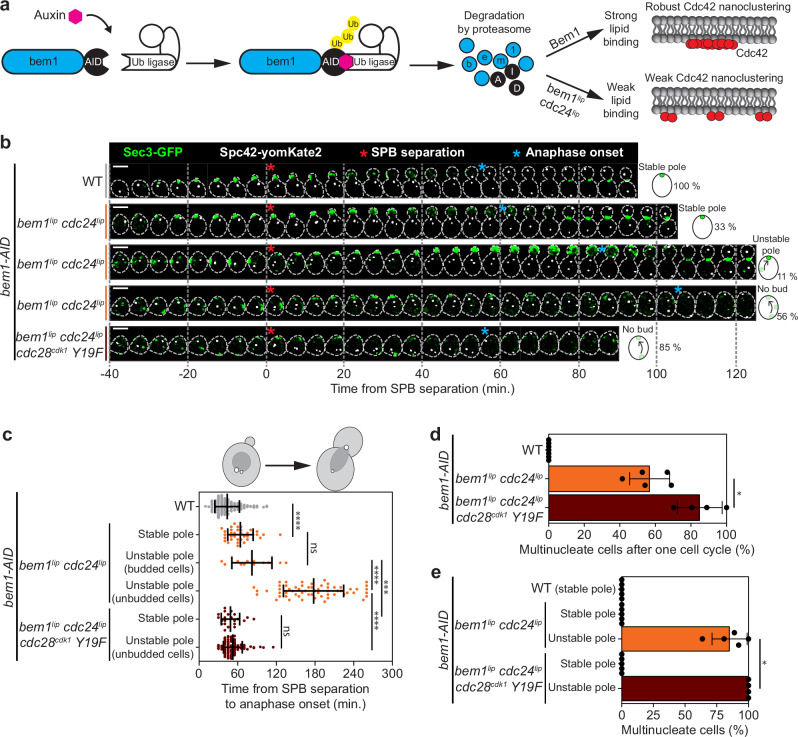
A conditional system indicates that the strength of the polarity defect in the *bem1*^*lip*^*cdc24*^*lip*^ mutant governs the extent of the Swe1^Wee1^ cell cycle delay. **a** A model showing the auxin-inducible degron (AID) system. After auxin (1-NAA) treatment, bem1-AID is degraded in *BEM1* control cells or in the *bem1*^*lip*^*cdc24*^*lip*^mutant. **b** Exocyst (Sec3-GFP, green) and spindle pole body (SPB, Spc42-yomKate2, white) dynamics. Cells were arrested in G1, treated with auxin for the final 30 minutes of alpha-factor arrest to degrade bem1-AID, then released into the cell cycle. Time 0 indicates SPB separation. Images are maximum-intensity projected z-stacks displaying SPB separation (red asterisk) and anaphase (blue asterisk). Schematics summarizing Sec3 dynamics and their quantification are shown on the right as a percentage. Scale bars, 5 µm. **c** Time between SPB separation and anaphase for WT (*n* = 179), *bem1*^*lip*^*cdc24*^*lip*^cells with stable Sec3 polarization (*n* = 35), unstable polarization that bud (*n* = 12), or unbudded (*n* = 60). Timing is also displayed for *bem1*^*lip*^*cdc24*^*lip*^*cdc28*^*cdk1*^ *Y19F* cells with stable (*n* = 22 cells) and unstable polarization (*n* = 95 cells). Bars display mean +/- SD. Two-tailed unpaired *t-*tests with Welch’s correction were performed (ns = not significant; ***p < 0.001; ****p < 0.0001). Exact *p* values are provided as a Source Data file. In (**b**, **c**), cells from multiple replicates have been pooled (*N*  =  6 for WT; *N*  =  5 for *bem1*^*lip*^*cdc24*^*lip*^; *N*  =  4 for *bem1*^*lip*^*cdc24*^*lip*^ *cdc28*^*cdk1*^ *Y19F*). **d** Frequency of multinucleate cells in WT (*n* = 179), *bem1*^*lip*^*cdc24*^*lip*^(*n* = 107) and *bem1*^*lip*^ *cdc24*^*lip*^*cdc28*^*cdk1*^ *Y19F* cells (*n* = 117). Bars display mean +/- SD. A Mann-Whitney (two-tailed unpaired) test was performed (*p = 0.016). **e** Frequency of WT cells (stable pole), *bem1*^*lip*^*cdc24*^*lip*^cells exhibiting a stable (*n* = 35), or unstable pole (*n* = 72) that become multinucleate. The frequency of *bem1*^*lip*^*cdc24*^*lip*^*cdc28*^*cdk1*^ *Y19F* cells exhibiting stable (*n* = 22 cells) or unstable Sec3 polarization (*n* = 95 cells) that become multinucleate are also indicated. Bars display mean +/- SD. A Mann-Whitney (two-tailed unpaired) test was performed (*p = 0.048). In (**d**, **e**), multiple biological replicates are displayed as points. Source data are provided as a Source Data file.

The *bem1*^*lip*^ *cdc24*^*lip*^ mutant had undergone generations of cell cycle progression during which the Swe1^Wee1^ checkpoint was active, raising the question of whether the observed phenotypes resulted from lipid binding defects, cell cycle checkpoint activation, or adaptation to sustained checkpoint activity (Supplementary Fig. 4a). A conditional system was therefore implemented to distinguish these possibilities. Bem1 was depleted using an auxin-inducible degron (AID) in *cdc24*^*lip*^ cells co-transformed with integrated versions of WT *BEM1* or *bem1*^*lip*^(Fig. 2a)^52,53^. Having verified that the conditional system provided temporal control of bem1-AID depletion (Supplementary Fig. 4b–e), the AID system was used to study the immediate consequences of loss of Bem1 and Cdc24 lipid binding.

The dynamics of cell polarity and cell cycle progression were monitored simultaneously after bem1-AID depletion in *BEM1* cells or the *bem1*^*lip*^ *cdc24*^*lip*^ mutant. The exocyst subunit Sec3-GFP was used as a marker of cell polarity, while Spc42-yomKate2, a spindle pole body component, was used as an indicator of cell cycle progression. Cells were synchronized in G1 and 1-NAA was added to degrade bem1-AID-6xFLAG 30-minutes prior to release into the cell cycle. Control cells entered the cell cycle with non-polarized Sec3-GFP that clustered as cells established a polarity axis. As cells budded, the Sec3-GFP signal was maintained in the growing bud (Fig. 2b). While 33**%** *bem1*^*lip*^ *cdc24*^*lip*^ mutant cells established a stable pole of Sec3-GFP similarly to control cells, albeit at a slower rate (Fig. 2b and Supplementary Fig. 5a, stable pole), the remaining cells either formed an unstable pole that was then corrected, enabling cells to bud (unstable pole), or formed an unstable pole that was not corrected, preventing budding (no bud). Thus, the mutant displayed either a weak, moderate or strong polarity defect.

The panel of polarity phenotypes provided an opportunity to relate the severity of the polarity defects to the temporal cell cycle delay, which could not be addressed previously with actin polymerization toxins in which F-actin organization is ablated^37,54^. The time from Sec3-GFP polarization to SPB separation was similar in the *bem1*^*lip*^ *cdc24*^*lip*^ mutant and in cells expressing wild type *BEM1* treated with 1-NAA, indicating that the SPB duplication cycle continues regardless of the severity of polarity defects (Supplementary Fig. 5b). Polarity defects and the expression of mitotic markers prior to bud emergence were observed in asynchronous cells, as was the case when the *bem1*^*lip*^ *cdc24*^*lip*^ mutant was constitutively expressed (Supplementary Fig. 5c–g). These results raised the question of how subsequent spindle dynamics would be affected. To address this, the time from SPB separation to anaphase onset was extracted in *BEM1* control cells and the three categories of mutant cells. In *BEM1* control cells this was 43 minutes, increasing to 64 minutes in *bem1*^*lip*^ *cdc24*^*lip*^ mutant cells that displayed a stable pole, and to 82 minutes in *bem1*^*lip*^ *cdc24*^*lip*^ mutants that displayed an unstable pole but eventually budded, and 177 minutes in mutant cells with an unstable pole that did not form a bud (Fig. 2b, c; and Supplementary Movie 8). Thus, the cell cycle delay correlated with the severity of the polarity defect incurred. We next tested if the cell cycle delay was dependent on Swe1^Wee1^, which inhibits Cdc28^Cdk1^ via phosphorylation on Tyr19 in response to signals including polarity defects^34^. In the *cdc28*^*cdk1*^*Y19F* mutant, the checkpoint is ablated because Cdc28^Cdk1^cannot be inhibited by phosphorylation. Consistently, when the *cdc28*^*cdk1*^ *Y19F* mutation was introduced into the *bem1*^*lip*^ *cdc24*^*lip*^ mutant, the cell cycle delay was abolished, regardless of whether cells budded or not, confirming that the cell cycle delay was Swe1^Wee1^-dependent (Fig. 2b, c). Moreover, the overall percentage of multinucleate cells increased from 56% in the *bem1*^*lip*^*cdc24*^*lip*^mutant to 85% in *bem1*^*lip*^ *cdc24*^*lip*^ *cdc28*^*cdk1*^ *Y19F* mutant cells (Fig. 2d). Remarkably, if the *bem1*^*lip*^*cdc24*^*lip*^*cdc28*^*cdk1*^ *Y19F* cells displayed any instability in polarity establishment, they invariably failed to bud and all of these cells became multinucleate (Fig. 2e). These observations underscore the protective role of Cdc28^Cdk1^ inhibitory phosphorylation when polarity defects are encountered. As an additional control, deletion of the *SWE1*^*WEE1*^ gene in the *bem1*^*lip*^*cdc24*^*lip*^mutant resulted in the appearance of multinucleate cells faster and to a higher extent than in the *bem1*^*lip*^*cdc24*^*lip*^mutant alone (Supplementary Fig. 5h). Collectively, these results demonstrate that polarity defects are sensed and translated into a Swe1^Wee1^-dependent cell cycle delay. The extent of the temporal cell cycle delay is proportional to the severity of the polarity problem encountered.

### The extended window of G1 cyclin expression is a direct response to polarity perturbations

**Fig. 3:**
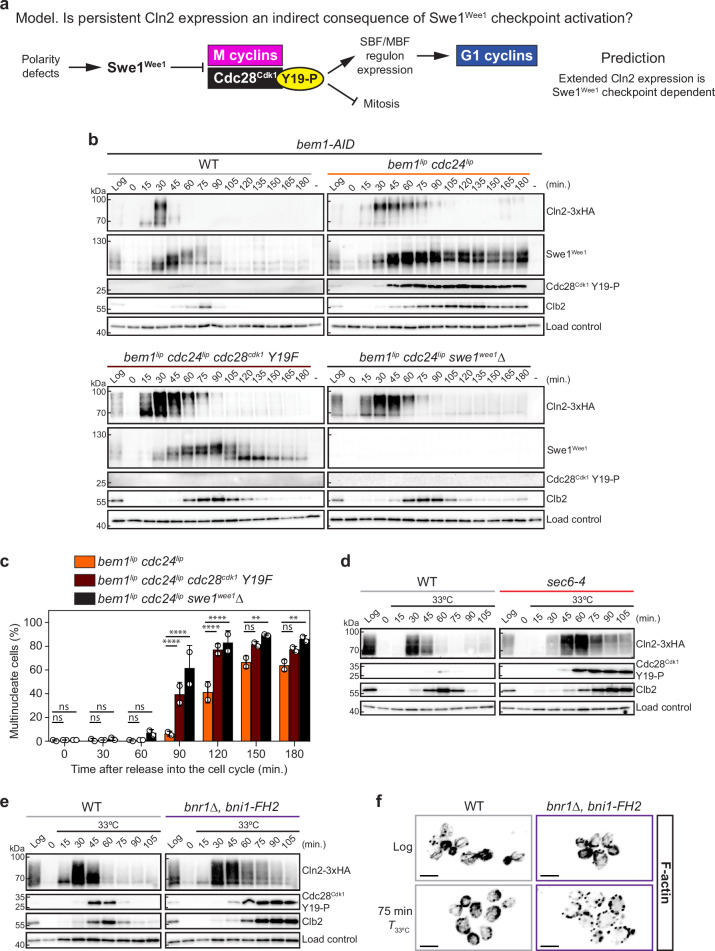
G1 cyclin hyperphosphorylation and stabilization in the *bem1*^*lip*^*cdc24*^*lip*^mutant are not indirect effects of Swe1^Wee1^ activation and are evident in other polarity mutants. **a** A model for G1 cyclin hyperphosphorylation and stabilization when polarity defects are encountered. When polarity defects arise, Swe1^Wee1^ kinase inhibits M cyclin-Cdc28^Cdk1^ and mitotic progression. SBF/MBF is reactivated, driving *CLN2* expression. In this model, prolonged Cln2 expression is a consequence of Swe1^Wee1^ checkpoint activity. **b** Cell cycle time-course of WT, *bem1*^*lip*^*cdc24*^*lip*^, *bem1*^*lip*^*cdc24*^*lip*^ *cdc28*^*cdk1*^ *Y19F* and *bem1*^*lip*^*cdc24*^*lip*^ *swe1*^*wee1*^*∆*. Cells were arrested in G1, treated with auxin for the final 30 minutes of alpha-factor arrest to degrade bem1-AID, then released into the cell cycle. Samples were collected at the indicated times. **c** The frequency of multinucleate cells was scored after DAPI staining (*n* > 100 cells per time point). Results from two biological replicates (displayed as points), bars correspond to mean +/- SD. A two-way ANOVA was performed (ns not significant; **p < 0.01*;* ****p < 0.0001). Exact *n* and *p* values are provided as a Source Data file. **d** Cell cycle time-course of WT and *sec6-4* cells after release into the cell cycle at the restrictive temperature (33 °C). **e** Cell cycle time-course of WT and *bnr1∆, bni1-FH2* cells after release into the cell cycle at the restrictive temperature (33 °C). **f** Maximum-intensity projected z-stacks of WT and *bnr1∆, bni1-FH2* cells from the time-courses shown in (**e**), where F-actin is visualized by Alexa-543 phalloidin staining. Fluorescence has been inverted. Scale bars, 5 µm. In (**b**,** d**,** e**), images are representative of multiple biological replicates (*N*  >  3 for each condition). Source data are provided as a Source Data file.

While a Swe1^Wee1^–dependent cell cycle delay provides a temporal window in which polarity defects can be corrected, it is unknown if Cdc28^Cdk1^ activity plays a role in the correction process itself. The observation that the G1 cyclin Cln2 was expressed during a longer temporal window in the *bem1*^*lip*^ *cdc24*^*lip*^ mutant is consistent with a model in which G1 cyclin Cdc28^Cdk1^ activity could contribute to polarity correction, since Cdc42 module components involved in GTPase activation are specific substrates of G1 cyclin Cdc28^Cdk1^ both in vitro and in vivo ^11,16^. However, the extended duration of Cln2 expression might alternatively be an indirect consequence of Swe1^Wee1^ checkpoint activation. This could occur because M-phase Cdc28^Cdk1^ activity usually represses G1 cyclin expression by inhibiting MBF/SBF that drive G1 cyclin transcription^55^. Swe1^Wee1^ checkpoint activity might therefore promote Cln2 expression by reactivating MBF/SBF (Fig. 3a). This model would predict that the extended expression of Cln2 in the *bem1*^*lip*^ *cdc24*^*lip*^ mutant would depend on Swe1^Wee1^ activity. This was tested by comparing Cln2 expression in *BEM1* control cells, *bem1*^*lip*^ *cdc24*^*lip*^*, bem1*^*lip*^ *cdc24*^*lip*^ *cdc28*^*cdk1*^ *Y19F* and *bem1*^*lip*^ *cdc24*^*lip*^ *swe1Δ* cells in which the Swe1^Wee1^ checkpoint is abrogated. Using the AID system, both Cln2 phosphorylation and its window of expression were similar in the *bem1*^*lip*^ *cdc24*^*lip*^ mutant, *bem1*^*lip*^ *cdc24*^*lip*^ *cdc28*^*cdk1*^ *Y19F* cells and *bem1*^*lip*^ *cdc24*^*lip*^ *swe1Δ* cells, although we occasionally also observed a sub-population of dephosphorylated Cln2 in checkpoint deficient cells (Fig. 3b and Supplementary Fig. 6a and b). Moreover, Swe1^Wee1^ checkpoint-deficient cells became multinucleate faster and to a greater extent than the *bem1*^*lip*^ *cdc24*^*lip*^ mutant alone within the single cell cycle of the experiment (Fig. 3c and Supplementary Fig. 6c). We conclude that Cln2 phosphorylation and its extended expression pattern are not indirect consequences of Swe1^Wee1^ checkpoint activity.

This left the alternative possibility to test: that G1 cyclin phosphorylation and extended expression may be a direct response to polarity defects that contribute to polarity correction. Reasoning that this may be a general response to defects in polarized growth, the dynamics of Cln2 expression were monitored in the *sec6-4*, an exocyst mutant that blocks the tethering of post-Golgi vesicles to the plasma membrane when shifted to the restrictive temperature, thereby preventing bud growth^56^. Wild type control and *sec6-4* cells were synchronized with alpha-factor and released into the cell cycle then shifted to 33 °C after 15 minutes. In control cells, Cln2 expression peaked 30 minutes after release into the cell cycle, dropped at 45 minutes and was largely absent at 60 minutes, whereas in *sec6-4* cells it peaked at 60 minutes and was still detected at 75 minutes. Moreover, Cln2 displayed a slower electrophoretic mobility in the *sec6-4* mutant than in control cells, reminiscent of its hyperphosphorylation observed in the *bem1*^*lip*^ *cdc24*^*lip*^ mutant (Fig. 3d). The *sec6-4* mutant also displayed elevated Cdc28^Cdk1^ Tyr19 phosphorylation in comparison to control cells, indicative of Swe1^Wee1^ activation. Importantly, this Tyr19 phosphorylation accumulated after the appearance of phosphorylated Cln2, indicating that it is a subsequent event. The formin mutant *bnr1∆ bni1-FH2* was a third polarity mutant in which Cln2 was phosphorylated and expressed for longer than control cells after a shift to the restrictive temperature (Fig. 3e)^57^. In this mutant, the actin cables used for post-Golgi vesicle transport to the plasma membrane are attenuated at the restrictive temperature, thus blocking polarized growth (Fig. 3f). In conclusion, G1 cyclins are phosphorylated and their expression is prolonged in response to polarity defects. This response is independent of the Swe1^Wee1^ checkpoint.

### G1 cyclin signaling contributes to Swe1^Wee1^ activity and corrects polarity errors

We next investigated the adaptive response that cells mount to polarity defects, in particular the mechanism of extended G1 cyclin expression. Cln1 and 2 are highly unstable proteins because they are substrates of the SCF^Grr1^ E3 ubiquitin ligase^58^. The extended expression of Cln2 observed in the *bem1*^*lip*^ *cdc24*^*lip*^ mutant might therefore reflect reduced degradation of the protein and/or increased mRNA levels. We first tested if Cln2 was stabilized in the mutant by measuring its half-life using a pulse of expression from the conditional *MET25* promoter (Supplementary Fig. 7a). Quantification of western blots indicated that the half-life of Cln2 was 22 minutes in the WT and 34 minutes in *bem1*^*lip*^ *cdc24*^*lip*^ cells, confirming its stabilization in the mutant (Supplementary Fig. 7b and c). As a control, we used a *cln2* mutant defective in SCF^Grr1^ recognition due to the mutation of 7 Cdk1^Cdc28^ consensus sites that function as a phospho-degron^59^. We confirmed that the half-life of this mutant, called *cln2*^*4T3S*^, was increased from 22 minutes for WT Cln2 to 56 minutes for *cln2*^*4T3S*^ (Supplementary Fig. 7d and e). We next tested if extended Cln2 expression in the *bem1*^*lip*^ *cdc24*^*lip*^ mutant also involved changes in mRNA levels by RT-qPCR. *CLN2* transcript levels peaked 30 minutes after entry into the cell cycle to a similar degree in WT and *bem1*^*lip*^ *cdc24*^*lip*^ cells (Supplementary Fig. 7f). However, whereas *CLN2* transcript levels dropped rapidly in WT cells, they remained higher in the *bem1*^*lip*^ *cdc24*^*lip*^ mutant. We conclude that the extended expression of Cln2 in the *bem1*^*lip*^ *cdc24*^*lip*^ mutant derives from stabilization of Cln2 protein and elevated *CLN2* mRNA.

Since rapid Cln2 hyperphosphorylation was the earliest cell cycle event in the polarity mutant to differ from control cells, we next sought to test its function. Cln2 phosphorylation has an established role in generating the phospho-degron that targets the protein for degradation by SCF^Grr1^ mentioned previously. This requires the phosphorylation of 7 minimal Cdc28^Cdk1^ consensus sites and is blocked in the *cln2*^*4T3S*^ mutant^59^. Phosphorylation of Cln2 may have additional functions; for example, it has been proposed to regulate Cln2 localization, but this function is less characterized and the number and location of sites are unknown^57^. Given that the extent of the Cln2 electrophoretic mobility shift was similar in WT and mutant cells, we posited that phosphorylation sites may be shared (Fig. 1f, g, 3b and Supplementary Fig. 6b). Therefore, to identify phosphorylation sites regulating Cln2 we searched for sites that were identified by mass spectrometry in large-scale studies and combined this data with our own mass spectrometry data of Cln2-Cdc28^Cdk1^ complexes that were purified from budding yeast using HA-tagged Cln2 (Supplementary Fig. 8a–d)^60,61^. In total, 24 phosphorylation sites were identified. Only two of these were Cdc28^Cdk1^ consensus sites involved in degradation, raising the question of what function the other sites serve. To begin to address this question we mutated the identified sites to alanine (*cln2-24A*) to block phosphorylation or aspartate (*cln2-24D*) to impart a negative charge similar to that of phosphorylation.

**Fig. 4:**
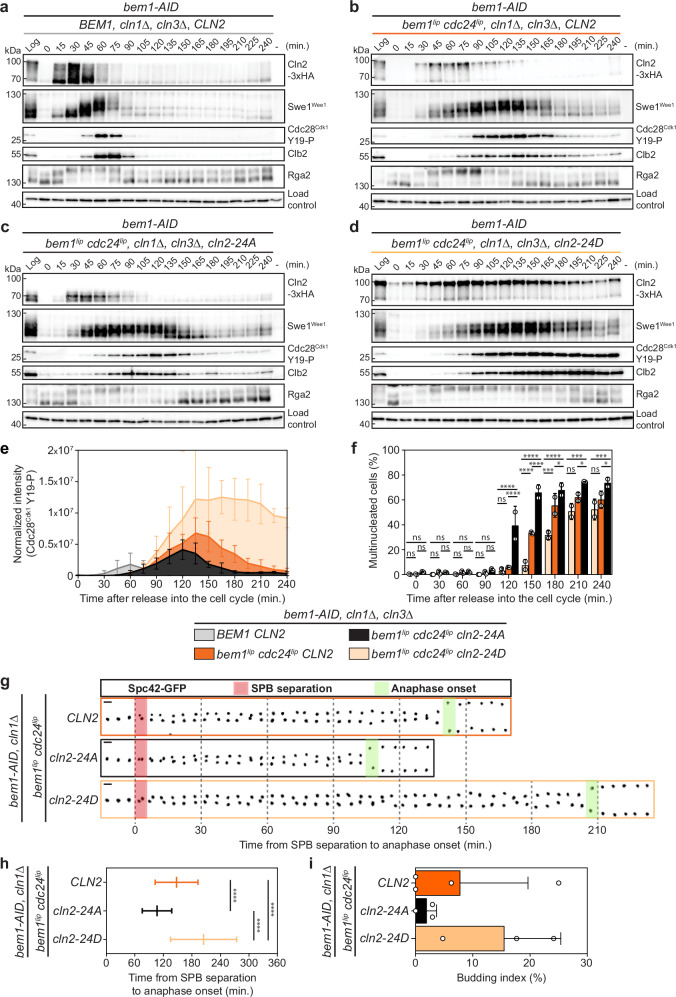
G1 cyclin signaling delays anaphase onset to protect cells from inappropriate nuclear division. **a** Cell cycle time-course of control *BEM1 CLN2* cells after arrest in G1, treatment with auxin for the final 30 minutes of synchronization, then release into the cell cycle. Samples were collected at the indicated times. **b** Analysis of *bem1*^*lip*^*cdc24*^*lip*^ *CLN2* cells. **c** Analysis of *bem1*^*lip*^*cdc24*^*lip*^ *cln2-24A* cells. **d** Analysis of *bem1*^*lip*^*cdc24*^*lip*^ *cln2-24D* cells. In (**a–d**), images are representative of multiple biological replicates (*N*  =  3 for each condition). **e** Normalized intensity of Cdc28 Tyr19 phosphorylation signal from 3 cell cycle time-courses (3 biological replicates). Lines and bars correspond to mean +/- SD. **f** Frequency of multinucleate cells from the time-courses displayed in (**a**–**d**) (*n* > 100 cells per time-point). Results from two biological replicates (displayed as points), bars correspond to mean +/- SD. A two-way ANOVA was performed (ns not significant; *p < 0.05*;* ***p < 0.001*;* ****p < 0.0001). Exact *n* and *p* values are provided as a Source Data file. **g** Spc42-GFP dynamics after arrest in G1, auxin treatment and release into the cell cycle. Maximum-intensity projected z-stacks of inverted fluorescence images. SPB separation is time 0 (pink) and anaphase onset is highlighted in green. Scale bars, 2 µm. **h** Time from SPB separation to anaphase onset from movies in (**g**). Lines and bars correspond to mean +/- SD. Two-tailed unpaired *t-*tests with Welch’s correction were performed (****p < 0.0001). Exact *p* values are provided as a Source Data file. Cells from multiple replicates have been pooled together to generate the graph (*n* = 56 cells for *bem1*^*lip*^*cdc24*^*lip*^ *CLN2*; *n* = 82 cells for *bem1*^*lip*^*cdc24*^*lip*^ *cln2-24A*; *n* = 67 cells for *bem1*^*lip*^*cdc24*^*lip*^ *cln2-24D*). **i** Frequency of budded cells from (**g**). Bars correspond to mean +/- SD (*n* = 56 cells for *bem1*^*lip*^*cdc24*^*lip*^ *CLN2*; *n* = 82 cells for *bem1*^*lip*^*cdc24*^*lip*^ *cln2-24A*; *n* = 67 cells for *bem1*^*lip*^*cdc24*^*lip*^ *cln2-24D*). Multiple biological replicates were carried out for the data displayed in panels **h** and **i** (*N*  =  4 for *bem1*^*lip*^*cdc24*^*lip*^ *CLN2*; *N*  =  3 for *bem1*^*lip*^*cdc24*^*lip*^ *cln2-24A; N*  =  3 for *bem1*^*lip*^*cdc24*^*lip*^ *cln2-24D* (displayed as points in **i**)). Source data are provided as a Source Data file.

In order to test the effect of Cln2 phosphorylation on cell cycle progression and Swe1^Wee1^ activity, we transformed the G1 cyclin constructs *CLN2-3xHA, cln2-24A-3xHA* and *cln2-24D-3xHA* into *BEM1* cells or *bem1*^*lip*^ *cdc24*^*lip*^ mutant cells in the AID system. Since the G1 cyclins Cln1, 2 and 3 play an essential but overlapping role in G1 progression, we also deleted *CLN1* and *CLN3* to avoid confounding effects deriving from redundancy. Cells were synchronized with alpha-factor and 30 minutes before release 1-NAA was added to degrade bem1-AID. Control experiments indicated that bem1-AID was degraded after 1-NAA addition (Supplementary Fig. 8e–h). In *BEM1 CLN2-3xHA* cells, successive distinct peaks of Cln2 then Clb2 expression were observed at 30 and 75 minutes after release into the cell cycle. We also observed that the Cdc42 GAP Rga2, a Cln2 substrate, became phosphorylated at 30 minutes, as Cln2 levels rose, displaying >50% dephosphorylation at 90 minutes (Fig. 4a). In *bem1*^*lip*^ *cdc24*^*lip*^ *CLN2-3xHA* cells, Cln2 was immediately phosphorylated, displaying a longer temporal window of expression. The phosphorylated Cln2 was active, since Rga2 remained phosphorylated for longer than in the *BEM1* control, peaking at 90 minutes and displaying >50% dephosphorylation at 135 minutes (Fig. 4b). As expected, the *bem1*^*lip*^ *cdc24*^*lip*^ mutant triggered Swe1^Wee1^ activity, evident by elevated Cdc28^Cdk1^ Tyr19 phosphorylation and by a prolonged peak of 2 C DNA content in FACS analysis (Supplementary Fig. 8i). The *bem1*^*lip*^ *cdc24*^*lip*^ *cln2-24A-3xHA* mutant strongly perturbed, but did not eliminate, Cln2 phosphorylation (Supplementary Fig. 8j). Rga2 phosphorylation was slightly advanced compared to *bem1*^*lip*^ *cdc24*^*lip*^ *CLN2-3xHA* cells, peaking at 75 minutes and displaying >50% dephosphorylation at 120 minutes (Fig. 4c). Levels of the M-phase cyclin Clb2 were also advanced, peaking at 90-105 minutes in *bem1*^*lip*^ *cdc24*^*lip*^ *cln2-24A-3xHA* cells compared to 135 minutes in *bem1*^*lip*^ *cdc24*^*lip*^ *CLN2-3xHA* cells, suggesting that perturbation of Cln2 phosphorylation advanced the onset of mitosis. Reduced Cdc28^Cdk1^ Tyr19 phosphorylation suggests that advanced mitosis in *cln2-24A-3xHA* cells was due to weakened Swe1^Wee1^ activity, which was evident from the accumulation of dephosphorylated, inactive Swe1^Wee1^.Conversely, Swe1^Wee1^ activity was elevated in *bem1*^*lip*^ *cdc24*^*lip*^ *cln2-24D-3xHA* cells where Cln2 phosphorylation was mimicked. Cdc28^Cdk1^ Tyr19 was extensively phosphorylated and Clb2 levels did not decline during the course of the experiment, indicative of a mitotic delay (Fig. 4d). These results were also evident from quantification of Cdc28^Cdk1^ Tyr19 phosphorylation in independent time-courses (Fig. 4e). Elevated Swe1^Wee1^ kinase activity in *bem1*^*lip*^ *cdc24*^*lip*^ *cln2-24D* cells followed a prolonged window of cln2-24D-3xHA expression (Fig. 4d). Moreover, Rga2 exhibited prolonged phosphorylation in *bem1*^*lip*^ *cdc24*^*lip*^ *cln2-24D-3xHA* cells, displaying >50% dephosphorylation only after 210 minutes. The robust phosphorylation of a Cln2 substrate in vivo indicates that the charge imparted by aspartate residues does not non-specifically impair Cln2 function. Since phosphorylation of the GAP Rga2 inactivates it, the results are also indicative of *bem1*^*lip*^ *cdc24*^*lip*^ *cln2-24D-3xHA* cells mounting a prolonged self-correcting response in an attempt to generate sufficient Cdc42-GTP to support a stable polarity axis.

Concomitantly, G1 cyclin phosphorylation triggered by polarity defects feeds into the strength of subsequent Swe1^Wee1^ activity: attenuating G1 cyclin phosphorylation weakened Swe1^Wee1^ activity, while mimicking G1 cyclin phosphorylation robustly strengthened it (Fig. 4e). We therefore assessed whether G1 cyclin phosphorylation and the level of subsequent Swe1^Wee1^ activity would protect cells against inappropriate nuclear division. To do so, the frequency of multinucleate cells over time was quantified as a biological readout of Swe1^Wee1^ activity. In the *bem1*^*lip*^ *cdc24*^*lip*^ *cln2-24A* mutant, the appearance of multinucleate cells occurred faster and in greater numbers, consistent with weakened Swe1^Wee1^ activity (Fig. 4f). Conversely, in *bem1*^*lip*^ *cdc24*^*lip*^ *cln2-24D* cells, multinucleate cells appeared at a slower rate, consistent with heightened Swe1^Wee1^ activity. Kymographs of SPB dynamics using Spc42-GFP as a marker confirmed that the time from SPB separation to anaphase onset was reduced in *bem1*^*lip*^ *cdc24*^*lip*^ *cln2-24A* cells compared to *CLN2* cells, while being delayed in the *bem1*^*lip*^ *cdc24*^*lip*^ *cln2-24D* mutant (Fig. 4g, h and Supplementary Movie 9). We conclude that phosphorylation of the G1 cyclin Cln2 that occurs extensively in the *bem1*^*lip*^ *cdc24*^*lip*^ mutant is involved in relaying signals to Swe1^Wee1^ that slow nuclear division, providing a temporal window for polarity correction. G1 cyclin activity also contributes directly to error correction by phosphorylating GTPase components such as the GAP Rga2. This was borne out quantitatively by comparing the percentage of budded *bem1*^*lip*^ *cdc24*^*lip*^ mutant cells in the G1 cyclin phospho-mutants in the first cell cycle after auxin addition (Fig. 4i). Of these mutants, the *bem1*^*lip*^ *cdc24*^*lip*^ *cln2-24D* cells that displayed the longest period of GAP inactivation due to Rga2 phosphorylation also displayed more budded cells (16%) compared with *bem1*^*lip*^ *cdc24*^*lip*^ *cln2-24A* cells (2%), suggesting that G1 cyclin phosphorylation was somehow involved in polarity correction. Before addressing the mechanism by which G1 cyclin phosphorylation corrects polarity errors, we tested whether the expression of *cln2* phosphorylation mutants may have a dominant effect in WT *BEM1* cells. The stability of Cln2, cln2-24A and cln2-24D was similar in WT cells (Supplementary Figs. 9a–c). However, cln2-24D expression was prolonged during the cell cycle in WT (Supplementary Fig 10a, b). Moreover, prolonged expression of the G1 cyclin in *cln2-24D* cells resulted in mildly elevated Cdc28^Cdk1^ Tyr19 phosphorylation and a 15-minute delay in Clb2 degradation, suggesting mild Swe1^Wee1^ activation (Supplementary Fig 10a, b). This effect, which was not due to a delay in budding, was also evident by FACS analysis as a prolonged 2 C DNA content peak in *cln2-24D* cells (Supplementary Fig 10c, d). In addition, the mutant cells were more ellipsoid than WT, a phenotype that is also observed in Cdc42 GAP mutants (Supplementary Fig 10e, f). Collectively, this suggests that cln2-24D may directly activate Swe1^Wee1^ and induce a mild mitotic delay.

### Polarity defects trigger the spatial reconfiguration of G1 cyclin activity

**Fig. 5:**
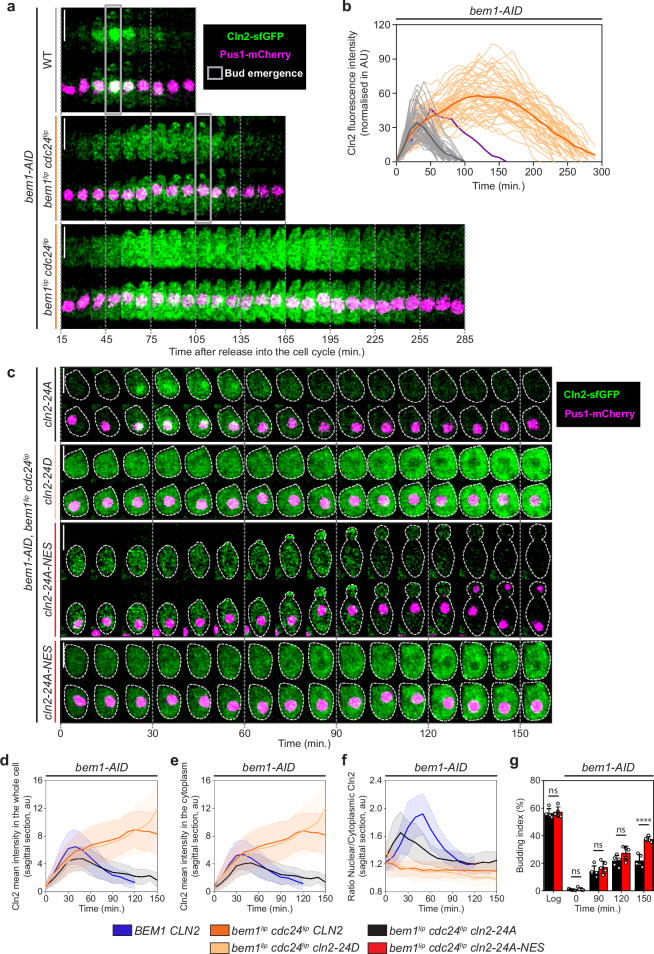
The *bem1*^*lip*^*cdc24*^*lip*^mutant triggers an adaptive response that augments cytoplasmic G1 cyclin levels. **a** Cln2-sfGFP (green) and Pus1-mCherry (magenta, nucleoplasm) dynamics were visualized after G1 arrest, auxin treatment and release into the cell cycle (used as reference time 0). Images are maximum-intensity projected z-stacks where bud emergence is indicated (grey rectangle). An example of a *bem1*^*lip*^*cdc24*^*lip*^ cell that buds (middle) and one that does not bud is shown (bottom). Scale bars, 5 µm. **b** Quantification of Cln2 fluorescence intensity in each time frame in WT cells (*n* = 47) and *bem1*^*lip*^*cdc24*^*lip*^ mutant cells (*n* = 48). Each line is an individual cell, and bold lines display the mean of WT and *bem1*^*lip*^*cdc24*^*lip*^ cells. The purple line displays Cln2 dynamics in a *bem1*^*lip*^*cdc24*^*lip*^ cell that buds, shown in (**a**). Number of biological replicates (*N*  =  3 for WT; *N*  =  3 for *bem1*^*lip*^*cdc24*^*lip*^). **c** Cln2-sfGFP (green) and Pus1-mCherry (magenta) dynamics after G1 arrest, auxin treatment and release into the cell cycle. Images are maximum-intensity projected z-stacks. Scale bars, 5 µm. Two examples of *bem1*^*lip*^*cdc24*^*lip*^ *cln2-24A-NES* (Nuclear Export Signal) are shown (a budded and an unbudded cell). **d** Quantification of Cln2 mean fluorescence intensity in the whole cell. **e** Quantification of Cln2 mean fluorescence intensity in the cytoplasm. **f** The ratio of nuclear to cytoplasmic Cln2 is displayed. In (**d**–**f**), cells from multiple replicates have been pooled together to generate the graphs (*n* = 47 for WT; *n* = 47 for *bem1*^*lip*^*cdc24*^*lip*^; *n* = 51 for *bem1*^*lip*^ *cdc24*^*lip*^ *cln2-24A*; *n* = 50 for *bem1*^*lip*^ *cdc24*^*lip*^ *cln2-24D*). Number of biological replicates (*N*  =  6 for WT, *N* = 5 for *bem1*^*lip*^ *cdc24*^*lip*^; *N*  =  7 for *bem1*^*lip*^ *cdc24*^*lip*^ *cln2-24A; N*  =  6 for *bem1*^*lip*^ *cdc24*^*lip*^ *cln2-24D*). Lines display mean +/- SD. **g** Frequency of budded cells after release into the cell cycle. Log sample was taken before alpha-factor treatment and bem1-AID degradation. Bars correspond to mean +/- SD. Results from five biological replicates (displayed as points, *n* > 200 cells per time point). A two-way ANOVA was performed (ns = not significant; ****p < 0.0001). Exact *n* and *p* values are provided as a Source Data file.

The Cdc28^Cdk1^ substrates that regulate polarity such as Rga2 are localized in the cytoplasm, whereas Cln2 localises to both the nucleus and cytoplasm. Since the nucleocytoplasmic partitioning of Cln2 has been linked to its phosphorylation, we next monitored Cln2 dynamics during the adaptive response to polarity defects^62,63^^,^. This was not previously possible due to the slow maturation kinetics of GFP relative to the rapid-proteolysis and low expression levels of Cln2. To overcome these challenges, Cln2 was tagged at its endogenous locus with sfGFP in wild type control and *bem1*^*lip*^ *cdc24*^*lip*^ mutant cells and imaged by time-lapse spinning-disk confocal microscopy. The nucleoplasmic protein Pus1 was also tagged as a nuclear marker. The dynamics of Cln2-sfGFP were monitored in individual cells after synchronous release into the cell cycle. Cln2-sfGFP expression in control cells was evident in the cytoplasm and prominently in the nucleus for around 45 minutes, consistent with the temporal expression observed in cell populations by western blotting (Fig. 5a and Supplementary Movie 10). In contrast, nuclear enrichment of Cln2-sfGFP expression was not observed in *bem1*^*lip*^ *cdc24*^*lip*^ mutant cells, which instead displayed a prominent and sustained burst of cytoplasmic Cln2. In a *bem1*^*lip*^ *cdc24*^*lip*^ mutant cell that formed a bud, Cln2-sfGFP was rapidly degraded after bud emergence, indicating that successful polarization and budding is linked to Cln2 degradation (Fig. 5a middle panels and Supplementary Movie 10). However, the majority of *bem1*^*lip*^ *cdc24*^*lip*^ mutant cells did not form a bud and these cells displayed a continuous increase in cytoplasmic Cln2-sfGFP expression that eventually declined (Fig. 5a lower panels, b and Supplementary Movie 10). Fluorescence intensity quantification indicated that *bem1*^*lip*^ *cdc24*^*lip*^ mutant cells expressed higher global levels of Cln2-sfGFP at steady-state, as well as displaying higher fluorescence intensity in the cytoplasm and a lower nuclear:cytoplasmic ratio than control *BEM1* cells (Supplementary Figs. 11a-c). This spatial reconfiguration of G1 cyclin activity from the nucleus to the cytoplasm in response to polarity defects was also evident by monitoring the dynamics of cln2-sfGFP in the phosphorylation mutants. When polarity defects were induced in *bem1*^*lip*^ *cdc24*^*lip*^ cells, *cln2-24A-sfGFP* mutants exhibited more prominent nuclear fluorescence than in *bem1*^*lip*^ *cdc24*^*lip*^ cells alone, consistent with the non-phosphorylated form of the protein being enriched in the nucleus (Fig. 5c and Supplementary Movie 11). Conversely, in *bem1*^*lip*^ *cdc24*^*lip*^ cells, *cln2-24D-sfGFP* displayed an increased, sustained burst of cytoplasmic fluorescence and partial exclusion from the nucleus that was evident from quantification (Fig. 5c–f). These results suggest that Cln2 phosphorylation may play a role in its nucleo-cytoplasmic partitioning. This would predict that forcing nuclear-enriched cln2-24A into the cytoplasm may be sufficient to alleviate polarity defects, which we tested by appending an NES signal to *cln2-24A-sfGFP* in *bem1*^*lip*^ *cdc24*^*lip*^ cells. Live cell imaging revealed that the protein no longer accumulated in the nucleus and that the NES was sufficient to restore budding or to initiate the polarized accumulation of cln2 in cells that attempted budding (Fig. 5c middle and bottom panels; Supplementary Movie 12). As a consequence, the percentage of budded cells increased after adding an NES to cln2-24A when cells were synchronously released into the cell cycle (Fig. 5g).

We next sought to distinguish phosphorylation sites that were important for localization from those that stabilized Cln2. To do so, *bem1*^*lip*^ *cdc24*^*lip*^ cells were transformed with *CLN2-sfGFP* or a mutant called *cln2-22A* in which only non-Cdk1 consensus sites (non-SP/TP) were mutated to alanine. Cells were given a pulse of G1 cyclin-sfGFP expression using the conditional *MET25* promoter. Whereas *CLN2-sfGFP* accumulated in the cytoplasm in the polarity mutant, *cln2-22A-sfGFP* displayed nuclear enrichment, indicating that phosphorylation of these non-Cdk1 consensus sites are important for its cytoplasmic accumulation in response to polarity defects (Supplementary Fig. 12a; Supplementary Movie 13). Conversely, the mutation of only Cdk1 consensus sites that are known to stabilize Cln2 resulted in strong nuclear accumulation of the resulting *cln2*^*4T3S*^*-sfGFP* in WT *BEM1* cells, in addition to enrichment in the growing bud where Cdc28^Cdk1^ drives polarized growth. In *bem1*^*lip*^ *cdc24*^*lip*^ cells, *cln2*^*4T3S*^*-sfGFP* was rerouted to the cytoplasm over time, presumably via the phosphorylation of the 22 non-Cdk1 consensus sites that we identified as being important for the G1 cyclin reponse to polarity defects (Supplementary Fig. 12b; Supplementary Movie 14). The *cln2*^*4T3S*^ mutant supported Cln2 function in polarized growth, and, like *cln2-24D*, generated cells that were more ellipsoid than WT *CLN2* (Supplementary Fig. 12c–e). The key role that different phosphorylation sites play in the nucleo-cytoplasmic routing of Cln2 was borne out by quantitating the ratio of nuclear/cytoplasm signal in *cln2*^*4T3S*^ and *cln2-22A* mutants (Supplementary Fig. 12f, g). Moreover, the reconfiguration of G1 cyclin localization in *bem1*^*lip*^ *cdc24*^*lip*^ cells may be an adaptive response to diverse polarity defects as we also observed cytoplasmic accumulation of Cln2 in *sec6-4* cells that are defective in post-Golgi vesicle tethering (Supplementary Fig. 13a–e).

## Discussion

The nanoclustering of proteins on the plasma membrane was previously proposed to digitize signaling, contributing to the decisiveness of Ras and Rho GTPase responses^25^. In this study, interfering with Cdc42 nanoclustering on the plasma membrane by ablating multivalent anionic lipid interactions underscored its importance for polarity dynamics in vivo; polarity establishment was delayed and was not decisive, but rather vacillated, displaying strong defects in maintenance. However, the loss of decisiveness ensuing from the lipid tethering defects of Cdc42 activators extended beyond GTPase signaling. Unexpectedly, the switch-like properties of the entire cell cycle control system, which is usually characterized by successive temporal waves of cyclin activity, became compromised.

**Fig. 6:**
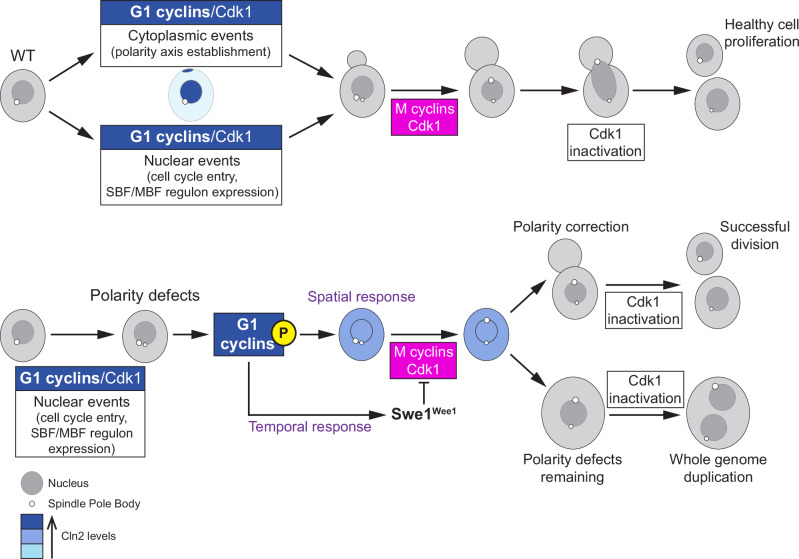
Polarity defects trigger an adaptive response that induces the rerouting of G1 cyclins to the cytoplasm and delays cell cycle progression. Schematic model of key cell cycle events related to G1 in wild type cells (upper panel) and the corrective events that are triggered in response to polarity defects including the outcome of these events (lower panel). G1 cyclin localization in the nucleus and cytoplasm is denoted in blue. In this model, G1 cyclins are phosphorylated in response to polarity defects. This signal contributes to healthy cell proliferation in two ways: a temporal response delays nuclear division via Swe1^Wee1^ kinase activity while a spatial response results in G1 cyclin enrichment in the cytoplasm. However, this strategy has a cost: if the polarity defect is too severe to be corrected, high G1-cyclin levels enforce inexorable cell cycle commitment in the absence of a daughter cell, generating multinucleate cells.

The loss of the cell cycle’s switch-like characteristics is derived from the activation of an adaptive response that cells mount to correct polarity defects. This involved a prolonged burst of cytoplasmic G1 cyclin expression that phosphorylated and inactivated a Cdc42 GAP, thus boosting Cdc42-GTP levels. This spatial response also fed into a temporal response that activated Swe1^Wee1^ kinase activity, delaying nuclear division via M-phase cyclin stabilization until polarity was corrected (Fig. 6). Interfering with either protective mechanism compromised euploidy. The study also illustrates how G1 cyclins not only trigger an essential cell cycle event - polarity establishment, but also monitor its successful completion. This requires G1 cyclins to be embedded within feedback networks that adjust cyclin activity and localization until the events that they monitor are executed appropriately. The strategy of upregulating the activator of a system in response to a deficit of the system’s output seems ingenious. The necessity for G1 cyclin upregulation to rectify polarity defects may derive from the specificity that G1 cyclins display for Cdc42 module components^11,16^. However, the cost of this strategy is that G1 cyclin upregulation commits cells to one cycle of cell division. This means that even if the cell cannot correct the problem, it will nevertheless eventually undergo nuclear division, generating a multinuclear cell. This underscores the perils of passage through G1 and irreversible cell cycle commitment. It may also explain the high frequency with which G1 control is found to be aberrant in tumours^64^. The short-term response identified in this study occurs in the cell cycle during which the polarity problem is encountered. However, in the longer-term, cells in which the scaffold *BEM1* has been deleted adopt a different strategy to mitigate the ensuing deficit of active Cdc42. These cells down-regulate inhibitors of Cdc42 due to a selective pressure for the mutation of GAPs, instead of upregulating an activator, highlighting the remarkable capacity of GTPase signaling modules to rapidly adapt to perturbations^65^. The present study illustrates how these perturbations to GTPase signaling extend beyond cell polarity to affect the cell cycle control network, whose timely response is essential to coordinate cell polarity and cell cycle progression, a requirement for healthy cell proliferation.

## Methods

### Plasmid construction

Plasmid names are provided in Supplementary Table 1. To construct AID strains, pDM1052 was generated by digesting pDM443 (pRS303) with XhoI and SacI and ligating *pBEM1-bem1 bc-14E + px (K338M, K348A, R349A & R369A)-Pk3*. pDM1053 was generated by ligating *pBEM1-BEM1-Pk3* (XhoI-EcoRI) into pRS303. pDM1052 and pDM1053 were integrated at the *his3* locus by digesting with NheI.

Synthesized gene fragments encoding *cln2-24A-3xHA* (*S233A, S236A, S239A, T242A, T378A, S380A, T381A, S383A, S385A, S389A, S391A, T392A, S393A, S396A, S399A, S400A, S401A, S403A, T468A, S472A, T476A, S528A, S529A, S530A)* or *cln2-24D-3xHA (S233D, S236D, S239D, T242D, T378D, S380D, T381D, S383D, S385D, S389D, S391D, T392D, S393D, S396D, S399D, S400D, S401D, S403D, T468D, S472D, T476D, S528D, S529D, S530D)* containing NdeI and SpeI sites were cloned into pDM64 (*pCLN2-CLN2-3xHA LEU2 (INT)*). The *cln2-3xHA* constructs were then sub-cloned into pRS406 (*URA3*) to generate pDM1085 (*pCLN2-CLN2-3xHA*), pDM1087 (*pCLN2-cln2-24A-3xHA*) and pDM1090 (*pCLN2-cln2-24D-3xHA*) and amplified using oligonucleotides that targeted integration at the *CLN2* locus. Integration was verified by PCR and sequencing. Gene synthesis was used to generate *cdc28*^*cdk1*^ *Y19F* mutants. *pCDC28-cdc28*^*cdk1*^*Y19F-cdc28term::natNT2* was synthesized (pDM1091), then digested with BglII and EcoRI to change the selection marker, generating pUC57-*pCDC28-cdc28*^*cdk1*^*Y19F-CDC28term::kanMX6* (pDM1092). pDM996 (pFA6a-*GAGAGA-mRuby2::HIS3)* was generated by replacing GFP in pFA6a-*GFP(S65T)::HIS* with GAGAGA-mRuby2 using PacI and AseI sites. pDM1064 and pDM1065 were generated by replacing *HIS3* in pFA6a-*GFP(S65T)::HIS3* with *CaURA3* and *natNT2*, respectively, after digestion with PmeI and BglII. pDM1140 (pFA6a-*mScarletl3-mScarletl3::HIS3*) was generated by replacing GFP in pFA6a-*GFP(S65T)::HIS3* sequentially with *mScarletl3* coding regions. Plasmids were checked by sequencing.

### Yeast strains and growth conditions

Strain names and genotypes are provided in Supplementary Table 2. One-step PCR-based gene replacement was used for tagging at endogenous loci. To check for integration at the desired locus, flanking oligonucleotides were designed and used with untagged control DNA. Cells were grown in rich medium (YPD) or synthetic medium at 25 °C and maintained in semi-logarithmic growth phase (OD_600 nm_ < 0.8 ml^−1^).

AID strains were generated as follows: the pTIR1 plasmid was digested using PmeI, to release TIR1 for integration at the *LEU2* locus^52^. In a second step, *BEM1* was tagged by homologous recombination (*BEM1-AID-6xFLAG::hphNT1*) at the endogenous *BEM1* locus. Finally, a WT version of *BEM1* (*his::BEM1-Pk3::HIS3*) or a mutant version of *bem1*^*lip*^ (*his3::BEM1p-bem1 bc-14E + px (K338M, K348A, R349A & R369A)-Pk3::HIS3*) was integrated at the *his3* locus. Transformants were tested for 1-NAA sensitivity and verified by PCR and DNA sequencing.

### Testing G1 arrest of cells with alpha-factor - “halo assays”

Cells were grown at 25 °C to semi-logarithmic growth phase. Two dilutions of cells were spread on YPD plates. Sterile filter paper was then applied with different concentrations of alpha-factor (0.5 mg.ml^−1^, 0.05 mg.ml^−1^ or 0.005 mg.ml^−1^). After 2-days at 25 °C, halos of growth inhibition due to cell cycle arrest were observed around the filter paper (Supplementary Fig. 2a).

### Cell cycle time-courses

Cells were grown at 25 °C until early-logarithmic phase, then diluted to an OD_600 nm_ of 0.3 ml^−1^. Cells were then synchronized by adding alpha-factor to 7 µg.ml^−1^ (or 0.5 µg.ml^−1^ for *bar1∆* cells) for 3-h at 25 °C. After washing cells in YPD using a vacuum pump attached to a manifold with a 0.22 µm nitrocellulose membrane without allowing them to dry, cells were released synchronously into the cell cycle. Cells were then incubated at 25 °C with agitation, and samples were taken every 15 minutes. To ensure that all cells completed a single cycle, alpha-factor (15 µg.ml^−1^) was added back to cells at 50 minutes to re-arrest them at the end of a single cell cycle. When using the AID system, bem1-AID was degraded by the addition of 0.5 mM 1-NAA, 30 minutes before washing away the alpha-factor. The same concentration of 1-NAA was added throughout the rest of the experiment to degrade any newly synthesized bem1-AID.

### Determination of protein stability

Cells expressing *MET25p-3xHA-CLN2* at the endogenous locus were grown at 25 °C in YPD supplemented with 0.08 g.l^−1^ methionine until mid-logarithmic phase (Supplementary Fig. 7a–c). Cells were then washed and resuspended in minimal medium lacking methionine for 2 h to induce expression from the *MET25* promoter, before YPD + 0.08 g.l^−1^ methionine was added to repress expression. Cell lysates were analyzed by immunoblotting using anti-HA antibodies. When using the AID system, bem1-AID was degraded by the addition of 0.5 mM 1-NAA, 30 minutes before washing away the methionine. The same concentration of 1-NAA was added throughout the rest of the experiment to degrade newly synthesized bem1-AID.

“Gal-shut-off” experiments were used to determine protein stability (Supplementary Fig. 7d, e and Supplementary Fig. 9). Cells were grown in YEP medium supplemented with 2% ethanol + glycerol at 25 °C until mid-logarithmic phase then diluted to an OD_600 nm_ of 0.4 ml^−1^. Galactose was then added to a final concentration of 2 % to induce expression from the *GAL1* promoter. After 60 minutes of gal-induction, cells were collected by centrifugation and resuspended in YEP with 2 % dextrose to repress expression.

### Western blotting

Samples for western blotting were prepared by collecting 1 OD_600 nm_ of mid-logarithmic phase cells, adding glass beads and flash freezing in liquid nitrogen. Some specific proteins required a different volume of cells to be frozen, which varied according to the sensitivity of the antibody being used for detection and protein abundance (0.5 OD_600 nm_ units for cyclins, 1.5 OD_600 nm_ units to detect phosphorylation on Tyr19 of Cdc28, or 2 OD_600 nm_ units for Rga2). Samples were vigorously agitated in 100 µL SDS sample buffer (65 mM Tris pH 6.8, 3% SDS, 10 % glycerol, 5 % β-mercaptoethanol, 100 mM β-glycerolphosphate, 50 mM NaF) supplemented with 1 mM fresh PMSF. Samples were immediately boiled and analyzed by SDS–PAGE, western blotting, and probed with appropriate antibodies. The primary antibodies used were anti-HA (12CA5), anti-TAP (IgG peroxidase, Jackson) anti-FLAG (M2), anti-c-MYC (9E10), anti-V5 (Pk3), anti-Clb2, anti-Swe1, anti-Gin4, anti-Ade13 (used as a loading control), anti-Rga2, anti-Tyr19P (phospho-cdc2 (Y15) Cell Signaling Technology). In figures, logarithmically-growing samples prior to synchronization are labelled “Log”, whereas negative controls (gene deletions or untagged controls) are labelled with a minus sign (“-“).

Where anti-Cdc28^Cdk1^ phospho-Tyr19 chemiluminescence signals were quantified, the same membrane was used for quantification and for the measurement of a loading control (anti-Ade13). Non-specific background was removed globally using ImageLab software, and volume tools were used to measure adjusted signal intensity before normalizing anti-Cdc28^Cdk1^ phospho-tyr19 to the anti-Ade13 loading control. All uncropped blots and molecular weight markers are available in Source Data.

### Cell imaging

Live cells to be imaged were grown at 25 °C in synthetic medium without tryptophan to semi-logarithmic phase and treated, or not, with alpha-factor and 1-NAA as indicated in figure legends. Cells were isolated for washing using a vacuum pump attached to a manifold with a 0.22 µm nitrocellulose membrane before being mounted on 0.5 % agar pads and sealed with halo-carbon oil for imaging. In Supplementary Fig. 12, cells expressing Cln2-3xHA-sfGFP under control of the *MET25* promoter were arrested in G1, and treated with auxin for the final 30 minutes of the alpha-factor arrest to degrade bem1-AID. They were then washed and released into the cell cycle for 70 minutes in methionine-depleted medium. Finally, cells were mounted on a 0.5 % agar gel containing auxin (0.5 mM) and methionine (0.08 g.l^−1^), sealed with halo-carbon oil and live-cell imaging was performed directly.

Live-cell imaging was carried out in a temperature-controlled room (22-25 °C) using a Ti-DH inverted microscope (Nikon) equipped with a ×100 oil Plan-Apochromat objective lens (N/A 1.45), a spinning disk (CSU-W1-T1, Yokogawa) and a sCMOS camera (Roper Scientific). The system was controlled by Metamorph software (Molecular Device). Depending on the number of acquisitions, total acquisition time and cell size, between 10 and 15 z-stacks with an interval of 0.3 or 0.5 µm were acquired.

Where necessary, cells were fixed with 3.7% formaldehyde for 20 min. After washing in PBS three times, budding was quantified (Fig. 5g and Supplementary Figs. 2b, 6b, 10c). To measure the percentage of multinucleate cells (Figs. 3c and 4f), cells were washed in PBS and incubated for one h with 10 µL of DAPI solution (Invitrogen). To visualize actin filaments, fixed cells were labeled with Alexa 546–phalloidin before imaging in PBS (Fig. 3f). In Fig. 1d, e, and Supplementary Fig. 5h and Supplementary Fig. 13, cells were also fixed with 3.7 % formaldehyde for 20 min and washed in PBS three times.

### Image analysis

Fiji (National Institutes of Health) was used for image analysis and fluorescence quantification. The background level of the camera was subtracted using a rolling ball radius of 50 pixels before quantification. For qualitative analyses, the timing of cell cycle events in mutant strains were compared with control cells. To better visualize these events, the images shown in Figs. 1a, d, h,2b,4g,5a, c and Supplementary Figs. 1d, 3a, 3e, 5e, 12a, 12b, 13a, 13b were median filtered (filter size 1 × 1), whereas quantitative analysis was performed on raw data.

Sec3-GFP polarization was defined when the signal accumulated at the plasma membrane for at least 10 minutes (Figs. 1b, 2b, c, e and Supplementary Figs. 1b, 5a, 5b, 5c). Cell separation and bud emergence were defined from DIC images. Whi5 complete exit from the nucleus was defined when no co-localization with nucleoplasmic Pus1 was observed (Fig. 1e and Supplementary Fig. 1e). The duration of Clb2 and Clb5 expression was defined qualitatively when a signal stronger than background was observed (Fig. 1 and Supplementary Fig. 2). Myo1 polarization was defined when the signal accumulated at the plasma membrane (Supplementary Figs. 3a–c). SPB separation was defined when the 2 SPBs were resolved, and anaphase onset was defined when the distance between the two SPBs doubled in one frame (Figs. 2, 4 and Supplementary Figs. 3, 5). Multinucleate cells were counted when 2 resolved nuclei were observed in the same cell compartment (mother cell or bud), by observing Pus1 signal (Fig. 1i and Supplementary Fig. 5h) or after DAPI treatment (Figs. 3c and 4f). Multinucleate cells in Fig. 2d and e were counted when anaphase was observed in cells that remained unbudded.

In Fig. 5b, Cln2 fluorescence intensity was measured in the whole cell on sum-intensity projected z-stacks in each time frame, where fluorescence intensity values were extracted after background subtraction and Pus1 levels, which are not cell cycle regulated, were used to normalize for bleaching. Cln2 fluorescence intensity was measured in sagittal sections through the cell in each time frame in Fig. 5d–f, where fluorescence intensity values were extracted after background subtraction and Pus1 levels were used to normalize for bleaching as well as delineating nuclear and cytoplasmic signals. A similar strategy was used in Supplementary Figs. 11, 12f-g and 13c-e, where fluorescence intensity values were extracted after background subtraction, and nuclear and cytoplasmic signals were measured using the Pus1 signal to delineate the nucleoplasm. Adobe Illustrator was used to generate schematic images of cells and experimental design.

### Flow-cytometric analysis of DNA contents

For DNA quantification by flow cytometry, 10^7^ cells were collected and fixed with 1 ml of 70 % ethanol for 30 min at room temperature. After one wash with 50 mM Tris-HCl pH 7.5, cells were resuspended in 0.5 ml of the same buffer containing 0.05 ml of a preboiled 10 mg.ml^−1^ RNAse solution and incubated overnight at 37 °C. Cells were then resuspended in 0.5 ml of 5 mg.ml^−1^ pepsin freshly diluted in 55 mM HCl. After 30 minutes at 37 °C, cells were washed with 0.5 ml of FACS buffer (200 mM Tris-HCl pH 7.5, 211 mM NaCl, 78 mM MgCl2) and diluted (1:10) in the same buffer containing 50 μg.ml^−1^ propidium iodide. After brief sonication, DNA content was determined using a BD Accuri C6 flow cytometer and analyzed using FlowJo software.

### Quantification of mRNA levels by RT-qPCR

To quantify *CLN2* mRNA levels by RT-qPCR, 10^7^ cells were collected during cell cycle time-courses and washed in 1.5 ml cold RNAse-free water. After centrifugation (1 min, 16000 g), water was removed, glass beads were added, and cells were flash frozen in liquid nitrogen. RNA was extracted using TRI Reagent. Samples were vigorously agitated after the addition of 450 μl TRI Reagent. Beads were then removed, and samples were incubated at room temperature for 5 minutes after adding another 550 μl TRI Reagent. Chloroform (200 µl) was added 5 minutes before centrifugation (15 min, 12000 g, 4 °C) and the supernatant was transferred to a new RNase-free tube to which 1 volume of isopropanol was added. After incubation for 15 minutes at room temperature, the samples were centrifuged (10 min, 12000 g, 4 °C) then RNA samples were washed with 1 ml 75 % ethanol. RNA was finally resuspended in 20 μl RNAse-free water.

RNA samples (2.4 µg) were treated with DNase for 25 minutes at 37 °C prior to the reverse-transcription reaction, which was carried out using a LunaScript RT kit (2 minutes at 25 °C, 10 minutes at 55 °C, 1 minute at 95 °C). cDNA samples were diluted in RNAse-free water (1:10), and SYBR green was used for quantification during the qPCRs. Samples were analyzed in 384-well plates in parallel (*CLN2* and *ACT1* mRNA levels). *ACT1* mRNA levels were used as controls for normalization.

### Statistical analysis

All plots and statistical analyses were performed with Prism software. Values from independent experiments are displayed as mean ± SD and the statistical tests used are indicated in the figure legends. Points representing individual values are also displayed. Sample numbers (*n* values), the number of independent experiments (*N* values) and plots where all biological replicates were combined in one graph are mentioned in figure legends. Exact sample numbers (*n* values) for Figs. 1e, 3c, 4f, 5g and Supplementary Figs. 5h, 13c, 13d, 13e are indicated in Source data. Exact *p*-values, as well as *F* values and degrees of freedom (for two-way ANOVAs) and *t*-values and degrees of freedom (for *t*-tests) are indicated in Source data. All the statistical tests used were unpaired, two-tailed. No statistical method was used to predetermine sample size. No data were excluded from the analyses. The experiments were not randomized. The investigators were not blinded to allocation during experiments, however strain numbers rather than genotypes were used to identify samples, providing semi-blinding. Source data are provided as a Source Data file.

In Supplementary Fig. 2b, the curves were smoothed using LOWESS analysis (medium). In Figs. 1a–b, 2c and Supplementary Figs. 3b, 11, 13, all individual values are also represented by points. After testing for the normal distribution of data, *t*-tests with Welch correction or Mann-Whitney tests were performed (*p < 0.05, **p < 0.01, ***p < 0.001, ****p < 0.0001). In Fig. 1i, the measurement of the percentage of multinucleate cells was repeated 3 times (shown as points) using the Pus1-GFP marker and all the cells were grouped after ensuring that these experimental repeats were not significantly different. Half-lives of Cln2 were calculated after nonlinear regression fitting (one phase decay or sigmoidal (4PL)), and the quality of data fitting with confidence intervals of 95 % are displayed in Supplementary Figs. 7 and 9.

### Cln2 purification and mass spectrometry

3xHA-Cln2-Cdc28^Cdk1^ was purified from a yeast strain expressing the construct from the *GAL1* promoter integrated at the *CLN2* locus. The active complex was purified in extract buffer (50 mM HEPES pH 7.6, 100 mM β-glycerophosphate, 50 mM NaF, 1 mM EGTA, 1 mM MgCl_2_, 5 % glycerol, 1 mM PMSF and 0.2 % Tween-20) using rabbit polyclonal anti-HA antibody, gently eluted using HA-dipeptide (0.5 mg ml^−1^) and stored with BSA for stability. Kinase assays were prepared in kinase assay buffer (50 mM HEPES pH 7.6, 2 mM MgCl_2_, 0.05% Tween-20, 10% glycerol and 1 mM DTT) in the presence or absence of 1 mM ATP^11^. Kinase assays containing ± 0.5 µg Cln2, as estimated from Coomassie blue stain, were incubated at 30 °C for 30 minutes. Protein was precipitated by adding trichloroacetic acid to 10% then analyzed by SDS-PAGE. Coomassie-stained SDS-PAGE gel slices containing Cln2 were excised, digested with trypsin and resuspended in 2.5% acetonitrile and 5% formic acid then analyzed by liquid chromatography tandem mass spectrometry (LC-MS/MS) using an LTQ-FThybrid linear ion trap-Fourier transform ion cyclotron resonance (FTCR) mass spectrometer (Thermo-Electron, San Jose, CA)^66^. Database searches were then performed against the *S. cerevisiae* Cln2 protein using the SEQUEST search algorithm. Database search parameters included a 1.1 Da mass tolerance, no enzyme specificity and amino acid modifications as follows: 15.9949 Da on methionine, 79.9663 Da on serine, threonine and tyrosine and 71.0371 Da on cysteine. Identified residues were subsequently filtered to be tryptic and fall within a 20 PPM window of the predicted mass. Validation was aided by the presence or absence of fragment ions characteristic of the neutral loss of phosphoric acid and by the employment of an in-house software package^67^. While the kinase assays were performed 4 times with the same results, mass spectrometry analysis of Cln2 phosphorylation sites was performed once.

### Reporting summary

Further information on research design is available in the Nature Portfolio Reporting Summary linked to this article.

## Supplementary information

Supplementary Information

Description of Additional Supplementary Files

Supplementary Movie 1

Supplementary Movie 2

Supplementary Movie 3

Supplementary Movie 4

Supplementary Movie 5

Supplementary Movie 6

Supplementary Movie 7

Supplementary Movie 8

Supplementary Movie 9

Supplementary Movie 10

Supplementary Movie 11

Supplementary Movie 12

Supplementary Movie 13

Supplementary Movie 14

Reporting Summary

Transparent Peer Review file

## Source data

Source data

## Data Availability

The authors declare that all data supporting the findings of this study are available within the paper and its supplementary information files. Source Data are provided with this paper. Plasmids and their sequences are available upon request. Raw mass spectrometry data is no longer available due to elapsed time since the experiments were performed, but identified residues are provided in Supplementary Information. Source data are provided with this paper.

## References

[1] Hartwell, L. H., Culotti, J., Pringle, J. R. & Reid, B. J. Genetic control of cell division cycle in yeast. *Science* **183**, 46–51 (1974). 4587263 10.1126/science.183.4120.46

[2] Costanzo, M. et al. CDK activity antagonizes Whi5, an inhibitor of G1/S transcription in yeast. *Cell* **117**, 899–913 (2004). 15210111 10.1016/j.cell.2004.05.024

[3] de Bruin, R. A., McDonald, W. H., Kalashnikova, T. I., Yates, J. 3rd & Wittenberg, C. Cln3 activates G1-specific transcription via phosphorylation of the SBF bound repressor Whi5. *Cell* **117**, 887–898 (2004). 15210110 10.1016/j.cell.2004.05.025

[4] Skotheim, J. M., Di Talia, S., Siggia, E. D. & Cross, F. R. Positive feedback of G1 cyclins ensures coherent cell cycle entry. *Nature* **454**, 291–296 (2008). 18633409 10.1038/nature07118 PMC2606905

[5] Charvin, G., Oikonomou, C., Siggia, E. D. & Cross, F. R. Origin of irreversibility of cell cycle start in budding yeast. *PLoS Biol.* **8**, e1000284 (2010). 20087409 10.1371/journal.pbio.1000284 PMC2797597

[6] Xiao, J., Turner, J. J., Kõivomägi, M. & Skotheim, J. M. Whi5 hypo- and hyper-phosphorylation dynamics control cell-cycle entry and progression. *Curr. Biol. CB* **34**, 2434–2447.e5 (2024). 38749424 10.1016/j.cub.2024.04.052 PMC11247822

[7] Moffat, J. & Andrews, B. Late-G1 cyclin-CDK activity is essential for control of cell morphogenesis in budding yeast. *Nat. Cell Biol.* **6**, 59–66 (2004). 14688790 10.1038/ncb1078

[8] Butty, A.-C., Pryciak, P. M., Huang, L. S., Herskowitz, I. & Peter, M. The role of Far1p in linking the heterotrimeric G protein to polarity establishment proteins during yeast mating. *Science* **282**, 1511–1516 (1998). 9822386 10.1126/science.282.5393.1511

[9] Nern, A. & Arkowitz, R. A. Nucleocytoplasmic shuttling of the Cdc42p exchange factor Cdc24p. *J. Cell Biol.* **148**, 1115–1122 (2000). 10725324 10.1083/jcb.148.6.1115 PMC2174313

[10] Knaus, M. et al. Phosphorylation of Bem2p and Bem3p may contribute to local activation of Cdc42p at bud emergence. *EMBO J.* **26**, 4501–4513 (2007). 17914457 10.1038/sj.emboj.7601873 PMC2063487

[11] McCusker, D. et al. Cdk1 coordinates cell-surface growth with the cell cycle. *Nat. Cell Biol.* **9**, 506–515 (2007). 17417630 10.1038/ncb1568

[12] Sopko, R., Huang, D., Smith, J. C., Figeys, D. & Andrews, B. J. Activation of the Cdc42p GTPase by cyclin-dependent protein kinases in budding yeast. *Embo J.* **26**, 4487–4500 (2007). 17853895 10.1038/sj.emboj.7601847 PMC2063489

[13] Zheng, X. D., Lee, R. T., Wang, Y. M., Lin, Q. S. & Wang, Y. Phosphorylation of Rga2, a Cdc42 GAP, by CDK/Hgc1 is crucial for Candida albicans hyphal growth. *Embo J.* **26**, 3760–3769 (2007). 17673907 10.1038/sj.emboj.7601814 PMC1952229

[14] Bhaduri, S. et al. A docking interface in the cyclin Cln2 promotes multi-site phosphorylation of substrates and timely cell-cycle entry. *Curr. Biol. CB*. **25**, 316–325 (2015). 25619768 10.1016/j.cub.2014.11.069 PMC4318751

[15] Kõivomägi, M. et al. Dynamics of Cdk1 substrate specificity during the cell cycle. *Mol. Cell* **42**, 610–623 (2011). 21658602 10.1016/j.molcel.2011.05.016 PMC3115021

[16] Pirincci Ercan, D. et al. Budding yeast relies on G1 cyclin specificity to couple cell cycle progression with morphogenetic development. *Sci. Adv.* **7**, eabg0007 (2021). 34088668 10.1126/sciadv.abg0007 PMC8177710

[17] Finegold, A. A. et al. Protein geranylgeranyl transferase of Saccharomyces cerevisiae is specific for Cys-Xaa-Xaa-Leu motif proteins and requires the CDC43 gene product but not the DPR1 gene product. *Proc. Natl. Acad. Sci. USA* **88**, 4448–4452 (1991). 2034682 10.1073/pnas.88.10.4448 PMC51677

[18] Kozminski, K. G., Chen, A. J., Rodal, A. A. & Drubin, D. G. Functions and functional domains of the GTPase Cdc42p. *Mol. Biol. Cell* **11**, 339–354 (2000). 10637312 10.1091/mbc.11.1.339 PMC14778

[19] Butty, A.-C. A positive feedback loop stabilizes the guanine-nucleotide exchange factor Cdc24 at sites of polarization. *EMBO J.* **21**, 1565–1576 (2002). 11927541 10.1093/emboj/21.7.1565 PMC125953

[20] Simpson, D., Johnson, D. I. & Toenjes, K. A. Separate membrane targeting and anchoring domains function in the localization of the S. cerevisiae Cdc24p guanine nucleotide exchange factor. *Curr. Genet.* **45**, 257–264 (2004). 14872283 10.1007/s00294-004-0485-9

[21] Das, S. et al. Single-molecule tracking of small GTPase Rac1 uncovers spatial regulation of membrane translocation and mechanism for polarized signaling. *Proc. Natl. Acad. Sci.* **112**, E267–E276 (2015). 25561548 10.1073/pnas.1409667112 PMC4311845

[22] Platre, M. P. et al. Developmental control of plant Rho GTPase nano-organization by the lipid phosphatidylserine. *Science* **364**, 57–62 (2019). 30948546 10.1126/science.aav9959

[23] Remorino, A. et al. Gradients of Rac1 Nanoclusters Support Spatial Patterns of Rac1 Signaling. *Cell Rep.* **21**, 1922–1935 (2017). 29141223 10.1016/j.celrep.2017.10.069

[24] Sartorel, E. et al. Phosphatidylserine and GTPase activation control Cdc42 nanoclustering to counter dissipative diffusion. *Mol. Biol. Cell* **29**, 1299–1310 (2018). 29668348 10.1091/mbc.E18-01-0051 PMC5994902

[25] Tian, T. et al. Plasma membrane nanoswitches generate high-fidelity Ras signal transduction. *Nat. Cell Biol.* **9**, 905–914 (2007). 17618274 10.1038/ncb1615

[26] Das, A. et al. Flippase-mediated phospholipid asymmetry promotes fast Cdc42 recycling in dynamic maintenance of cell polarity. *Nat. Cell Biol.* **14**, 304–310 (2012). 22344035 10.1038/ncb2444 PMC3534761

[27] Fairn, G. D., Hermansson, M., Somerharju, P. & Grinstein, S. Phosphatidylserine is polarized and required for proper Cdc42 localization and for development of cell polarity. *Nat. Cell Biol.* **13**, 1424–1430 (2011). 21964439 10.1038/ncb2351

[28] Haupt, A. & Minc, N. Gradients of phosphatidylserine contribute to plasma membrane charge localization and cell polarity in fission yeast. *Mol. Biol. Cell* **28**, 210–220 (2017). 27852900 10.1091/mbc.E16-06-0353 PMC5221626

[29] Zhou, Y., Prakash, P. S., Liang, H., Gorfe, A. A. & Hancock, J. F. The KRAS and other prenylated polybasic domain membrane anchors recognize phosphatidylserine acyl chain structure. *Proc. Natl. Acad. Sci. UA* **118**, e2014605118 (2021). 10.1073/pnas.2014605118 PMC8017956 33526670

[30] Meca, J. et al. Avidity-driven polarity establishment via multivalent lipid-GTPase module interactions. *EMBO J.* **38**, e99652 (2019). 30559330 10.15252/embj.201899652 PMC6356062

[31] Smith, S. E. et al. Independence of symmetry breaking on Bem1-mediated autocatalytic activation of Cdc42. *J. Cell Biol.* **202**, 1091–1106 (2013). 24062340 10.1083/jcb.201304180 PMC3787378

[32] Rapali, P. et al. Scaffold-mediated gating of Cdc42 signalling flux. *Elife* **6**, e25257 (2017). 28304276 10.7554/eLife.25257 PMC5386590

[33] Witte, K., Strickland, D. & Glotzer, M. Cell cycle entry triggers a switch between two modes of Cdc42 activation during yeast polarization. *Elife* **6**, e26722 (2017). 28682236 10.7554/eLife.26722 PMC5536948

[34] Lew, D. J. & Reed, S. I. A cell cycle checkpoint monitors cell morphogenesis in budding yeast. *J. Cell Biol.* **129**, 739–749 (1995). 7730408 10.1083/jcb.129.3.739 PMC2120431

[35] Harvey, S. L. & Kellogg, D. R. Conservation of mechanisms controlling entry into mitosis: budding yeast wee1 delays entry into mitosis and is required for cell size control. *Curr. Biol. CB* **13**, 264–275 (2003). 12593792 10.1016/s0960-9822(03)00049-6

[36] Booher, R. N., Deshaies, R. J. & Kirschner, M. W. Properties of Saccharomyces cerevisiae wee1 and its differential regulation of p34CDC28 in response to G1 and G2 cyclins. *Embo J.* **12**, 3417–3426 (1993). 8253069 10.1002/j.1460-2075.1993.tb06016.x PMC413617

[37] McMillan, J. N., Sia, R. A. L. & Lew, D. J. A Morphogenesis Checkpoint Monitors the Actin Cytoskeleton in Yeast. *J. Cell Biol.* **142**, 1487–1499 (1998). 9744879 10.1083/jcb.142.6.1487 PMC2141759

[38] McNulty, J. J. & Lew, D. J. Swe1p responds to cytoskeletal perturbation, not bud size, in S. cerevisiae. *Curr. Biol*. *CB* **15**, 2190–2198 (2005). 10.1016/j.cub.2005.11.039 16360682

[39] Barral, Y., Parra, M., Bidlingmaier, S. & Snyder, M. Nim1-related kinases coordinate cell cycle progression with the organization of the peripheral cytoskeleton in yeast. *Genes Dev.* **13**, 176–187 (1999). 9925642 10.1101/gad.13.2.176 PMC316392

[40] Carroll, C. W., Altman, R., Schieltz, D., Yates, J. R. & Kellogg, D. The septins are required for the mitosis-specific activation of the Gin4 kinase. *J. Cell Biol.* **143**, 709–717 (1998). 9813092 10.1083/jcb.143.3.709 PMC2148151

[41] Chiroli, E., Rossio, V., Lucchini, G. & Piatti, S. The budding yeast PP2ACdc55 protein phosphatase prevents the onset of anaphase in response to morphogenetic defects. *J. Cell Biol.* **177**, 599–611 (2007). 17502422 10.1083/jcb.200609088 PMC2064206

[42] Lianga, N. et al. A Wee1 checkpoint inhibits anaphase onset. *J. Cell Biol.* **201**, 843–862 (2013). 23751495 10.1083/jcb.201212038 PMC3678162

[43] Sreenivasan, A. & Kellogg, D. The Elm1 Kinase Functions in a Mitotic Signaling Network in Budding Yeast. *Mol. Cell. Biol.* **19**, 7983–7994 (1999). 10567524 10.1128/mcb.19.12.7983 PMC84883

[44] Zhang, X. et al. Membrane association and functional regulation of Sec3 by phospholipids and Cdc42. *J. Cell Biol.* **180**, 145–158 (2008). 18195105 10.1083/jcb.200704128 PMC2213614

[45] Stahelin, R. V., Karathanassis, D., Murray, D., Williams, R. L. & Cho, W. Structural and Membrane Binding Analysis of the Phox Homology Domain of Bem1p. *J. Biol. Chem.* **282**, 25737–25747 (2007). 17581820 10.1074/jbc.M702861200

[46] Lew, D. J. & Reed, S. I. Morphogenesis in the yeast cell cycle: regulation by Cdc28 and cyclins. *J. Cell Biol.* **120**, 1305–1320 (1993). 8449978 10.1083/jcb.120.6.1305 PMC2119756

[47] Kõivomägi, M. et al. Cascades of multisite phosphorylation control Sic1 destruction at the onset of S phase. *Nature* **480**, 128–131 (2011). 21993622 10.1038/nature10560 PMC3228899

[48] Schwob, E., Bohm, T., Mendenhall, M. & Nasmyth, K. The B-type cyclin kinase inhibitor p40sic1 controls the G1 to S transition in S. cerevisiae. *Cell* **79**, 233–244 (1994). 7954792 10.1016/0092-8674(94)90193-7

[49] Harvey, S. L., Charlet, A., Haas, W., Gygi, S. P. & Kellogg, D. R. Cdk1-Dependent Regulation of the Mitotic Inhibitor Wee1. *Cell* **122**, 407–420 (2005). 16096060 10.1016/j.cell.2005.05.029

[50] Mortensen, E. M., McDonald, H., Yates, J. & Kellogg, D. R. Cell Cycle-dependent Assembly of a Gin4-Septin Complex. *Mol. Biol. Cell* **13**, 2091–2105 (2002). 12058072 10.1091/mbc.01-10-0500 PMC117627

[51] Tjandra, H., Compton, J. & Kellogg, D. Control of mitotic events by the Cdc42 GTPase, the Clb2 cyclin and a member of the PAK kinase family. *Curr. Biol.* **8**, 991–1000 (1998). 9740799 10.1016/s0960-9822(07)00419-8

[52] Morawska, M. & Ulrich, H. D. An expanded tool kit for the auxin-inducible degron system in budding yeast. *Yeast* **30**, 341–351 (2013). 23836714 10.1002/yea.2967 PMC4171812

[53] Nishimura, K., Fukagawa, T., Takisawa, H., Kakimoto, T. & Kanemaki, M. An auxin-based degron system for the rapid depletion of proteins in nonplant cells. *Nat. Methods* **6**, 917–922 (2009). 19915560 10.1038/nmeth.1401

[54] Ayscough, K. R. et al. High rates of actin filament turnover in budding yeast and roles for actin in establishment and maintenance of cell polarity revealed using the actin inhibitor latrunculin-A. *J. Cell Biol.* **137**, 399–416 (1997). 9128251 10.1083/jcb.137.2.399 PMC2139767

[55] Amon, A., Tyers, M., Futcher, B. & Nasmyth, K. Mechanisms that help the yeast cell cycle clock tick: G2 cyclins transcriptionally activate G2 cyclins and repress G1 cyclins. *Cell* **74**, 993–1007 (1993). 8402888 10.1016/0092-8674(93)90722-3

[56] TerBush, D. R. & Novick, P. Sec6, Sec8, and Sec15 are components of a multisubunit complex which localizes to small bud tips in Saccharomyces cerevisiae. *J. Cell Biol.* **130**, 299–312 (1995). 7615633 10.1083/jcb.130.2.299 PMC2199927

[57] Sagot, I., Klee, S. K. & Pellman, D. Yeast formins regulate cell polarity by controlling the assembly of actin cables. *Nat. Cell Biol.* **4**, 42–50 (2002). 11740491 10.1038/ncb719

[58] Patton, E. E. et al. Cdc53 is a scaffold protein for multiple Cdc34/Skp1/F-box protein complexes that regulate cell division and methionine biosynthesis in yeast. *Genes Dev.* **12**, 692–705 (1998). 9499404 10.1101/gad.12.5.692 PMC316590

[59] Lanker, S., Valdivieso, M. H. & Wittenberg, C. Rapid Degradation of the G1 Cyclin Cln2 Induced by CDK-Dependent Phosphorylation. *Science* **271**, 1597–1601 (1996). 8599119 10.1126/science.271.5255.1597

[60] Breitkreutz, A. et al. A global protein kinase and phosphatase interaction network in yeast. *Science* **328**, 1043–1046 (2010). 20489023 10.1126/science.1176495 PMC3983991

[61] Lanz, M. C. et al. In-depth and 3-dimensional exploration of the budding yeast phosphoproteome. *EMBO Rep.* **22**, e51121 (2021). 33491328 10.15252/embr.202051121 PMC7857435

[62] Edgington, N. P. & Futcher, B. Relationship between the function and the location of G1 cyclins in S. cerevisiae. *J. Cell Sci.* **114**, 4599–4611 (2001). 11792824 10.1242/jcs.114.24.4599

[63] Miller, M. E. & Cross, F. R. Distinct subcellular localization patterns contribute to functional specificity of the Cln2 and Cln3 cyclins of Saccharomyces cerevisiae. *Mol. Cell Biol.* **20**, 542–555 (2000). 10611233 10.1128/mcb.20.2.542-555.2000 PMC85127

[64] Knudsen, E. S., Witkiewicz, A. K. & Rubin, S. M. Cancer takes many paths through G1/S. *Trends Cell Biol.* **34**, 636–645 (2024). 37953123 10.1016/j.tcb.2023.10.007 PMC11082069

[65] Laan, L., Koschwanez, J. H. & Murray, A. W. Evolutionary adaptation after crippling cell polarization follows reproducible trajectories. *eLife* **4**, e09638 (2015). 26426479 10.7554/eLife.09638 PMC4630673

[66] Mimura, S., Seki, T., Tanaka, S. & Diffley, J. F. Phosphorylation-dependent binding of mitotic cyclins to Cdc6 contributes to DNA replication control. *Nature* **431**, 1118–1123 (2004). 15496876 10.1038/nature03024

[67] Beausoleil, S. A., Villen, J., Gerber, S. A., Rush, J. & Gygi, S. P. A probability-based approach for high-throughput protein phosphorylation analysis and site localization. *Nat. Biotechnol.* **24**, 1285–1292 (2006). 16964243 10.1038/nbt1240

