# Supplementary material for: A cyclin-polarity feedback network ensures healthy cell proliferation
Source: Nat Commun. 2026 Jun 19;17:7731. doi: 10.1038/s41467-026-74596-7 (PMC13434654; doi:10.1038/s41467-026-74596-7)
Supplement: Supplementary file 1 [file 41467_2026_74596_MOESM17_ESM.pdf]

nature portfolio

Corresponding author(s): Derek McCusker

Last updated by author(s): May 20, 2026

Reporting Summary

Nature Portfolio wishes to improve the reproducibility of the work that we publish. This form provides structure for consistency and transparency in reporting. For further information on Nature Portfolio policies, see our Editorial Policies and the Editorial Policy Checklist.

Statistics

For all statistical analyses, confirm that the following items are present in the figure legend, table legend, main text, or Methods section.

n/a	Confirmed	
☐	☒	The exact sample size (n) for each experimental group/condition, given as a discrete number and unit of measurement
☐	☒	A statement on whether measurements were taken from distinct samples or whether the same sample was measured repeatedly
☐	☒	The statistical test(s) used AND whether they are one- or two-sided<br>*Only common tests should be described solely by name; describe more complex techniques in the Methods section.*
☒	☐	A description of all covariates tested
☒	☐	A description of any assumptions or corrections, such as tests of normality and adjustment for multiple comparisons
☐	☒	A full description of the statistical parameters including central tendency (e.g. means) or other basic estimates (e.g. regression coefficient) AND variation (e.g. standard deviation) or associated estimates of uncertainty (e.g. confidence intervals)
☐	☒	For null hypothesis testing, the test statistic (e.g. F, t, r) with confidence intervals, effect sizes, degrees of freedom and P value noted<br>*Give P values as exact values whenever suitable.*
☒	☐	For Bayesian analysis, information on the choice of priors and Markov chain Monte Carlo settings
☒	☐	For hierarchical and complex designs, identification of the appropriate level for tests and full reporting of outcomes
☒	☐	Estimates of effect sizes (e.g. Cohen's d, Pearson's r), indicating how they were calculated

Our web collection on statistics for biologists contains articles on many of the points above.

Software and code

Policy information about availability of computer code

Data collection	For microscopy: Metamorph 6<br>For flow cytometry: Accuri C6
Data analysis	Fiji, Prism 8, FlowJo 10, Adobe Illustrator CS5, Adobe Photoshop CS5, Adobe Acrobat Pro.

For manuscripts utilizing custom algorithms or software that are central to the research but not yet described in published literature, software must be made available to editors and reviewers. We strongly encourage code deposition in a community repository (e.g. GitHub). See the Nature Portfolio guidelines for submitting code & software for further information.

Data

Policy information about availability of data

All manuscripts must include a data availability statement. This statement should provide the following information, where applicable:

- Accession codes, unique identifiers, or web links for publicly available datasets
- A description of any restrictions on data availability
- For clinical datasets or third party data, please ensure that the statement adheres to our policy

The authors declare that all data supporting the findings of this study are available within the paper and its supplementary information files. Source Data are provided with this paper.

Research involving human participants, their data, or biological material

Policy information about studies with human participants or human data. See also policy information about sex, gender (identity/presentation), and sexual orientation and race, ethnicity and racism.

Reporting on sex and gender	N/A
Reporting on race, ethnicity, or other socially relevant groupings	N/A
Population characteristics	N/A
Recruitment	N/A
Ethics oversight	N/A

Note that full information on the approval of the study protocol must also be provided in the manuscript.

Field-specific reporting

Please select the one below that is the best fit for your research. If you are not sure, read the appropriate sections before making your selection.

[x] Life sciences [ ] Behavioural & social sciences [ ] Ecological, evolutionary & environmental sciences

For a reference copy of the document with all sections, see nature.com/documents/nr-reporting-summary-flat.pdf

Life sciences study design

All studies must disclose on these points even when the disclosure is negative.

Sample size	Indicated in the figure legends. No statistical method was used to predetermine the sample size. Sample sizes were chosen to enable rigorous testing of hypotheses. All experiments in the paper were repeated to check for reproducibility of results and to apply statistical tests, as described in the Figure legends.
Data exclusions	No data were excluded from the analyses.
Replication	All the experiments were reproduced at least 3 times on different days. The exact number of biological replicates is indicated in the figure legends.
Randomization	The experiments were not randomized.
Blinding	The investigators were not blinded to sample allocation during experiments; however samples were only labelled with strain numbers rather than any specific genotype information.

Reporting for specific materials, systems and methods

We require information from authors about some types of materials, experimental systems and methods used in many studies. Here, indicate whether each material, system or method listed is relevant to your study. If you are not sure if a list item applies to your research, read the appropriate section before selecting a response.

Materials & experimental systems

n/a	Involved in the study	
[ ]	[x]	Antibodies
[x]	[ ]	Eukaryotic cell lines
[x]	[ ]	Palaeontology and archaeology
[ ]	[x]	Animals and other organisms
[x]	[ ]	Clinical data
[x]	[ ]	Dual use research of concern
[x]	[ ]	Plants

Methods

n/a	Involved in the study	
[x]	[ ]	ChIP-seq
[ ]	[x]	Flow cytometry
[x]	[ ]	MRI-based neuroimaging

Antibodies

Antibodies used	- anti-HA (12CA5) (gift from Dr R. Arkowitz, iBV, Nice). Dilution 1/1000<br>- anti-TAP (IgG peroxidase, ChromPure Jackson ImmunoResearch. 011-030-003. Lot 105131). Dilution 1/1500<br>- anti-FLAG (M2, Sigma. F3165. Lot SLCP 4941). Dilution 1/500

	- anti-c-MYC (9E10) (gift from Prof. B Lillemeier, Univ. Freiburg). Dilution 1/1500<br>- anti-V5 (Clone SV5-Pk3. Bio-Rad. MCA2893GA). Dilution 1/1500<br>- anti-Clb2 (Rabbit polyclonal. Gift from Prof. D. Kellogg, UCSC. See PMID: 9740799). Dilution 1/4000<br>- anti-Swe1 (Rabbit polyclonal. Gift from Prof. D. Kellogg, UCSC. See PMID: 12917352). Dilution 1/1000<br>- anti-Gin4 (Rabbit polyclonal. Gift from Doug Kellogg. See PMID: 12058072). Dilution 1/1000<br>- anti-Ade13 (Rabbit polyclonal. Gift from Benoit Pinson. See PMID: 25947137). Dilution 1/10000<br>- anti-Rga2 (Rabbit polyclonal raised in lab. See PMID: 17417630). Dilution 1/1000<br>- anti-Y19P (phospho-cdc2 (Y15) Cell Signaling Technology. 4539L Lot 13). Dilution 1/1500
Validation	Antibodies were validated on yeast cell extracts from strains lacking the protein of interest and/or by the manifacturer as detailed on their website.

Animals and other research organisms

Policy information about studies involving animals; ARRIVE guidelines recommended for reporting animal research, and Sex and Gender in Research

Laboratory animals	Saccharomyces cerevisiae
Wild animals	N/A
Reporting on sex	N/A
Field-collected samples	The study did not involve samples collected from the field.
Ethics oversight	N/A

Note that full information on the approval of the study protocol must also be provided in the manuscript.

Plants

Seed stocks	N/A
Novel plant genotypes	N/A
Authentication	N/A

Flow Cytometry

Plots

Confirm that:

☐ The axis labels state the marker and fluorochrome used (e.g. CD4-FITC).

☒ The axis scales are clearly visible. Include numbers along axes only for bottom left plot of group (a 'group' is an analysis of identical markers).

☒ All plots are contour plots with outliers or pseudocolor plots.

☒ A numerical value for number of cells or percentage (with statistics) is provided.

Methodology

Sample preparation	Sample preparation is indicated in Methods.
Instrument	Accuri C6
Software	FlowJo
Cell population abundance	15000 cells/sample
Gating strategy	No gating

☐ Tick this box to confirm that a figure exemplifying the gating strategy is provided in the Supplementary Information.
