# Supplementary material for: A cyclin-polarity feedback network ensures healthy cell proliferation
Source: Nat Commun. 2026 Jun 19;17:7731. doi: 10.1038/s41467-026-74596-7 (PMC13434654; doi:10.1038/s41467-026-74596-7)
Supplement: Supplementary file 2 [file 41467_2026_74596_MOESM18_ESM.pdf]

nature portfolio

Peer Review File

A cyclin-polarity feedback network ensures healthy cell proliferation

Corresponding Author: Dr Derek McCusker

This file contains all reviewer reports in order by version, followed by all author rebuttals in order by version.

Version 0:

Reviewer comments:

Reviewer #1

(Remarks to the Author)
In budding yeast, the G1 cyclin-associated CDK is required for polarized bud formation, and defects in this process are monitored by the morphogenesis checkpoint to prevent mitosis. The manuscript by Peyran et al. first used bem1 cdc24lip anionic lipid binding mutants to induce defective polarity establishment. The results with bem1 cdc24lip mutants demonstrate that polarity defects stabilize G1 cyclin Cln2 as well as Swe1 kinase, which prevents mitotic progression by phosphorylating and inhibiting CDK1. In this study, bem1-AID cdc24lip mutants were also generated to further strengthen the connection between polarity and G1 cyclin, and between G1 cyclin and Swe1. A particular interesting observation is from the phospho-deficient and phospho-mimetic cln2 mutants, showing that Cln2 phosphorylation prevents its nuclear localization and degradation, while also sustaining CDK1 phosphorylation by Swe1. However, this manuscript lacks sufficient mechanistic details about the link between Cln2 phosphorylation and Swe1 stabilization. Moreover, 24 residues were mutated to generate cln2 phospho-mutants, leading to ambiguity. Results from specific cln2 phospho-mutants may further clarify the role of each phosphorylation event in the regulation of Swe1 stability and cell cycle progression. Therefore, major revision is required before this manuscript can be considered by Nature Communication.

Major points
1. The conclusion from Fig. 2 is that increased Cln2 phosphorylation and extended expression in bem1 cdc24lip cells is caused by defective polarized growth, but not an indirect consequence of Swe1 checkpoint activation. The evidence supporting this conclusion is relatively weak. To exclude the role of Swe1 in regulating Cln2, one possible experiment is to examine Cln2 protein level and modification in WT cells overexpressing Swe1.
2. G1 cyclin signaling contributes to Swe1 activity (Fig. 4). This figure shows persistent Cdc28 phosphorylation by Swe1 in bem1-AID cdc24lip mutants expressing Cln2-Asp, but cells expressing Cln2-Ala exhibit a slightly faster disappearance of Y19-P Cdc28 compared to control cells. Although these results indicate that Cln2 phosphorylation affects Swe1 activity, it is unclear if this effect is direct or indirect. Swe1 degradation requires its bud-neck localization. It might be worthwhile to examine polarity establishment in bem1-AID cdc24lip mutants expressing Cln2-Asp or Cln2-Ala, because one possibility is that expression of Cln2-Asp further exacerbates polarity establishment, which indirectly stabilizes Swe1.
As a control, the polarity establishment in WT cells (but not bem1 cdc24lip mutants) expressing Cln2-Asp or Cln2-Ala need to be examined. In addition, Swe1 phosphorylation and protein levels should be included in this figure, because this result will be informative for the connection between Cln2 phosphorylation and Swe1 phosphorylation/stability.
3. The cln2-Asp and cln2-Ala mutants. In these mutants, 24 amino acid residues are replaced with either Ala or Asp based on phosphorylation sites identified by MS, including two CDK sites. Previous results show that CDK1-dependent Cln2 phosphorylation regulates its localization and degradation. It would be informative to generate cln2 mutants with mutated CDK phosphorylation sites and further examine their localization and polarization phenotypes.
It seems that cln2 mutants lacking CDK phosphorylation sites are available. It is unclear if Swe1 activity has been examined in these mutants.
4. The logic for Fig. 3 is not easy to follow. It would be better to include a diagram showing the two models. Also, the text for this figure needs to be reworded with a clear rationale for the experimental design. A clear increase in Cln2 protein level and delayed Cln2 band-shift are noticed in Fig. 3A, and this should be taken into account when drawing conclusions.

Minor points:
-There is no page number.
- The manuscript would benefit from English editing, as the logical flow is sometimes difficult to follow.

-A better description of yeast strain bem1 cdc24lip is necessary in the main text. Also, the strain name bem1lip cdc24lip for this mutant seems more appropriate based on the description.
-Figure 1A: More information is needed to describe how to define cell separation as time 0.
-Figure 1E: Why is the faster nuclear release of Whi5 observed in bem1 cdc24lip cells? Is this phenotype dependent on Cln3-Cdc28 activation?
-It seems that bem1 cdc24lip cells exhibit a significant defect in polarity establishment, thus, slow cell growth is expected. It is unclear how these mutant cells are synchronized in G1 phase in Fig. 1f.
-A brief explanation of cdc28 Y19F is needed when it is mentioned for the first time (Fig. 2b, page xxx).
-Description for supplementary Fig. 2D could not be found in the main text.
- "Moreover, the phosphorylation that we observe in the bem1 cdc24lip mutant is associated with an increased window of Cln2 expression rather than its destabilization". My understanding is that destabilization of Cln2 is an important factor in controlling Cln2 expression window. This needs to be reworded.
-Figure 5. It will be interesting to examine the localization of Cln2 with mutated CDK phosphorylation sites. This will directly detect the role of Cln2 phosphorylation by CDK in its localization and degradation.

Reviewer #2

(Remarks to the Author)
This manuscript tackles a fundamental question in cell biology: how cells maintain cell cycle fidelity in the face of polarity disruption. The authors present compelling data showing that polarity defects lead to a loss of coordination between nuclear and cytoplasmic functions of G1 cyclin-CDK, and widespread disruptions to cell cycle timing. A particularly well-supported finding is the increased activity of Swe1, which delays cell cycle progression and may provide time for error correction. These aspects of the study are clearly demonstrated and provide a solid foundation for the manuscript. Building on this, the authors propose that regulation of the G1 cyclin Cln2 is central to this response, suggesting that Cln2 phosphorylation relays signals to Swe1 to delay the cell cycle, and contributes to error correction via enhanced phosphorylation of the GTPase regulators. However, these conclusions are less well supported. Much of the data is correlative and does not directly test the proposed model. To substantiate these claims, two major questions need to be addressed: (1) How is Cln2 expression prolonged in response to polarity disruption, and what are the relative contributions of transcriptional regulation versus phosphorylation? (2) Are the observed changes in Cln2 expression sufficient to activate Swe1 and delay the cell cycle? In summary, while the manuscript presents a promising and conceptually interesting model, additional mechanistic data are required to validate the proposed role of Cln2 in coordinating the polarity response.

Major concerns
1. With the exception of Fig. 1A–C, all experiments in the manuscript rely on alpha-factor arrest and release to investigate cell cycle differences in mutant strains. However, alpha-factor not only arrests cells in G1 but also induces polarization, resulting in shmoo formation. Consequently, cells polarize during the arrest phase, are then released, and subsequently undergo re-polarization—raising the question of whether this sequence could influence the observed results. Importantly, synchronization may not be essential for several of the experiments presented. As demonstrated in Fig. 1A, imaging asynchronous cells and analyzing only those that enter G1 during the experiment is a viable alternative. This caveat of alpha factor should be mentioned, and it would strengthen the manuscript to validate key findings in asynchronous cells. For example, if fluorescent Whi5 was included in the experiment shown in Fig. 1A, would the results match those in Fig. 1D–E? Similarly, do the findings in Fig. 2B–C hold when cells are not pre-synchronized with alpha-factor?

2. In both Fig. 3A and Fig. 4A-D, it's difficult to compare the different mutants directly due to how the experiments are presented. First, because the samples from different strains are run on separate gels, it's not possible to draw reliable conclusions about relative expression levels or phosphorylation states of the different proteins across mutants. While lane availability is understandably limited, including select time points (e.g., 15, 30, and 60 minutes) from each strain on the same gel would allow for direct comparison and better support the authors' claims about relative expression and phosphorylation.

3. Second, in Fig. 3A and 4A-D, the interpretation of the data would benefit from flow cytometry analysis of DNA content to assess synchrony in the release experiments across strains. Without this, it's unclear whether differences in marker expression reflect true biological differences or simply variation in cell cycle progression—for example, if a subset of cells in one strain is delayed in a particular phase, it could give the appearance of prolonged marker expression.

4. The increase in Cln2 half-life in the polarity mutant is quite modest compared to the well-characterized, stable 4T3S mutant. Given the significant disruption to cell cycle dynamics, it seems likely that the prolonged Cln2 expression observed is primarily due to altered transcription rather than increased protein stability alone. To test this, Cln2 mRNA levels should be measured in the experiment shown in Fig. 3A. If transcription is elevated or prolonged in the mutant, it could explain the increased protein levels. Conversely, if mRNA levels are comparable between mutant and wild-type cells, this would strongly support a role for Cln2 phosphorylation and/or stability in the polarity sensing mechanism.

5. The rationale for the Cln2 phosphomutants tested in this study is not clearly established, which makes it challenging to interpret the experimental results. The text suggests that the authors mutated all phosphorylation sites previously identified under various conditions from large-scale proteomic studies, rather than focusing on sites specifically phosphorylated during polarity disruption. Among these, two are known CDK sites that influence Cln2 degradation, but the kinases responsible for the remaining sites are either unknown or not discussed. To convincingly link polarity defects to changes in Cln2 phosphorylation and expression—an effect the authors propose as causative for the observed cell cycle delay—it would be important to first identify which Cln2 phosphosites are actually altered under polarity-disrupted conditions. Targeted mutation

of those specific sites would strengthen the mechanistic connection. Additionally, generating separate mutants for CDK versus non-CDK sites could provide insight into which kinases may regulate Cln2 in the context of polarity disruption.

6. The functional consequences of the cln2-Ala and cln2-Asp mutants remain unclear and warrant more thorough characterization. First, it is important to assess whether either mutant affects Cln2 protein stability. The Asp mutant appears to be expressed at higher levels and persists longer, which is unexpected given that phosphorylation typically targets Cln2 for degradation. One possible explanation is that phosphomimetic mutations often fail to recreate the structural interface required for recognition by SCF ubiquitin ligases, thereby impairing degradation. However, if this were the case, the Ala mutant—which prevents phosphorylation—might be expected to behave similarly, raising further questions. Second, the data in Figure 4 do not clearly demonstrate whether the mutations affect Cln2 localization and whether it is localization that confers the phenotype. The apparent cytoplasmic accumulation of the Asp mutant may simply reflect its elevated expression level rather than a true change in localization. If WT Cln2 or Cln2-Ala are relocalized to the cytoplasm do they phenocopy the Asp mutant?

7. Additional experiments are needed to establish a causal relationship between increased Cln2 levels or activity and Swe1 activation. Specifically, does overexpression of Cln2, or expression of a phosphomimetic mutant targeting relevant sites, lead to Swe1 activation in the absence of polarity disruption? Directly testing this would strengthen the proposed model and clarify whether Cln2 alone is sufficient to trigger this aspect of the checkpoint response.

Additional comments
8. The introduction states "The irreversibility of cell cycle commitment is thought to be mediated by a positive feedback loop where the G1-cyclin Cln3-Cdc28Cdk1 phosphorylates and partially inhibits Whi5...". More recent work shows that Cln3 doesn't directly phosphorylate Whi5 (Koivomagi et al, Science 2021; PMID: 34648313). This statement should be revised.

9. The text states "the resulting polarity defects also triggered a Swe1Wee1-dependent mitotic delay via inhibitory phosphorylation of Cdc28Cdk1 on Y19 (Supplementary Fig. 1i)". It is not clear what the purpose of Sup Fig 1i is, but it does not support this statement.

10. Many figures require more detail about the number of biological replicates performed (Figs. 1B, 1E, 1I, 2C, 3B, 4F, 4H, 5B, 5D-F). It should also be stated whether cells from multiple replicates have been pooled together to generate the plots.

11. Fig 2D-E and 4I should include error bars.

12. It is not clear whether any mass spectrometry analysis was performed in this study to map Cln2 phosphosites. There is a section in the methods describing a purification/mass spectrometry experiment, but no results from such an experiment are presented in the manuscript, and the text suggests that the phosphosite mutants that are tested were designed based on other published, large-scale datasets. If this experiment was performed, it should be included.

Version 1:

Reviewer comments:

Reviewer #1

(Remarks to the Author)
I believe all my concerns have been adequately addressed in the revised version. In my opinion, it is suitable for publication.
Yanchang

Reviewer #2

(Remarks to the Author)
The authors have done a good job of addressing my concerns in this revised manuscript. I now support publication.

Open Access This Peer Review File is licensed under a Creative Commons Attribution 4.0 International License, which permits use, sharing, adaptation, distribution and reproduction in any medium or format, as long as you give appropriate credit to the original author(s) and the source, provide a link to the Creative Commons license, and indicate if changes were made.
In cases where reviewers are anonymous, credit should be given to 'Anonymous Referee' and the source.
The images or other third party material in this Peer Review File are included in the article's Creative Commons license, unless indicated otherwise in a credit line to the material. If material is not included in the article's Creative Commons license and your intended use is not permitted by statutory regulation or exceeds the permitted use, you will need to obtain permission directly from the copyright holder.
To view a copy of this license, visit https://creativecommons.org/licenses/by/4.0/

We are very grateful to the reviewers for the time that they have spent reviewing the manuscript and for their constructive comments that have enabled us to improve it. Below, we provide a general overview of the additional experiments that we have now conducted before a point-by-point response to all of the comments raised during review. Changes to the manuscript text are in purple font.

- Using two independent approaches to inactivate the Swe1^{Wee1}-dependent checkpoint, we demonstrate that Cln2 is stabilized in response to polarity defects, indicating that the regulation of Cln2 expression in response to polarity defects is independent of Swe1 checkpoint activation.
- We analyzed cell cycle dynamics in otherwise WT cells expressing cln2-24A or cln2-24D. We also present analyses of Swe1^{Wee1} phosphorylation and stability in these mutants.
- Gal shut-off experiments are presented, indicating that the half-life of cln2-24A and cln2-24D proteins in WT cells are similar, in contrast to the *cln2-4T3S* mutant in which Cdc28^{Cdk1} consensus sites in Cln2 are mutated, resulting in its stabilization.
- Mutants of Cdc28^{Cdk1} consensus sites in cln2 (*cln2-4T3S*) were generated and the localization of this mutant and its phenotype in relation to polarity establishment were analyzed.
- A model has been provided to clarify the hypothesis being tested in Figure 3. - The text has also been edited and additional explanatory text has been added to clarify points that were raised during review. The nomenclature of the lipid binding mutant has also been changed to *bem1^{lip} cdc24^{lip}* throughout the manuscript.
- We have analyzed the contribution of transcription to increased G1 cyclin expression by RT-qPCR.
- Additional experiments are presented comparing the cell cycle dynamics of different fluorescent markers in WT and *bem1^{lip} cdc24^{lip}* mutants using asynchronous cells in order to verify that observed differences in cell cycle progression are not due to cell synchronization effects. These include comparison of Whi5 nuclear export kinetics, Myo1 polarization, Sec3 polarization, Clb2 expression, SPB separation, the nucleo/cytoplasmic ratio of Cln2 and the timing of budding.
- Cell cycle time-course experiments in which phosphorylation of Cln2 is compared between different mutants have been run on the same gels, facilitating direct comparison of electrophoretic mobility shifts by western blotting.
- FACS analysis of WT and *bem1^{lip} cdc24^{lip}* mutants are presented.
- We present analysis of Cln2-sfGFP nucleo-cytoplasmic dynamics in mutants where either Cdc28^{Cdk1} consensus sites (*cln2-4T3S*) or all sites except Cdc28^{Cdk1} sites (*cln2-22A*) were mutated to alanine.
- Analysis of cln2-24A-sfGFP dynamics and phenotype after adding a heterologous NES targeting sequence are presented.

Reviewer #1 (Remarks to the Author)

In budding yeast, the G1 cyclin-associated CDK is required for polarized bud formation, and defects in this process are monitored by the morphogenesis checkpoint to prevent mitosis. The manuscript by Peyran et al. first used bem1 cdc24lip anionic lipid binding mutants to induce defective polarity establishment. The results with bem1 cdc24lip mutants demonstrate that polarity defects stabilize G1 cyclin Cln2 as well as Swe1 kinase, which prevents mitotic progression by phosphorylating and inhibiting CDK1. In this study, bem1-AID cdc24lip mutants were also generated to further strengthen the connection between polarity and G1 cyclin, and between G1 cyclin and Swe1. A particular interesting observation is from the phospho-deficient and phospho-mimetic cln2 mutants, showing that Cln2 phosphorylation prevents its nuclear localization and degradation, while also sustaining CDK1 phosphorylation by Swe1. However, this manuscript lacks sufficient mechanistic details about the link between Cln2 phosphorylation and Swe1 stabilization. Moreover, 24 residues were mutated to generate

cln2 phospho-mutants, leading to ambiguity. Results from specific cln2 phospho-mutants may further clarify the role of each phosphorylation event in the regulation of Swe1 stability and cell cycle progression. Therefore, major revision is required before this manuscript can be considered by Nature Communication.

Major points

1. The conclusion from Fig. 2 is that increased Cln2 phosphorylation and extended expression in bem1 cdc24lip cells is caused by defective polarized growth, but not an indirect consequence of Swe1 checkpoint activation. The evidence supporting this conclusion is relatively weak. To exclude the role of Swe1 in regulating Cln2, one possible experiment is to examine Cln2 protein level and modification in WT cells overexpressing Swe1.

We previously concluded that Cln2 phosphorylation and stabilization in response to polarity defects were not an indirect consequence of $Swe1^{Wee1}$ checkpoint activation. We arrived at this conclusion by mutating Tyr19 in $Cdc28^{Cdk1}$ that $Swe1^{Wee1}$ phosphorylates to inhibit $Cdc28^{Cdk1}$ during checkpoint activation. However, we acknowledge the reviewer's concern that this point could be strengthened. We therefore induced polarity defects in a *swe1Δ* mutant and looked at Cln2 stability and phosphorylation. Consistent with the results in the *$cdc28^{cdk1}$ Y19F* mutant, Cln2 was also hyperphosphorylated and expressed for longer in the *swe1Δ* mutant when cells experienced polarity defects using the *$bem1^{lip}$ $cdc24^{lip}$* mutant. These results are shown in Fig. 3B, which also now includes the requested blots for $Swe1^{Wee1}$ and supporting FACS analysis in Supplementary Fig. 6c. Samples from these lysates were also run on the same gel for side-by-side comparison in Supplementary Fig. 6b, as requested by reviewer 2. The reviewer also suggested the possibility of looking at Cln2 during $Swe1^{Wee1}$ overexpression. However, this experiment would not address whether Cln2 stabilization occurs independently of $Swe1^{Wee1}$ checkpoint activity. Indeed, we previously found that Cln2 is stabilized after $Swe1^{Wee1}$ overexpression and that this promotes excessive polarized growth in a $Cdc28^{Cdk1}$-dependent manner (McCusker et al. 2007, PMID: 17417630 Figs. 1c, d and S1c). The most direct means of assessing whether Cln2 stabilization is independent of the $Swe1^{Wee1}$ checkpoint is to inactivate the checkpoint. We hope the reviewer will be convinced that Cln2 stabilization occurs independently of this checkpoint after our use of two independent methods to inactivate the checkpoint that give the same result.

2. G1 cyclin signaling contributes to Swe1 activity (Fig. 4). This figure shows persistent Cdc28 phosphorylation by Swe1 in bem1-AID cdc24lip mutants expressing Cln2-Asp, but cells expressing Cln2-Ala exhibit a slightly faster disappearance of Y19-P Cdc28 compared to control cells. Although these results indicate that Cln2 phosphorylation affects Swe1 activity, it is unclear if this effect is direct or indirect. Swe1 degradation requires its bud-neck localization. It might be worthwhile to examine polarity establishment in bem1-AID cdc24lip mutants expressing Cln2-Asp or Cln2-Ala, because one possibility is that expression of Cln2-Asp further exacerbates polarity establishment, which indirectly stabilizes Swe1. As a control, the polarity establishment in WT cells (but not bem1 cdc24lip mutants) expressing Cln2-Asp or Cln2-Ala need to be examined. In addition, Swe1 phosphorylation and protein levels should be included in this figure, because this result will be informative for the connection between Cln2 phosphorylation and Swe1 phosphorylation/stability.

The reviewer's point is well taken that we do not currently know all of the molecular events linking Cln2 phosphorylation to $Swe1^{Wee1}$ activation. It was not our intention to claim that Cln2 directly regulates $Swe1^{Wee1}$; it may, but this remains to be tested. We have therefore modified the figure legend to reflect this uncertainty. The title is now, "G1 cyclin signalling delays

anaphase onset to protect cells from inappropriate nuclear division". In addition, we have now conducted the requested experiments.

First, the reviewer asked if *cln2-Asp*, which we have now renamed *cln2-24D*, may exacerbate the polarity defects of the *bem1^{lip} cdc24^{lip}* mutant and suggests monitoring polarity establishment in this mutant. These experiments are presented in Fig. 4I where we see that *cln2-24D* rescues the polarity defects of the *bem1^{lip} cdc24^{lip}* mutant. We attribute this rescue to increased cytoplasmic Cln2 in *cln2-24D* cells which provides augmented G1-cyclin activity to boost Cdc42 activation to a level that can support stable polarity axis establishment. This point is supported by the longer duration of Cdc42 GAP hyperphosphorylation in *bem1^{lip} cdc24^{lip} cln2-24D* cells (see Rga2 blots in Fig. 4d) and by the cytoplasmic enrichment of cln2-24D-sfGFP (Figs. 5c and e). As requested, we have also now included Swe1^{Wee1} blots in this figure (Figs. 4a-d). The blots show that Swe1^{Wee1} is hyperphosphorylated at 75 minutes and destabilized in WT cells, while displaying less phosphorylation and more stabilization in the *bem1^{lip} cdc24^{lip}* mutant. In the *cln2-24A* mutant Swe1^{Wee1} is eventually destabilized and Cdk1 Tyr19 phosphorylation levels fall, while in *cln2-24D* cells both Swe1 stabilization and inhibitory Cdk1 Tyr19 phosphorylation are prolonged. This data, together with our imaging data discussed below, support our proposal that Cln2 phosphorylation, which is mimicked in the *cln2-24D* mutant, contributes to its cytoplasmic localization where it activates Cdc42. In addition to this spatial regulation of Cdc28^{Cdk1} activity, phosphorylated Cln2 boosts Swe1^{Wee1} checkpoint activity, providing temporal control of the cell cycle. We propose that collectively, this spatial and temporal regulation of Cdc28^{Cdk1} activity constitutes a corrective response to polarity defects.

We also present a series of experiments in Supplementary Fig. 10 a-f examining *cln2-24A* and *cln2-24D* in otherwise WT *BEM1* cells. Budding occurred with similar timing in all of the strains tested, indicating that *cln2-24D* mutants do not induce strong polarity establishment defects (Supplementary Fig. 10c). However, Tyr19 phosphorylation levels on Cdc28^{Cdk1} were slightly elevated in *cln2-24D* cells. This may be because *cln2-24D* mutants displayed a slight "hyperpolarized" morphology in *BEM1* cells where the cells are more ovoid (Supplementary Figs. 10e and f). This has previously been observed in cells with increased Cdc42-GTP due to deletion of Cdc42 GAPs, and in cells overexpressing Cln2 (Caviston et al. 2003, PMID: 14517318 Figure 3; Lew and Reed 1993, PMID: 8449978 Figure 6). This phenotype is consistent with our observations that the *cln2-24D* mutant displays prolonged Rga2 GAP hyperphosphorylation, which inactivates the GAP (Knaus et al. 2007, PMID: 17914457 Figure 6). We also observe that cln2-24D protein is expressed for longer during the cell cycle. We tested whether this was due to stabilization by looking at the half-life of the protein using Gal shut-off experiments where a burst of transcription from the *GAL1* promoter is terminated after dextrose addition (Supplementary Fig. 9). The half-lives of Cln2, cln2-24A and cln2-24D were similar in these *BEM1* cells (15 minutes, 9 minutes and 18 minutes respectively). This contrasts with cln2-4T3S, which had a half-life of > 40 minutes (Supplementary Figs. 7d and e). It therefore appears more likely that the increased expression of cln2-24D in these *BEM1* cells reflects higher rates of transcription. Future experiments will be required to identify how cln2-24D stimulates its own expression.

3. The cln2-Asp and cln2-Ala mutants. In these mutants, 24 amino acid residues are replaced with either Ala or Asp based on phosphorylation sites identified by MS, including two CDK sites. Previous results show that CDK1-dependent Cln2 phosphorylation regulates its localization and degradation. It would be informative to generate cln2 mutants with mutated CDK phosphorylation sites and further examine their localization and polarization phenotypes. It seems that cln2 mutants lacking CDK phosphorylation sites are available. It is unclear if Swe1 activity has been examined in these mutants.

We thank the reviewer for this suggestion. In addition to the analysis of cln2-24A and cln2-24D-sfGFP dynamics in Fig. 5, we also present additional analysis focusing on the contribution of different phosphorylation sites to Cln2 nucleo-cytoplasmic partitioning, including Cdc28^{Cdk1}

consensus site mutants that stabilize the protein (*cln2-4T3S*), which will be discussed below. In these experiments, which are presented in Supplementary Fig. 12, we expressed Cln2-sfGFP constructs from the conditional *MET25* promoter. Cells were given a pulse of Cln2-sfGFP expression by withdrawing methionine then methionine was added to repress transcription and imaging was started. Whereas wild type Cln2-sfGFP was nuclear and cytoplasmic in *BEM1* cells, in the $bem1^{lip}$ $cdc24^{lip}$ mutant the protein was enriched in the cytoplasm, as we reported using expression from the *CLN2* promoter in Fig. 5. We next sought to distinguish Cln2 phosphorylation sites that are important for localization from $Cdc28^{Cdk1}$ consensus sites that stabilize the protein. We generated a mutant in which all phosphorylation sites except $Cdc28^{Cdk1}$ consensus sites were changed to alanine (*cln2-22A*) and transformed this into the $bem1^{lip}$ $cdc24^{lip}$ mutant. Importantly, this mutant was enriched in the nucleus and did not display the strong cytoplasmic localization that WT Cln2 displayed in $bem1^{lip}$ $cdc24^{lip}$ cells (Supplementary Figs. 12a and f). This indicates that the phosphorylation of these non-$Cdc28^{Cdk1}$ consensus sites are necessary for the cytoplasmic enrichment of Cln2 when polarity problems are encountered.

We next addressed the localization of *cln2* mutants that are stabilized due to the mutation of $Cdc28^{Cdk1}$ consensus sites, referred to as *cln2-4T3S* (Lanker et al. 1996 PMID: 8599119). In *BEM1* cells, cln2-4T3S-sfGFP was predominantly nuclear, with some cytoplasmic localization that is polarized to the bud (Supplementary Figs.12b and g). This result is important because one of the questions raised during review was whether the predominantly cytoplasmic localization of Cln2 that we observed in the $bem1^{lip}$ $cdc24^{lip}$ mutant shown in Fig. 5a may simply reflect its stabilization. This is not the case, as stabilized *cln2-4T3S-sfGFP* is predominantly nuclear in *BEM1* cells (Supplementary Fig. 12b, upper panels). Moreover, when we imaged *cln2-4T3S-sfGFP* dynamics in the $bem1^{lip}$ $cdc24^{lip}$ mutant, we observe that the protein accumulates strongly in the cytoplasm with some nuclear localization (Supplementary Fig. 12b bottom panels). The data therefore point to cln2-4T3S being phosphorylated on non-$Cdc28^{Cdk1}$ consensus sites in the $bem1^{lip}$ $cdc24^{lip}$ mutant, triggering its cytoplasmic accumulation.

The reviewer also asked about the polarization phenotype of the *cln2-4T3S* mutant. In *BEM1* cells, a pulse of expression of *cln2-4T3S* from the *MET25* promoter generated ovoid cells due to excessive polarized growth similar to *cln2-24D* in WT, as can be seen in the cell shown in Supplementary Figure 12e. However, the percentage of cells that form a bud and the timing between *MET25* promoter repression and budding was similar in *CLN2* and *cln2-4T3S* cells (Supplementary Figs. 12c and d). Finally, as requested, we also compared $Cdc28^{Cdk1}$ Tyr19 phosphorylation in *CLN2* and *cln2-4T3S* cells in the $bem1^{lip}$ $cdc24^{lip}$ mutant. The reviewer will see that the levels are comparable. We have marked the relevant lanes for comparison with an asterisk in the figure below. We did not include this figure in the resubmitted manuscript as we wanted to keep this section of the manuscript focused on the role of Cln2 phosphorylation in nucleo-cytoplasmic partitioning. However, if the reviewer feels that it would make a valuable addition, we can include it.

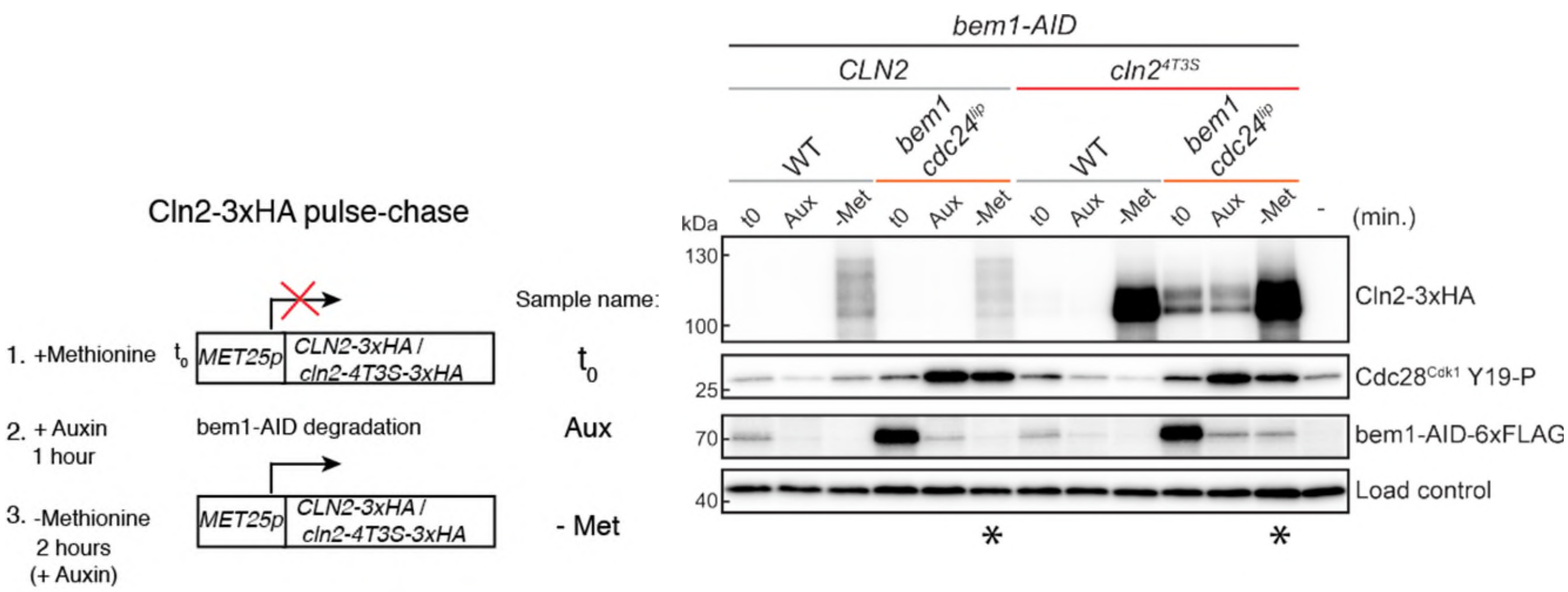

4. The logic for Fig. 3 is not easy to follow. It would be better to include a diagram showing the two models. Also, the text for this figure needs to be reworded with a clear rationale for the experimental design. A clear increase in Cln2 protein level and delayed Cln2 band-shift are noticed in Fig. 3A, and this should be taken into account when drawing conclusions.

In the resubmitted manuscript we have now provided a diagram with the model being tested in Fig. 3a, clearer rationale for the experiments being presented (page 8, lines 232-236) and analysis of Cln2 phosphorylation states on the same gel in Supplementary Fig. 6d. On this gel the Cln2 electrophoretic mobility shift is essentially indistinguishable between *bem1^{lip} cdc24^{lip}* and *bem1^{lip} cdc24^{lip} cdc28^{cdk1} Y19F.* However, we agree that the kinetics of Cln2 hyperphosphorylation exhibits some variability in the Swe1^{Wee1} checkpoint mutants (*cdk1^{cdc28} Y19F* and *swe1Δ*) and we have mentioned this in the text on page 8, lines 242-243.

Minor points:

-There is no page number.
We apologize for this omission in the initial submission and have corrected it.
- The manuscript would benefit from English editing, as the logical flow is sometimes difficult to follow.
This has been done. However, if the reviewer still finds instances where the text should be modified then please let us know.
-A better description of yeast strain bem1 cdc24lip is necessary in the main text. Also, the strain name bem1lip cdc24lip for this mutant seems more appropriate based on the description.
The name of the mutant has now been changed to *bem1^{lip} cdc24^{lip}* throughout the manuscript. In addition, we have clarified the description of the mutant when it is first mentioned in the text (page 4, lines 92-94) in addition to providing a supplementary figure that provides at atomic model of the proteins showing the positions of the charge-swap point mutations.
-Figure 1A: More information is needed to describe how to define cell separation as time 0.
When budding yeast separate at the end of cell division, daughter cells exhibit a noticeable physical displacement evident as a rotation by DIC imaging. Additionally, we monitor division using Sec3-GFP, the exocyst marker that tethers the post-Golgi vesicles to the plasma membrane required for cell division. These points have now been clarified in the text on page 4, lines 95-96).
-Figure 1E: Why is the faster nuclear release of Whi5 observed in bem1 cdc24lip cells? Is this phenotype dependent on Cln3-Cdc28 activation?
We consistently see faster nuclear Whi5 release from the nucleus in *bem1^{lip} cdc24^{lip}* mutant cells compared to *BEM1* controls. This is the case in synchronized cell populations (Figs. 1d and e) and when dynamics are monitored in non-synchronized individual cells (Supplementary Figs. 1d and e). While we attribute this effect to higher G1 cyclin activity that is generated during the adaptive response to polarity defects, we have not yet been able to hone in on the specific cyclin responsible.
-It seems that bem1 cdc24lip cells exhibit a significant defect in polarity establishment, thus, slow cell growth is expected. It is unclear how these mutant cells are synchronized in G1 phase in Fig. 1f.
In all experiments involving synchronization we employed alpha-factor to induce a G1-like arrest before synchronous release into the cell cycle. We carried out controls to ensure that *bem1^{lip} cdc24^{lip}* mutant cells arrest in G1 in response alpha-factor including looking at their sensitivity by "halo" assays of growth inhibition at different alpha-factor concentrations (Supplementary Fig. 2a). In the resubmitted manuscript we also provide FACS analysis showing a G1 profile for alpha-factor treated cells (Supplementary Fig. 6c). In the resubmitted manuscript we have stated in the legend to Fig. 1f that alpha-factor was used for synchronization (page 31 lines 865-866).

-A brief explanation of cdc28 Y19F is needed when it is mentioned for the first time (Fig. 2b, page xxx).

Thank you for pointing this out. This has been done on pages 7-8, lines 209-212.

-Description for supplementary Fig. 2D could not be found in the main text.

This has been provided.

- "Moreover, the phosphorylation that we observe in the bem1 cdc24lip mutant is associated with an increased window of Cln2 expression rather than its destabilization". My understanding is that destabilization of Cln2 is an important factor in controlling Cln2 expression window. This needs to be reworded.

Thank you. This has been clarified.

-Figure 5. It will be interesting to examine the localization of Cln2 with mutated CDK phosphorylation sites. This will directly detect the role of Cln2 phosphorylation by CDK in its localization and degradation.

This was a really helpful suggestion for which we are grateful. As discussed in Major point 3, we generated *cln2-4T3S*-sfGFP and analyzed the dynamics of its localization after providing cells with a pulse of expression using the conditional *MET25* promoter (Supplementary Figs. 12b, c, d, e and g). Comparison of Cln2-sfGFP with cln2-4T3S-sfGFP underscores the precision with which Cln2 is usually degraded after budding (compare top panels in Supplementary Figs. 12 a and b). We observe that cytoplasmic cln2-4T3S is strongly enriched at the site of bud growth, driving bud growth long after the WT protein is degraded and polarized bud growth ceases. The effect of this is to generate the ellipsoid cell shown in Supplementary Fig. 12e (bottom panel). The extent of the co-localization between cln2-4T3S and the growing bud has not been reported previously and illustrates the key role that G1 cyclin regulation plays in polarized growth.

Reviewer #2 (Remarks to the Author):

This manuscript tackles a fundamental question in cell biology: how cells maintain cell cycle fidelity in the face of polarity disruption. The authors present compelling data showing that polarity defects lead to a loss of coordination between nuclear and cytoplasmic functions of G1 cyclin-CDK, and widespread disruptions to cell cycle timing. A particularly well-supported finding is the increased activity of Swe1, which delays cell cycle progression and may provide time for error correction. These aspects of the study are clearly demonstrated and provide a solid foundation for the manuscript. Building on this, the authors propose that regulation of the G1 cyclin Cln2 is central to this response, suggesting that Cln2 phosphorylation relays signals to Swe1 to delay the cell cycle, and contributes to error correction via enhanced phosphorylation of the GTPase regulators. However, these conclusions are less well supported. Much of the data is correlative and does not directly test the proposed model. To substantiate these claims, two major questions need to be addressed: (1) How is Cln2 expression prolonged in response to polarity disruption, and what are the relative contributions of transcriptional regulation versus phosphorylation? (2) Are the observed changes in Cln2 expression sufficient to activate Swe1 and delay the cell cycle? In summary, while the manuscript presents a promising and conceptually interesting model, additional mechanistic data are required to validate the proposed role of Cln2 in coordinating the polarity response.

Thank you for these encouraging comments and thoughtful questions. In the resubmitted manuscript we performed additional experiments to address these points and hope to have provided a clearer mechanistic link between Cln2 phosphorylation, its localization and checkpoint signaling. This is explained in the specific points below.

Major concerns

1. With the exception of Fig. 1A–C, all experiments in the manuscript rely on alpha-factor arrest and release to investigate cell cycle differences in mutant strains. However, alpha-factor not only arrests cells in G1 but also induces polarization, resulting in shmoo formation. Consequently, cells polarize during the arrest phase, are then released, and subsequently undergo re-polarization—raising the question of whether this sequence could influence the observed results. Importantly, synchronization may not be essential for several of the experiments presented. As demonstrated in Fig. 1A, imaging asynchronous cells and analyzing only those that enter G1 during the experiment is a viable alternative. This caveat of alpha factor should be mentioned, and it would strengthen the manuscript to validate key findings in asynchronous cells. For example, if fluorescent Whi5 was included in the experiment shown in Fig. 1A, would the results match those in Fig. 1D–E? Similarly, do the findings in Fig. 2B–C hold when cells are not pre-synchronized with alpha-factor?

We acknowledge the reviewer's concern on this point and have now performed numerous additional imaging experiments in non-synchronized cells to ensure that the phenotypes observed in the *bem1*^{lip} *cdc24*^{lip} mutant are not due to alpha-factor synchronization. We also confirmed that the time from cell separation to Whi5 nuclear exit is advanced in the *bem1*^{lip} *cdc24*^{lip} mutant (Supplementary Figs. 1d and e).

Additionally, using live-cell imaging in asynchronous cells we observe a delay in the time from cell separation to Myo1 polarization in the *bem1*^{lip} *cdc24*^{lip} mutant (Supplementary Fig. 3a-c). We also confirmed that Clb2 expression was prolonged and anaphase was delayed in the *bem1*^{lip} *cdc24*^{lip} mutant (Supplementary Fig. 3d). The imaging of spindle dynamics in Supplementary Fig. 3e was also done in asynchronous cells.

Since these experiments were performed using the constitutive expression of bem1^{lip} cdc24^{lip} mutants, we also provide controls that similar phenotypes are observed using the AID system in asynchronous cultures. This demonstrates that the observed phenotypes were not due to long-term checkpoint adaptation. These controls are provided in Supplementary Fig. 5c (Sec3 using the AID system), Supplementary Fig. 5d (time from cell separation to bud emergence using the AID system), Supplementary Figs. 5e-g (Clb2 and SPB dynamics using the AID system) and Supplementary Fig. 5h (appearance of multinucleate cells using the AID system).

2. In both Fig. 3A and Fig. 4A-D, it's difficult to compare the different mutants directly due to how the experiments are presented. First, because the samples from different strains are run on separate gels, it's not possible to draw reliable conclusions about relative expression levels or phosphorylation states of the different proteins across mutants. While lane availability is understandably limited, including select time points (e.g., 15, 30, and 60 minutes) from each strain on the same gel would allow for direct comparison and better support the authors' claims about relative expression and phosphorylation.

Thank you for this suggestion which now enables direct comparison of the phosphorylation mobility shifts between different mutants. These results are now presented in Supplementary Figs. 6b and 8j.

3. Second, in Fig. 3A and 4A-D, the interpretation of the data would benefit from flow cytometry analysis of DNA content to assess synchrony in the release experiments across strains. Without this, it's unclear whether differences in marker expression reflect true biological differences or simply variation in cell cycle progression—for example, if a subset of cells in one strain is delayed in a particular phase, it could give the appearance of prolonged marker expression.

Flow cytometry analysis is now presented in Supplementary Figs. 6c and 8i. The different strains appear to be well synchronized since they transit from 1C to 2C DNA content comparably. The reviewer also asks whether the differences in marker expression reflect true biological differences. This is a great question and one that originally motivated us to perform the single cell analysis of cyclin dynamics. To our knowledge, this has not previously been reported for G1 cyclins and the technical challenge that we encountered was the identification of fluorophores that were sufficiently bright and matured more rapidly than the rate of degradation of Cln2, an unstable, low abundance protein. In the case of Cln2 expression, we feel that the data on this point is unambiguous: in the *bem1*^{lip} *cdc24*^{lip} mutant Cln2-sfGFP displays an extended wave of expression when analyzed at the single cell level by imaging and in a cell population by western blotting (Figs. 5a, 3b and 4b). The extended activity of Cln2 during the cell cycle is also borne out by looking at the phosphorylation of the Cln2 substrate Rga2. Given the consistency of the conclusion using different approaches, we think these data are indicative of an adaptive response to polarity defects. We also observed higher steady-state Cln2-sfGFP expression in single cells experiencing polarity defects in non-synchronized cells (Supplementary Fig. 11a). The same applies to Clb2; both by western blotting in a cell population and analysis of Clb2-sfGFP dynamics in single cells, mitotic cyclin stabilization is evident (Supplementary Fig. 5e). The biological difference between Clb2 dynamics in WT cells and the *bem1*^{lip} *cdc24*^{lip} mutant derives from Swe1^{Wee1} checkpoint activity in the mutant that delays Clb2 degradation and mitotic exit.

4. The increase in Cln2 half-life in the polarity mutant is quite modest compared to the well-characterized, stable 4T3S mutant. Given the significant disruption to cell cycle dynamics, it seems likely that the prolonged Cln2 expression observed is primarily due to altered transcription rather than increased protein stability alone. To test this, Cln2 mRNA levels should be measured in the experiment shown in Fig. 3A. If transcription is elevated or prolonged in the mutant, it could explain the increased protein levels. Conversely, if mRNA levels are comparable between mutant and wild-type cells, this would strongly support a role for Cln2 phosphorylation and/or stability in the polarity sensing mechanism.

The reviewer is correct that the stabilization of Cln2 in the *bem1^lip^ cdc24^lip^* mutant is more modest than that of *cln2-4T3S*. The prolonged expression of Cln2 in the mutant was therefore also likely to reflect increased transcription. It was not our intention to convey the idea that transcription plays no role in the response. We anticipated that Cln2 mRNA might be elevated in polarity mutants because it is embedded in a feedforward loop between Cln2 and the G1 transcriptional regulon that upregulates gene expression (Cross and Tinkelenberg 1991, PMID: 2040016; Dirick and Nasmyth 1991, PMID: 1829507; Skotheim et al. 2008, PMID: 18633409). We quantified Cln2 mRNA by RT-pPCR and observe a burst of mRNA that persists in the *bem1^lip^ cdc24^lip^* mutant. This data is presented in Supplementary Fig. 7f with explanatory text on page 10, lines 280-285. Both transcriptional and post-translational mechanisms therefore operate to provide the burst of Cln2 protein observed *in vivo*. The employment of both mechanisms likely makes G1 more robust. Thank you for focusing our attention on this point.

5. The rationale for the Cln2 phosphomutants tested in this study is not clearly established, which makes it challenging to interpret the experimental results. The text suggests that the authors mutated all phosphorylation sites previously identified under various conditions from large-scale proteomic studies, rather than focusing on sites specifically phosphorylated during polarity disruption. Among these, two are known CDK sites that influence Cln2 degradation, but the kinases responsible for the remaining sites are either unknown or not discussed. To convincingly link polarity defects to changes in Cln2 phosphorylation and expression—an effect the authors propose as causative for the observed cell cycle delay—it would be important to first identify which Cln2 phosphosites are actually altered under polarity-disrupted conditions. Targeted mutation of those specific sites would strengthen the mechanistic connection. Additionally, generating separate mutants for CDK versus non-CDK sites could provide insight into which kinases may regulate Cln2 in the context of polarity disruption.

We have now edited the text on page 10, lines 286-302 to introduce the logic for the phosphosite mutants more clearly for readers. We agree that purifying Cln2 from the *bem1^lip^ cdc24^lip^* mutant and identifying sites by mass spectrometry would have been the ideal experiment, however this option was not available to us so we analyzed our own mapping data on Cln2 sites from WT cells and the available proteomic data. We were surprised by both the high number of phosphorylated sites in Cln2 and the low number of Cdc28^{Cdk1} consensus sites (only two), suggesting that Cln2 phosphorylation had functions in addition to the generation of the degron recognized by SCF^{Grr1}. We had also observed that while the electrophoretic mobility shift of Cln2 in the *bem1^lip^ cdc24^lip^* mutant occurred faster than in WT cells, the extent of the shift was nevertheless similar, suggesting that some of the sites phosphorylated in WT cells might overlap with those in *bem1^lip^ cdc24^lip^* mutant. This point is supported by the *cln2-24A* mutant, which strongly reduces the electrophoretic mobility shift of Cln2 *in vivo,* indicating that relevant sites have been mutated (Supplementary Fig. 8j). While most of the electrophoretic mobility shift has been ablated in the *cln2-24A* mutant, the presence of a minor residual shift indicates that some additional sites remain unidentified, and we have pointed out this caveat in the manuscript on page 11, lines 317-320.

We do not currently know which kinase phosphorylates Cln2 when polarity defects are encountered, but we are working on this important question. It is also worth mentioning that the identity of the kinase that phosphorylates Cln2 in WT cells has not been formally identified. While it is assumed to be Cdc28^{Cdk1} because Cln2 contains 7 Cdc28^{Cdk1} consensus sites, only one of these conforms to a full Cdc28^{Cdk1} consensus site, raising the possibility that other proline-directed kinase(s) may target some of the other 6 sites. Clearly there is more to learn about the phosphorylation of Cln2.

In order to understand how phosphorylation influences Cln2 function, we conducted the experiments suggested by the reviewers. We generated *cln2-22A* in which only non-

Cdc28^{Cdk1} sites were mutated to alanine and *cln2-4T3S* in which all 7 Cdc28^{Cdk1} consensus sites are mutated to alanine. We expressed the sfGFP-tagged proteins from the *MET25* promoter where expression is induced when methionine is removed (Supplementary Fig. 12). Cells were provided with a pulse of expression by removing then adding back methionine. We then immediately monitored the dynamics of the sfGFP-proteins. Cln2-sfGFP was nuclear and cytoplasmic in *BEM1* cells, while displaying marked cytoplasmic accumulation in the *bem1^{lip} cdc24^{lip}* mutant (Supplementary Fig. 12a). The system therefore recapitulated the results we reported using the endogenous *CLN2* promoter (Fig. 5). We observed that cln2-22A-sfGFP displayed reduced cytoplasmic and elevated nuclear localization in *bem1^{lip} cdc24^{lip}* mutant, indicating that the phosphorylation of Cln2 on non-Cdc28^{Cdk1} sites is important for its accumulation in the cytoplasm in response to polarity defects (Supplementary Figs. 12a, bottom panel and f).

As suggested by the reviewer, we also generated the cln2 mutant in which Cdc28^{Cdk1} consensus sites were mutated (*cln24T3S*). cln2-4T3S displayed marked stabilization that defined the site of growth and strong nuclear enrichment in *BEM1* cells (Supplementary Fig. 12b top panel). In the *bem1^{lip} cdc24^{lip}* mutant where cln2-4T3S could still be phosphorylated on non-Cdc28^{Cdk1} sites, cln2-4T3S became strongly enriched in the cytoplasm, underscoring the profound reconfiguration of G1 cyclin nucleo-cytoplasmic partitioning in response to polarity defects via Cln2 phosphorylation on non-Cdc28^{Cdk1} sites (Supplementary Figs. 12b bottom panel and g).

6. The functional consequences of the cln2-Ala and cln2-Asp mutants remain unclear and warrant more thorough characterization. First, it is important to assess whether either mutant affects Cln2 protein stability. The Asp mutant appears to be expressed at higher levels and persists longer, which is unexpected given that phosphorylation typically targets Cln2 for degradation. One possible explanation is that phosphomimetic mutations often fail to recreate the structural interface required for recognition by SCF ubiquitin ligases, thereby impairing degradation. However, if this were the case, the Ala mutant—which prevents phosphorylation—might be expected to behave similarly, raising further questions. Second, the data in Figure 4 do not clearly demonstrate whether the mutations affect Cln2 localization and whether it is localization that confers the phenotype. The apparent cytoplasmic accumulation of the Asp mutant may simply reflect its elevated expression level rather than a true change in localization. If WT Cln2 or Cln2-Ala are relocalized to the cytoplasm do they phenocopy the Asp mutant?

The reviewer's question on cln2-Ala and cln2-Asp can be summarized in 3 parts: do these mutations affect protein stability? Do the mutations affect localization? If we think that they do affect localization, can we modify this localization to convincingly demonstrate our proposal that polarity defects reconfigure the spatial organization of G1 cyclins?

i) The stability of *cln2-24A* and *cln2-24D*.

We tested the half-life of the proteins using Gal shut-off experiments where a burst of transcription from the *GAL1* promoter is terminated after dextrose addition (Supplementary Fig. 9). The half-lives of Cln2, cln2-24A and cln2-24D were comparable (15 minutes, 9 minutes and 18 minutes respectively). This contrasts with cln2-4T3S, which had a half-life of > 40 minutes (Supplementary Figs. 7d and e). Thus, the phosphorylation site mutants do not appear to strongly alter the intrinsic stability of the proteins in *BEM1* cells. Therefore, in *bem1^{lip} cdc24^{lip}* mutants, the extended half-life of Cln2 suggests that SCF^{Grr1} may be less active (Supplementary Figs. 7a-c).

ii) Do *cln2-24A* and *cln2-24D* affect localization?

The reviewer was concerned that the proposed cytoplasmic enrichment of cln2-24D may reflect elevated expression rather than a true change in localization. We address this valid concern with three lines of evidence that we feel support our initial proposal. First, if the localization of cln2-24D only reflected elevated expression and not a response to polarity defects then cln2-4T3S, which is stabilized, ought to have a similar localization in WT and *bem1^{lip} cdc24^{lip}* mutants. However, Supplementary Figs. 12b and g indicate that this is not the case and that cln2-4T3S-sfGFP behaves differently in *bem1^{lip} cdc24^{lip}* mutants compared to its pattern in *BEM1* control cells. Second, we provide evidence that the reorganization of Cln2 from the nucleus to the cytoplasm in response to polarity defects requires the phosphorylation

of Cln2 on non-Cdc28^{Cdk1} sites, since mutation of these sites in the *cln2-22A* mutant strongly perturbs cytosolic Cln2 accumulation, while clear nuclear localization is evident (Supplementary Fig. 12a, bottom panel). Third, comparison of cln2-24A and cln2-24D in Fig. 5c show that they display opposite dynamics, which would not be expected if the only difference between the proteins was expression level. Fluorescence is excluded from the nucleus in the cln2-24D mutant after 105 minutes, whereas fluorescence accumulates in the nucleus transiently in the cln2-24A mutant. We feel that this data, and the next point below provide a strong case that G1 cyclin dynamics are rewired in response to polarity defects.

iii) Modifying cln2 localization.
We placed a nuclear export sequence on cln2-24A to generate cln2-24A-NES and tested if this would generate sufficient cytosolic G1 cyclin activity to correct polarity defects in the *bem1^{lip} cdc24^{lip}* mutant. Figs. 5c and g demonstrate the cytosolic enrichment of cln2-24A-NES and its nuclear exclusion, as was observed for cln2-24D. In the upper cln2-24A-NES cell shown in Fig. 5c the cytosolic targeting of cln2-24A was indeed sufficient to correct the polarity defect and enable budding. In the cell shown in the lower panel polarity defect is corrected during imaging, indicated by the evolution of the cln2-sfGFP signal from a non-polarized to polarized state, but the movie was stopped before budding. Fig. 5g provides quantification of the positive effect that cln2-24A-NES had on the budding in the *bem1^{lip} cdc24^{lip}* mutant. Thank you for suggesting this experiment to us.

7. Additional experiments are needed to establish a causal relationship between increased Cln2 levels or activity and Swe1 activation. Specifically, does overexpression of Cln2, or expression of a phosphomimetic mutant targeting relevant sites, lead to Swe1 activation in the absence of polarity disruption? Directly testing this would strengthen the proposed model and clarify whether Cln2 alone is sufficient to trigger this aspect of the checkpoint response.

We address this question in Supplementary Fig. 10 a-c, where we analyzed cell cycle progression in *cln2-24A* and *cln2-24D* in otherwise WT *BEM1* cells. Bud emergence occurs at the same time in both strains (Supplementary Fig. 10c). However, prolonged cln2-24D expression results in slightly hyperpolarized cells, illustrated by cells becoming more ovoid (Supplementary Figs. 10e and f). This phenotype is likely due to excessive Cdc42 activation stemming from Cdc28^{Cdk1}-dependent GAP inactivation (Knaus et al. 2007, PMID: 17914457; Sopko et al. 2007, PMID: 17853895). This phenotype supports our proposal that cln2-24D displays heightened activity towards Cdc42 regulators in the cytosol.

We also observe that inhibitory Tyr19 phosphorylation on Cdc28^{Cdk1} is prolonged in *cln2-24D* cells compared to *cln2-24A* cells and that Clb2 degradation occurs slightly later in the *cln2-24D* mutant (Supplementary Figs. 10a and b). These points are also supported by FACS profiles where the 2C peak in *cln2-24D* persists after it has receded to 1C in *cln2-24A* (Supplementary Fig. 10d, 150 min). The changes in cln2 expression in the *cln2-24D* mutant are therefore sufficient to activate Swe1^{Wee1} and delay mitotic progression, an effect that is greatly amplified when strong polarity defects are encountered.

Additional comments
8. The introduction states "The irreversibility of cell cycle commitment is thought to be mediated by a positive feedback loop where the G1-cyclin Cln3-Cdc28Cdk1 phosphorylates and partially inhibits Whi5...". More recent work shows that Cln3 doesn't directly phosphorylate Whi5 (Koivomagi et al, Science 2021; PMID: 34648313). This statement should be revised.

Thank you for pointing this out. We have modified the text on page 2, lines 25-28 accordingly.

9. The text states "the resulting polarity defects also triggered a Swe1Wee1-dependent mitotic delay via inhibitory phosphorylation of Cdc28Cdk1 on Y19 (Supplementary Fig. 1i)". It is not clear what the purpose of Sup Fig 1i is, but it does not support this statement.

Supplementary Fig. 1i showed that while individual point mutations in Bem1 and Cdc24 lipid binding domains did not activate the Swe1 checkpoint, mutation of all the lipid sites did. This had been included to illustrate how multivalency in lipid binding is sensed by the Swe1^{Wee1} checkpoint. However, in the resubmitted manuscript this figure was not included.

10. Many figures require more detail about the number of biological replicates performed (Figs. 1B, 1E, 1I, 2C, 3B, 4F, 4H, 5B, 5D-F). It should also be stated whether cells from multiple replicates have been pooled together to generate the plots.

This data has now been provided in the figure legends, methods and accompanying reporting summary.

11. Fig 2D-E and 4I should include error bars.

In our initial submission, cells from multiple replicate experiments conducted on different days were pooled to generate the plots. In the resubmitted manuscript we have presented the data with error bars, as requested.

12. It is not clear whether any mass spectrometry analysis was performed in this study to map Cln2 phosphosites. There is a section in the methods describing a purification/mass spectrometry experiment, but no results from such an experiment are presented in the manuscript, and the text suggests that the phosphosite mutants that are tested were designed based on other published, large-scale datasets. If this experiment was performed, it should be included.

In Supplementary Fig. 8d, we show the sites that were identified in our mass spectrometry analysis and a summary of the sites identified by other studies.
