# Supplementary material for: A cyclin-polarity feedback network ensures healthy cell proliferation
Source: Nat Commun. 2026 Jun 19;17:7731. doi: 10.1038/s41467-026-74596-7 (PMC13434654; doi:10.1038/s41467-026-74596-7)
Supplement: Supplementary file 3 [file 41467_2026_74596_MOESM1_ESM.pdf]

A cyclin-polarity feedback network ensures healthy cell proliferation

Landry Peyran[1], Charles Lefranc[1], Steven P. Gygi[2], Anne Royou[1] and Derek McCusker[1]

[1] Univ. Bordeaux, CNRS, IBGC, UMR 5095; F-33000 Bordeaux, France.

[2] Department of Cell Biology, Harvard Medical School; Boston, MA, USA.

Supplementary information

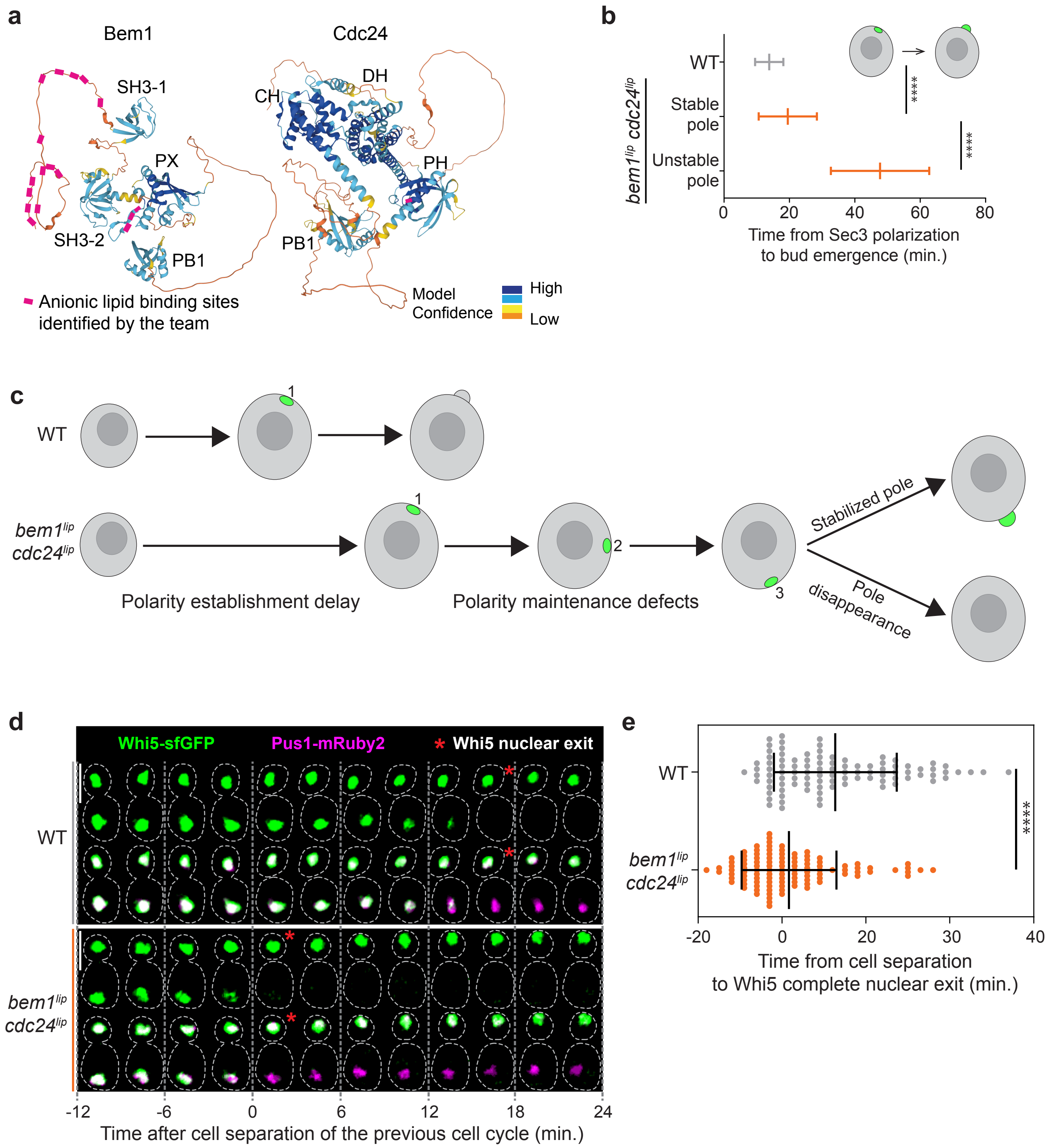

Supplementary Fig. 1

Supplementary Fig. 1. Polarity defects in the *bem1*^{lip} *cdc24*^{lip} mutant delay bud emergence and the coordination of nuclear - cytoplasmic events.

(a) Alphafold models of Bem1 (https://alphafold.ebi.ac.uk/entry/P29366) and Cdc24 (https://alphafold.ebi.ac.uk/entry/P11433) in which mutated basic residues that interact with anionic lipids are shown in pink. Model confidence values are included. **(b)** Time between Sec3-GFP polarization and bud emergence in wild type (n = 73 cells) and *bem1*^{lip} *cdc24*^{lip} cells exhibiting a stable pole (n = 61 cells) or an unstable pole (n = 18). Cells from multiple replicates have been pooled to generate the graph (N = 4 for WT; N = 8 for *bem1*^{lip} *cdc24*^{lip}). Bars correspond to mean +/- SD. Mann-Whitney (two-tailed unpaired) tests were performed (****$p < 0.0001$). **(c)** A schematic summarizing the polarity establishment and maintenance defects displayed by the *bem1*^{lip} *cdc24*^{lip} mutant. **(d)** Live imaging was performed in asynchronous WT and *bem1*^{lip} *cdc24*^{lip} cells. Images are maximum-intensity projected z-stacks. The time of cell separation in the previous cell cycle has been used as reference (time, 0). Whi5-sfGFP (green) nuclear localization was determined using a nucleoplasmic marker (Pus1-mRuby2, magenta) and its complete exit from the nucleus is highlighted by a red asterisk. Scale bars, 5 µm. **(e)** Scatter dot plot showing the time between cell separation of the previous cell cycle and Whi5 complete nuclear exit in WT (n = 98) and *bem1*^{lip} *cdc24*^{lip} cells (n = 108). Only mother cells were quantified. Bars correspond to mean +/- SD. A two-tailed unpaired t-test with Welch's correction was performed (****$p = 9.77e^{-09}$). Cells from multiple replicates were pooled to generate the graph (N = 3 for WT; N = 3 for *bem1*^{lip} *cdc24*^{lip}). Source data and exact p values are provided as a Source Data file.

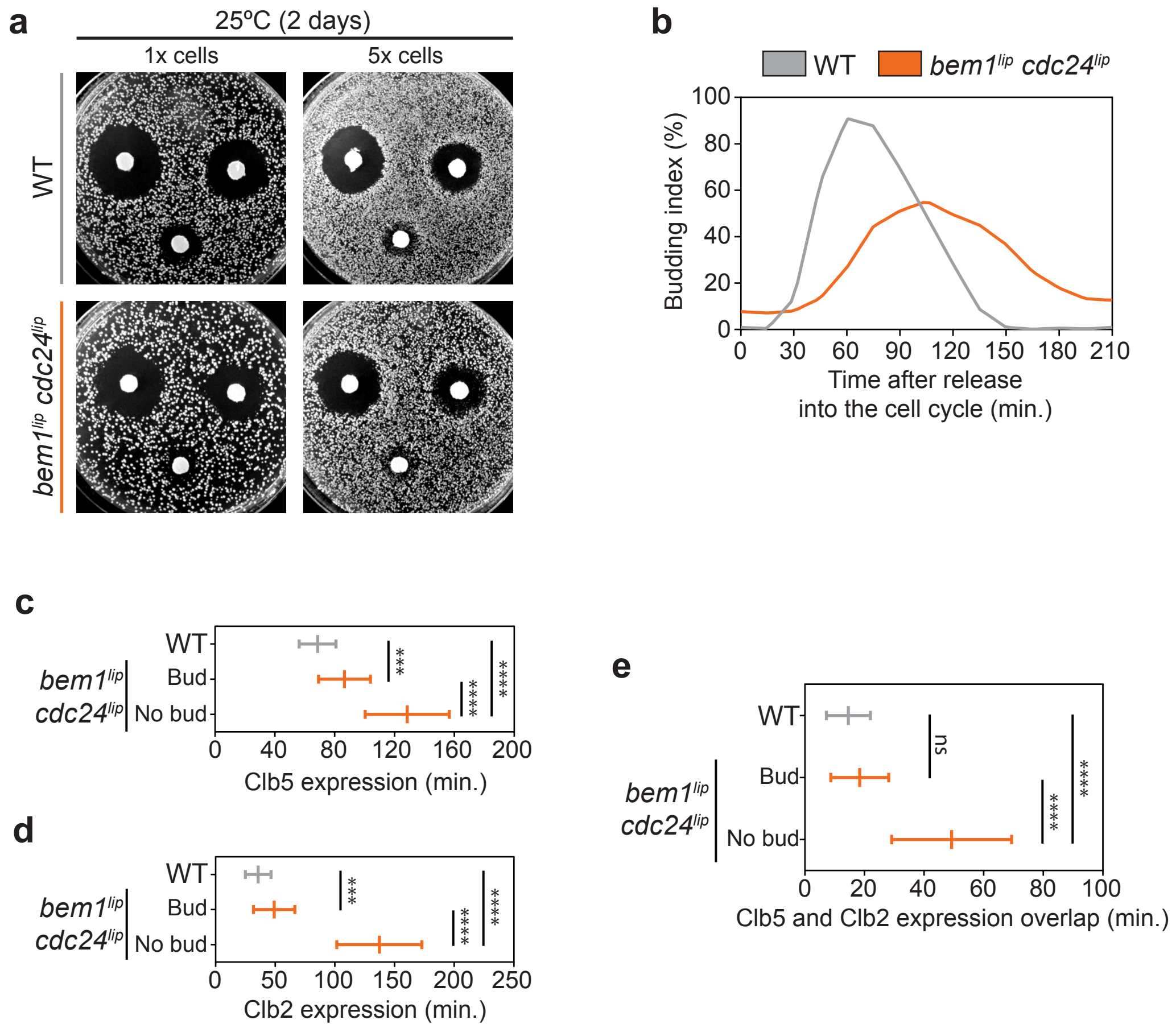

Supplementary Fig. 2

Supplementary Fig. 2. The *bem1^{lip} cdc24^{lip}* mutant responds to alpha-factor but its polarity defects impact the decisiveness of late cell cycle events and trigger Swe1^{Wee1} activation.

(a) WT and *bem1^{lip} cdc24^{lip}* cells were adjusted to the same OD_{600nm} and grown on YPD plates containing alpha-factor at 0.5 mg.ml^{-1}, 0.05 mg.ml^{-1} and 0.005 mg.ml^{-1}. Alpha-factor induces a G1 arrest that generates a halo of growth inhibition, enabling the sensitivity to G1 arrest to be assessed qualitatively after two days of growth at 25°C. Images are representative of multiple biological replicates (*N* = 3 for each condition). **(b)** Frequency of budded WT and *bem1^{lip} cdc24^{lip}* cells at the times indicated after synchronous release into the cell cycle. This data is from the time-course shown in Figs. 1f and g. **(c)** Timing of Clb5-sfGFP (S-phase) expression in WT (*n* = 35 cells), *bem1^{lip} cdc24^{lip}* cells that bud (*n* = 19 cells) and that do not bud (*n* = 27 cells). **(d)** Timing of Clb2-2xmScarletl3 (M-phase) expression in WT (*n* = 28 cells), *bem1^{lip} cdc24^{lip}* cells that bud (*n* = 18 cells) and that do not bud (*n* = 13 cells). **(e)** Timing of Clb5-sfGFP and Clb2-2xmScarletl3 expression overlap in WT (*n* = 38 cells), budded *bem1^{lip} cdc24^{lip}* cells (*n* = 19 cells) and unbudded *bem1 cdc24^{lip}* cells (*n* = 27 cells). The data from (**c, d, e**) is from the imaging shown in Fig. 1h. Bars correspond to mean +/- SD. Mann-Whitney (two-tailed unpaired) tests were performed (ns = not significant; ***p < 0.001; ****p < 0.0001). Cells from multiple replicates were pooled to generate the graphs (*N* = 4 for WT; *N* = 4 for *bem1^{lip} cdc24^{lip}*). Source data and exact *p* values are provided as a Source Data file.

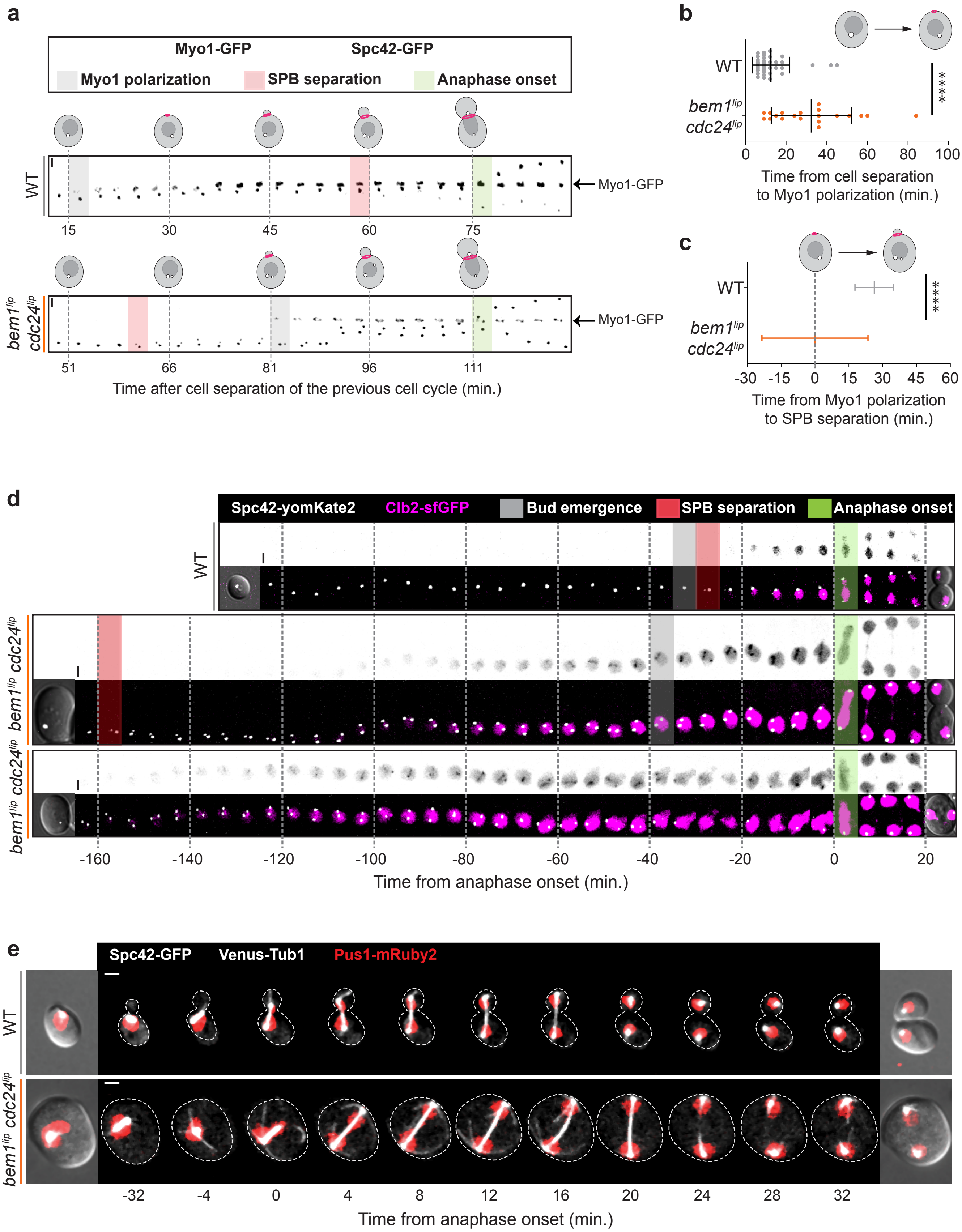

Supplementary Fig. 3

Supplementary Fig. 3. Strong polarity defects in the *bem1^lip^ cdc24^lip^* mutant result in the misordering of cell cycle events and whole genome duplication.

(a) Time-lapse images of WT and *bem1^lip^ cdc24^lip^* cells from asynchronous cultures expressing Spc42-GFP and Myo1-GFP to visualize SPB dynamics and the recruitment of the myosin-II heavy chain (Myo1) to the bud neck, respectively. Maximum-intensity projected z-stacks of inverted fluorescence images. The time of cell separation in the previous cell cycle has been used as reference (time, 0). Schematics are displayed above the fluorescence images (SPBs in white, myosin ring in pink). Note how the invariant order of Myo1 polarization (grey shading), SPB separation (red shading) then anaphase onset (green shading) observed in WT cells is not observed in the *bem1^lip^ cdc24^lip^* mutant. Scale bars, 2 µm. **(b)** Time from cell separation to Myo1 polarization in WT (*n* = 37 cells) and *bem1^lip^ cdc24^lip^* (*n* = 20 cells). **(c)** Time from Myo1 polarization to SPB separation in WT (*n* = 41 cells) and *bem1^lip^ cdc24^lip^* (*n* = 23 cells). The data from (**b, c**) is from the imaging shown in (**a**). Bars correspond to mean +/- SD. Mann-Whitney (two-tailed unpaired) tests were performed (****$p < 0.0001$). Cells from multiple replicates were pooled to generate the graphs (*N* = 5 for WT; *N* = 3 for *bem1^lip^ cdc24^lip^*). Source data and exact *p* values are provided as a Source Data file. **(d)** Time-lapse images of WT (top panel) and *bem1^lip^ cdc24^lip^* mutant cells (middle and bottom panels) from asynchronous cultures expressing Clb2-sfGFP (magenta) and Spc42-yomKate2 (white). Bud emergence, SPB separation and anaphase onset are indicated in grey, pink and green shading, respectively. In the *bem1^lip^ cdc24^lip^* cells shown in the bottom panels, SPB separation occurs 200 minutes before anaphase (not shown). Images are representative of multiple biological replicates (*N* = 3 for WT; *N* = 3 for *bem1^lip^ cdc24^lip^*). **(e)** Time-lapse images of WT (top panel) and *bem1^lip^ cdc24^lip^* mutant cells (bottom panels) from asynchronous cultures expressing Venus-Tub1 (microtubules, white), Spc42-GFP (white), and Pus1-mRuby2 (red). Images are representative of multiple biological replicates (*N* = 3 for WT; *N* = 6 for *bem1^lip^ cdc24^lip^*). The data from (**d, e**) shows images of maximum-intensity projected z-stacks and the time indicated is relative to anaphase onset (time, 0). Scale bars, 2 µm.

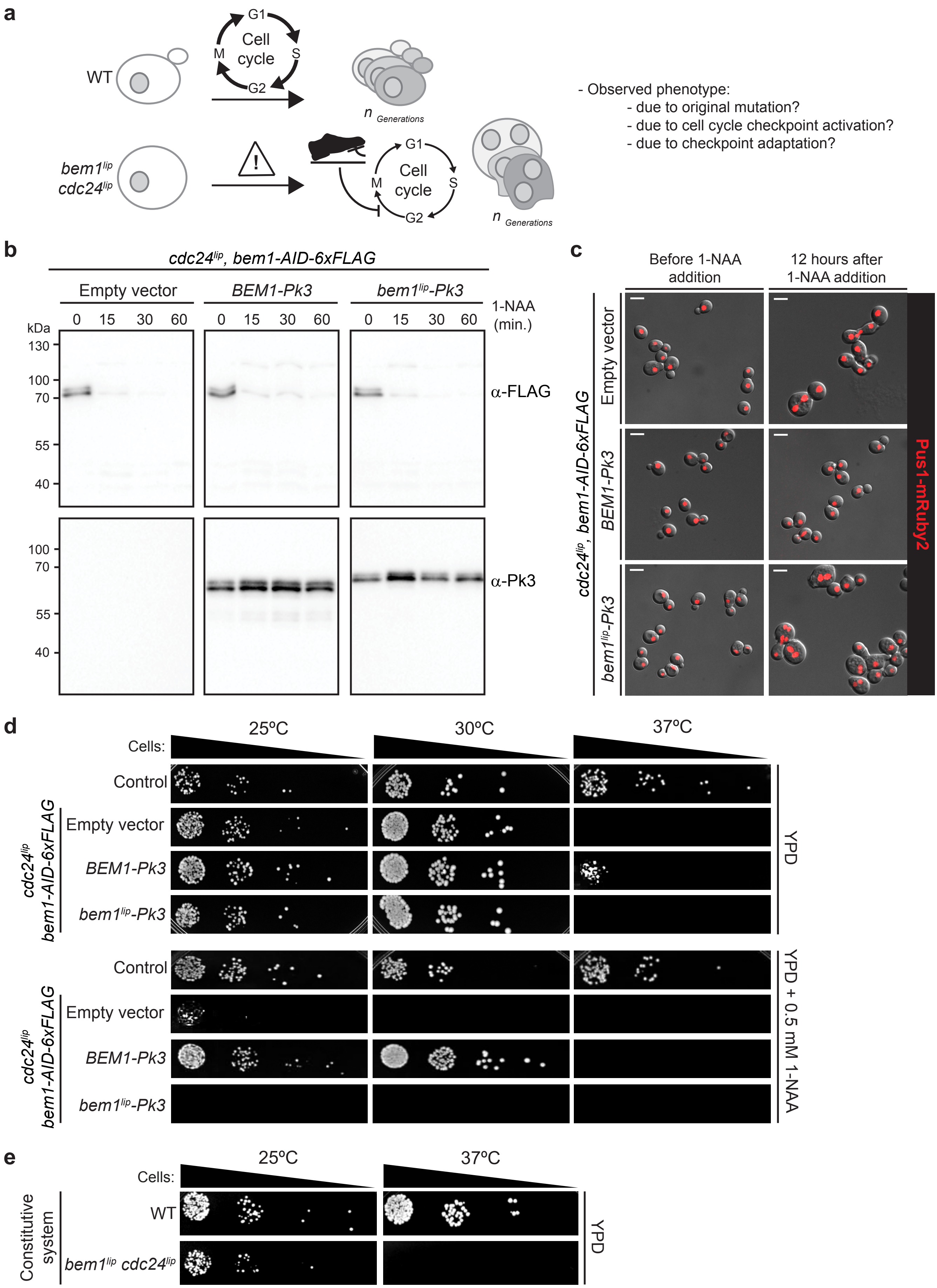

Supplementary Fig. 4

Supplementary Fig. 4. Characterization of an auxin-inducible degron (*bem1-AID*) to rapidly and conditionally ablate Bem1 function.

(a) Schematic depicting the difficulty of assigning a phenotype to mutants that activate cell cycle checkpoints. **(b)** Western blots where *BEM1-Pk3*, *bem1^{lip}-Pk3*, or cells transformed with an empty vector were treated with the synthetic auxin hormone 1-NAA for the times indicated. Endogenous bem1-AID-6xFLAG was degraded within 15 minutes, while Bem1-Pk3 or bem1^{lip}-Pk3 was expressed to comparable levels, both being expressed from the *BEM1* promoter. Note that the bem1^{lip}-Pk3 protein displays a slower electrophoretic mobility than Bem1-Pk3 by SDS-PAGE due to the charge imparted by the replacement of 14 N-terminal lysine/arginine residues with glutamate. **(c)** Cells of the indicated genotype were treated with 0.5 mM auxin for 12 hours and Pus1-mRuby2, a nucleoplasmic marker, was imaged. Scale bar, 5 µm. Cells containing an empty vector or *bem1^{lip} cdc24^{lip}* displayed morphological defects and many became multinucleate, while those transformed with WT *BEM1-Pk3* did not. **(d)** 10-fold serial dilutions of the indicated strains were spotted onto YPD plates or YPD plates supplemented with 0.5 mM 1-NAA and grown at 25°C, 30°C or 37°C for two days. When spotted onto plates in the absence of 1-NAA, all strains formed colonies at a similar rate at 25°C and 30°C. However, when cells containing an empty vector or the *bem1^{lip} cdc24^{lip}* mutant were incubated for two days in the presence of 1-NAA, they lost viability at 25°C and 30°C. **(e)** 10-fold serial dilutions of WT and *bem1^{lip} cdc24^{lip}* cells (constitutive system) were spotted onto YPD plates and grown for two days at the temperatures indicated. We interpret the more severe loss of viability at 25°C in the AID system (**d**) compared to the constitutive system (**e**) as being indicative of adaptive mechanisms responding to the loss of bem1 and cdc24 lipid binding in the constitutive system. The advantage of the AID system is that there is insufficient time for adaptation.

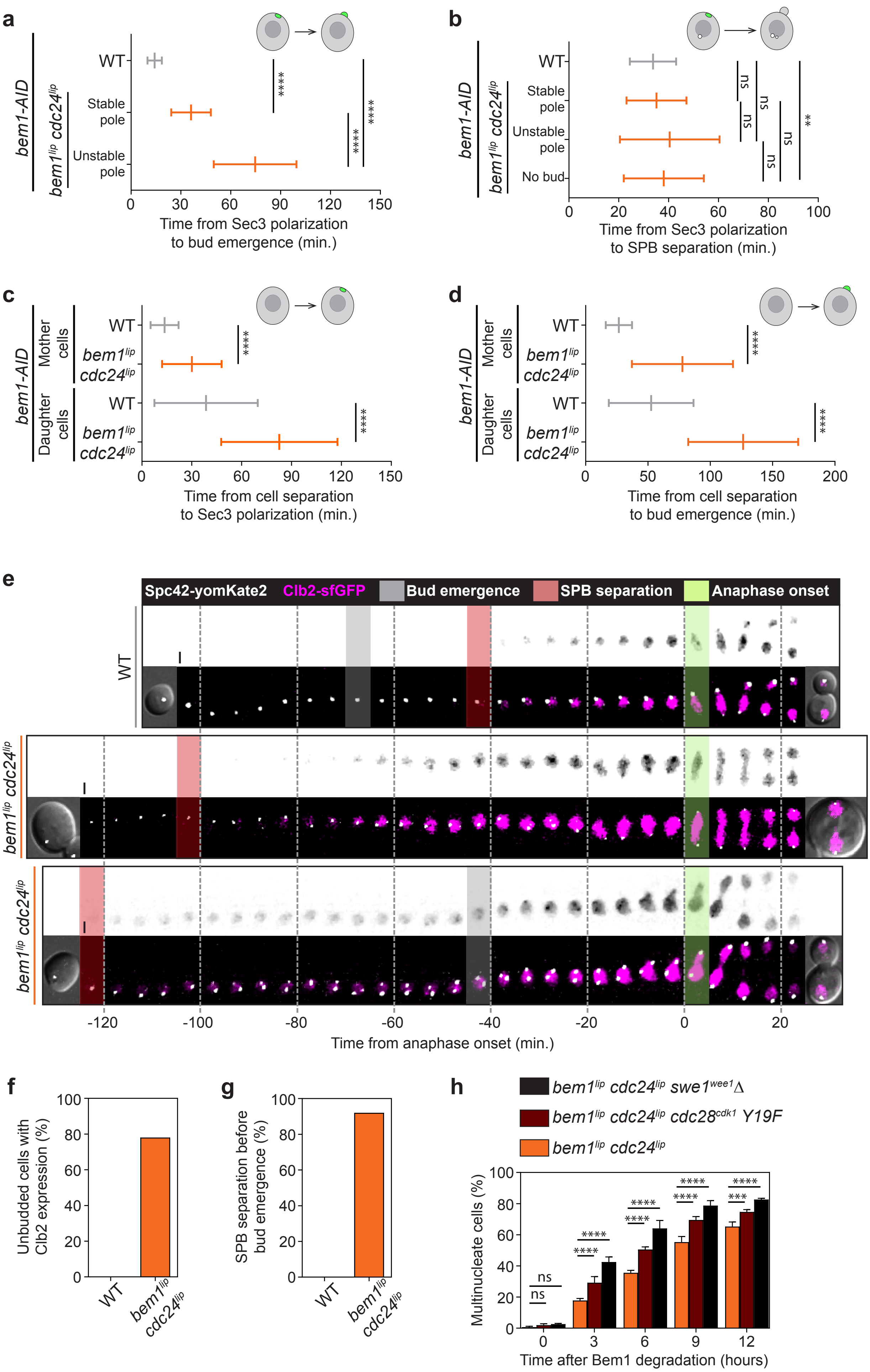

Supplementary Fig. 5

Supplementary Fig. 5. The SPB duplication cycle continues regardless of the severity of polarity defects, resulting in the appearance of multinucleate cells.

(a) Time from the first Sec3 polarization to bud emergence in WT (n = 190), *bem1^{lip} cdc24^{lip}* cells that displayed a stable pole (n = 43), or unstable pole (n = 17) until bud formation. **(b)** Time from the first Sec3 polarization event to SPB separation in WT (n = 190), *bem1^{lip} cdc24^{lip}* cells that displayed a stable pole (n = 43), or unstable pole followed by budding (n = 18), and unstable pole with no bud (n = 131). The data from (**a, b**) is from the imaging shown in Fig. 2. Bars correspond to mean +/- SD. Two-tailed unpaired *t*-tests with Welch's correction were performed (ns = not significant; **p < 0.01; ****p < 0.0001). Cells from multiple replicates were pooled to generate the graphs (N = 8 for WT; N = 7 for *bem1^{lip} cdc24^{lip}*). **(c)** Time from cell separation to Sec3 polarization in WT cells (n = 51 for mother, n = 35 for daughter) and *bem1^{lip} cdc24^{lip}* cells (n = 54 for mother, n = 41 for daughter). **(d)** Time from cell separation to bud emergence in WT (n = 51 for mother, n = 35 for daughter) and *bem1^{lip} cdc24^{lip}* cells (n = 29 for mother, n = 26 for daughter). In (**c, d**), the experiments were done with asynchronous cultures where auxin was added 30 minutes before imaging. Bars correspond to mean +/- SD. Two-tailed unpaired *t*-tests with Welch's correction were performed (****p < 0.0001). Cells from multiple replicates were pooled to generate the graphs (N = 3 for WT; N = 4 for *bem1^{lip} cdc24^{lip}*). **(e)** Time-lapse images of WT and *bem1^{lip} cdc24^{lip}* cells expressing Clb2-sfGFP (magenta) and Spc42-yomKate2 (white) from asynchronous culture where 1-NAA was added to degrade bem1-AID before imaging. Bud emergence, SPB separation and anaphase onset are indicated in grey, pink and green shading respectively. Images of maximum-intensity projected z-stacks. Time 0 corresponds to anaphase onset. Scale bars, 2 µm. **(f)** Frequency of unbudded WT and *bem1^{lip} cdc24^{lip}* cells expressing Clb2-sfGFP. **(g)** Frequency of WT and *bem1^{lip} cdc24^{lip}* cells undergoing SPB separation before bud emergence. The data from (**e**, **f**, **g**) are representative of multiple biological replicates (N = 2 for WT; N = 3 for *bem1^{lip} cdc24^{lip}*) where cells were pooled to generate the graphs (n = 30 for WT; n = 50 for *bem1^{lip} cdc24^{lip}*). **(h)** Frequency of multinucleate cells that appear after 1-NAA treatment in *bem1^{lip} cdc24^{lip}*, *bem1^{lip} cdc24^{lip} cdc28^{cdk1} Y19F* and *bem1^{lip} cdc24^{lip} swe1^{wee1}Δ* cells (n > 100 cells per time point, results of three biological replicates). Values display mean +/- SD. A two-way ANOVA was performed (ns = not significant; ** p < 0.01; *** p < 0.001; **** p < 0.0001). Source data, exact n and p values are provided as a Source Data file.

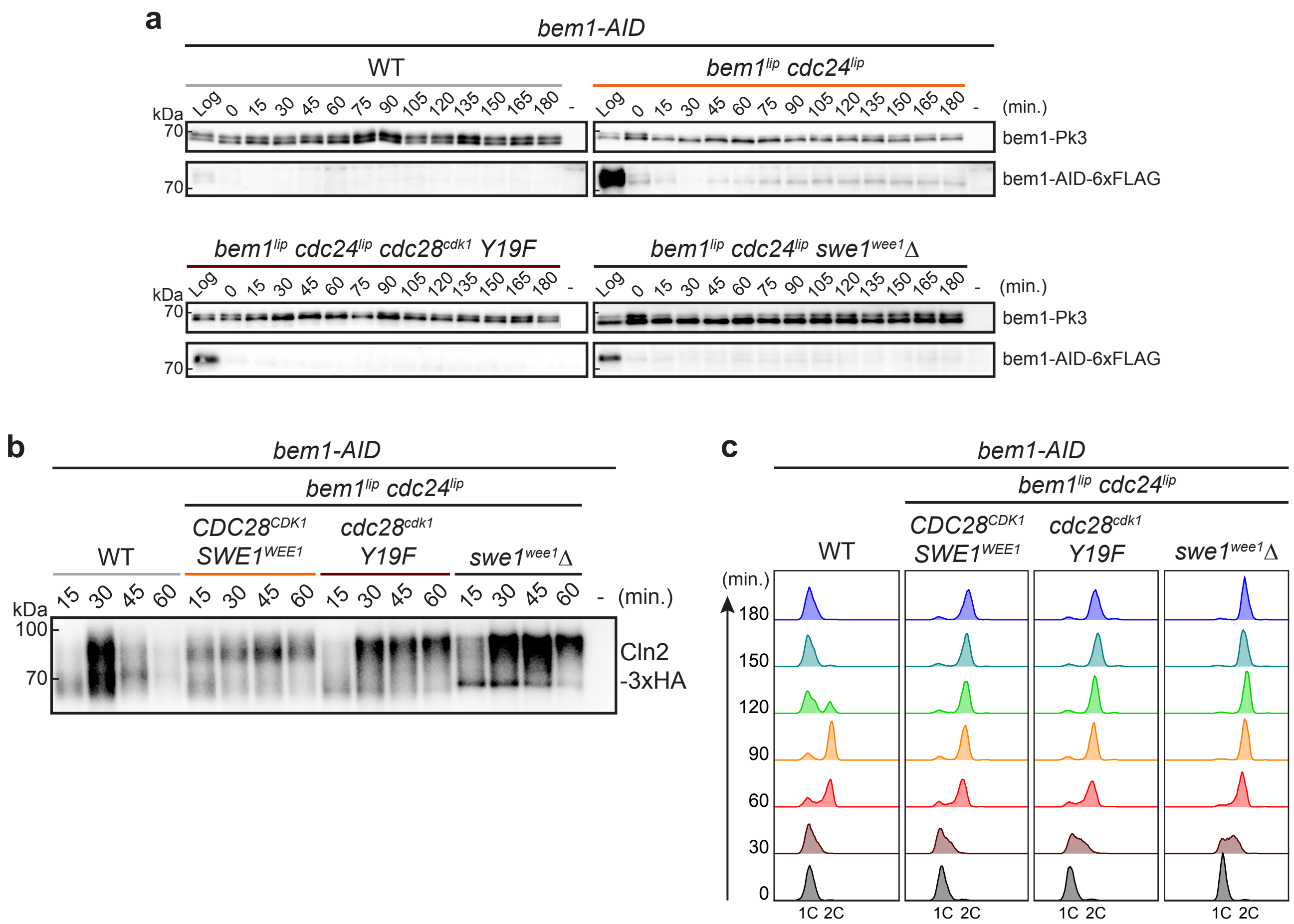

Supplementary Fig. 6

Supplementary Fig. 6. Control Western blots and flow-cytometry analysis of DNA content in *BEM1*, *bem1^{lip} cdc24^{lip}*, *bem1^{lip} cdc24^{lip} cdc28^{cdk1} Y19F* and *bem1^{lip} cdc24^{lip} swe1^{wee1}*Δ cells.

The data accompanies the cell cycle time-courses shown in Figure 3. Cells were arrested in G1, treated with auxin for the final 30 minutes of alpha-factor arrest to degrade bem1-AID, then released into the cell cycle. Samples were collected at the indicated times and processed for flow cytometry and protein analysis. **(a)** Cells of the indicated genotype were analyzed by Western blotting. **(b)** Western blots of samples used in Figure 3. Samples were run on the same gel for direct comparison. **(c)** Flow-cytometry analysis of DNA content. Time 0 corresponds to G1 arrest. Source data are provided as a Source Data file.

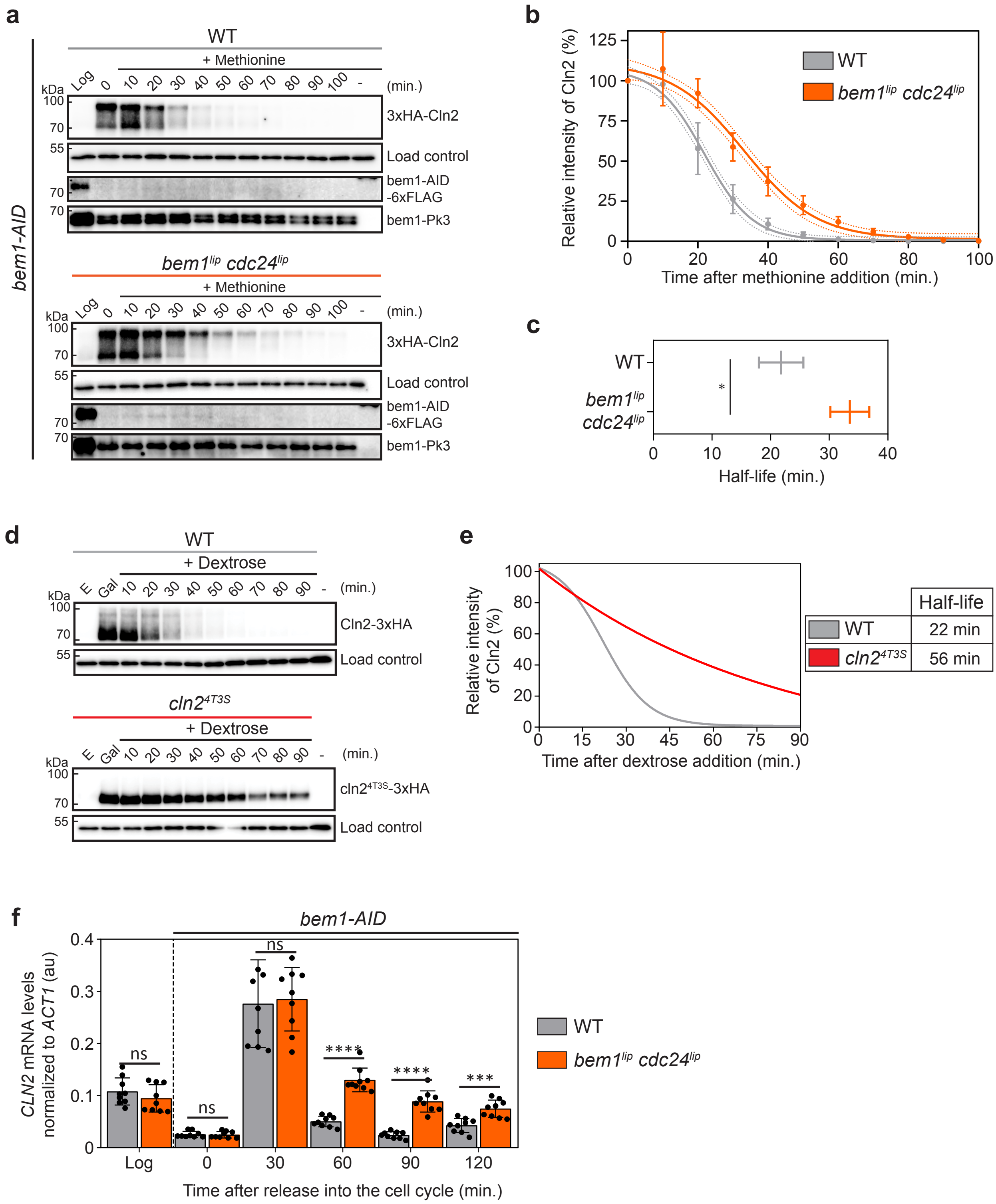

Supplementary Fig. 7

Supplementary Fig. 7. The G1 cyclin Cln2 expression is increased in the *bem1^{lip} cdc24^{lip}* mutant.

(a) 3xHA-Cln2, under control of the *MET25* promoter, was expressed for 2 hours and then repressed by addition of methionine in WT or *bem1^{lip} cdc24^{lip}* mutant cells. Samples were collected at the indicated times for western blotting. “Log”: uninduced sample grown in YPD + methionine. This experiment was repeated 5 times, with similar results. **(b)** Relative abundance of Cln2 protein was quantified from western blots. Lines correspond to nonlinear regression fitting (sigmoidal, R^2 of 0.98 for WT and 0.96 for *bem1^{lip} cdc24^{lip}*). Confidence intervals of 95 % of fits are shown (dashed lines). Dots and bars display experimental means +/- SD for each time point. **(c)** Half-life of Cln2 protein in WT (n = 4) and *bem1^{lip} cdc24^{lip}* mutant cells (n = 5) was extracted from (b). Bars correspond to mean +/- SD. Mann-Whitney test (two-tailed unpaired) was performed (*p = 0.016). **(d)** Cln2-3xHA or cln2^{4T3S}-3xHA, expressed from the *GAL1* promoter, was induced for 1 hour and then repressed via dextrose addition. Samples were collected at the indicated times for western blotting. “E”: uninduced sample grown in YEP + ethanol + glycerol. “Gal”: induced sample after 1 hour in galactose. **(e)** Relative abundance of Cln2 protein in WT (n = 2) and *cln2^{4T3S}* (n = 2) was quantified from western blots. Lines correspond to nonlinear regression fitting (sigmoidal for WT cells (R^2 of 0.99), one phase decay for *cln2^{4T3S}* cells (R^2 of 0.98)). Half-life of Cln2 protein in WT and *cln2^{4T3S}* was extracted from the fitted curve. **(f)** Histogram showing the relative abundance of *CLN2* mRNA over time for the indicated genotypes. WT and *bem1^{lip} cdc24^{lip}* cells were arrested in G1, treated with auxin for the final 30 minutes of alpha-factor arrest to degrade bem1-AID, then released into the cell cycle. Samples were collected at the indicated times and mRNAs were extracted. Multiple replicates of qPCR were performed to analyze *CLN2* mRNA levels (N = 9, displayed by points). *ACT1* mRNA levels were used for normalization. The Log sample was taken before alpha-factor treatment and bem1-AID degradation. Bars correspond to mean +/- SD. Mann-Whitney (two-tailed unpaired) tests were performed (ns = not significant; ***p < 0.001; ****p < 0.0001). Source data and exact p values are provided as a Source Data file.

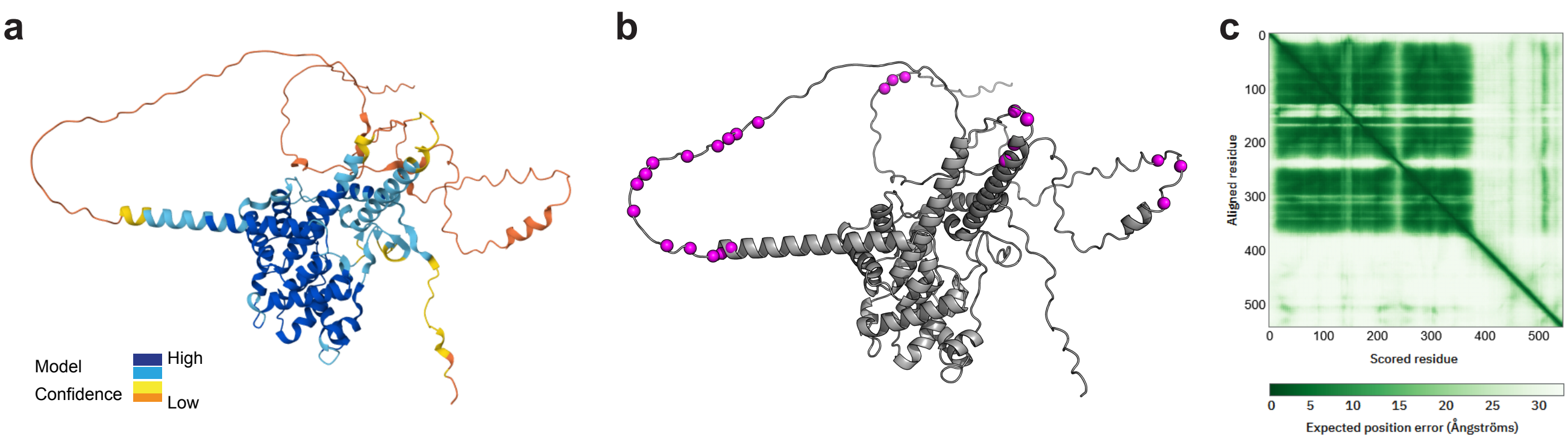

d

```
  1 MASAEPRPRM GLVINAKPDY YPIELSNAEL LSHFEMLQEY HQEISTNVIA QSCKFKPNPK
 61 LIDQQPEMNP VETRSNIITF LFELSVVTRV TNGIFFHSVR LYDRYCSKRI VLRDQAKLVV
121 ATCLWLAAKT WGGCNHIINN VVIPTGGRFY GPNPRARIPR LSELVHYCGD GQVFDESMFL
181 QMERHILDTL NWNIYEPMIN DYVLNVDENC LMQYELYENQ VTYDKQCSEK RQSQLSQDSD
241 ATVDERPYQN EEEEEEDLKL KIKLINLKKF LIDVSAWQYD LLRYELFEVS HGIFSIINQF
301 TNQDHGPFLM TPMTSESKNG EILSTLMNGI VSIPNSLMEV YKTVNGVLPF INQVKEYHLD
361 LQRKLQIASN LNISRKLTIS TPSCSFENSN STSIPSPASS SQSHTPMRNM SSLSDNSVFS
421 RNMEQSSPIT PSMYQFGQQQ SNSICGSTVS VNSLVNTNNK QRIYEQITGP NSNNATNDYI
481 DLLNLNESNK ENQNPATAHY LNGGPPKTSF INHGMFPSPT GTINSGKSSS ASSLISFGMG
541 NT QVI*
```

SP/TP Cdc28[Cdk1] consensus sites
S/T Peyran, L. *et al.*
S Breitkreutz, A. *et al.* (*Science*, 2010) PMID: 20489023
S Lanz, M. C. *et al.* (*EMBO Rep.*, 2021) PMID: 33491328

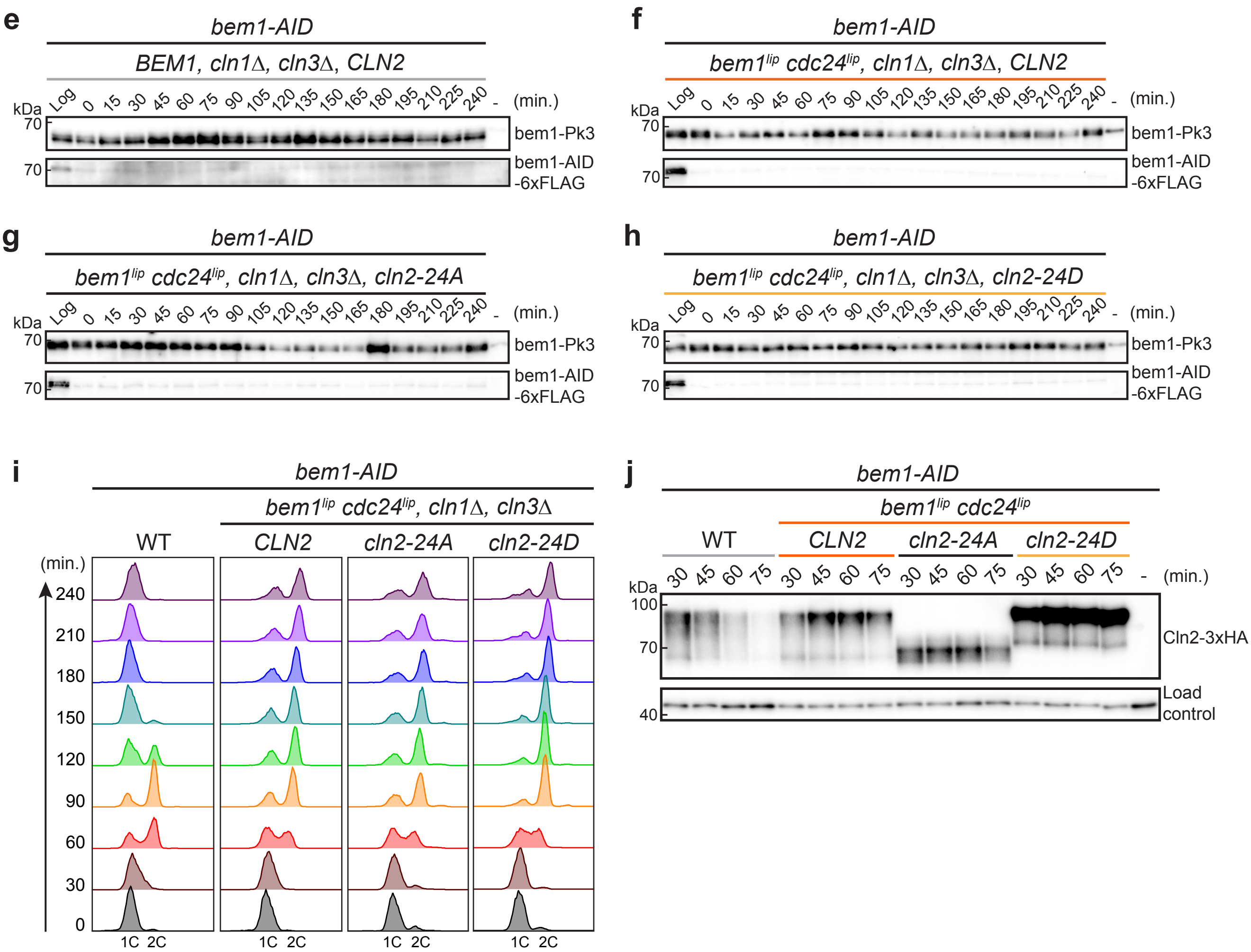

Supplementary Fig. 8

Supplementary Fig. 8. G1 cyclin Cln2 structural model, mapping of Cln2 phosphorylation sites and cell cycle time-course controls of WT, *bem1^{lip} cdc24^{lip}*, *bem1^{lip} cdc24^{lip} cln2-24A* and *bem1^{lip} cdc24^{lip} cln2-24D*.

(a) Alphafold model of Cln2 (https://alphafold.ebi.ac.uk/entry/P20438). Model confidence values are included. **(b)** Alphafold model of Cln2 displaying the position of phosphorylation sites in pink. **(c)** Model confidence values. **(d)** Amino acid sequence of Cln2 where phosphorylated residues mapped in this study and other large scale studies are highlighted in red and blue, respectively. Underlined SP/TP corresponds to the 7 Cdc28^{Cdk1} consensus sites. The data shown in (**e-j**) accompanies the cell cycle time-courses shown in Figure 4. Cells were arrested in G1, treated with auxin for the final 30 minutes of alpha-factor arrest to degrade bem1-AID, then released into the cell cycle. Samples were collected at the indicated times and processed for western blotting and flow cytometry analysis. **(e-h)** Controls western blots. **(i)** Flow-cytometric analysis of DNA content. Time 0 corresponds to G1 arrest. **(j)** Western blots of samples shown in Figure 4. The samples were run on the same gel for direct comparison. Source data are provided as a Source Data file.

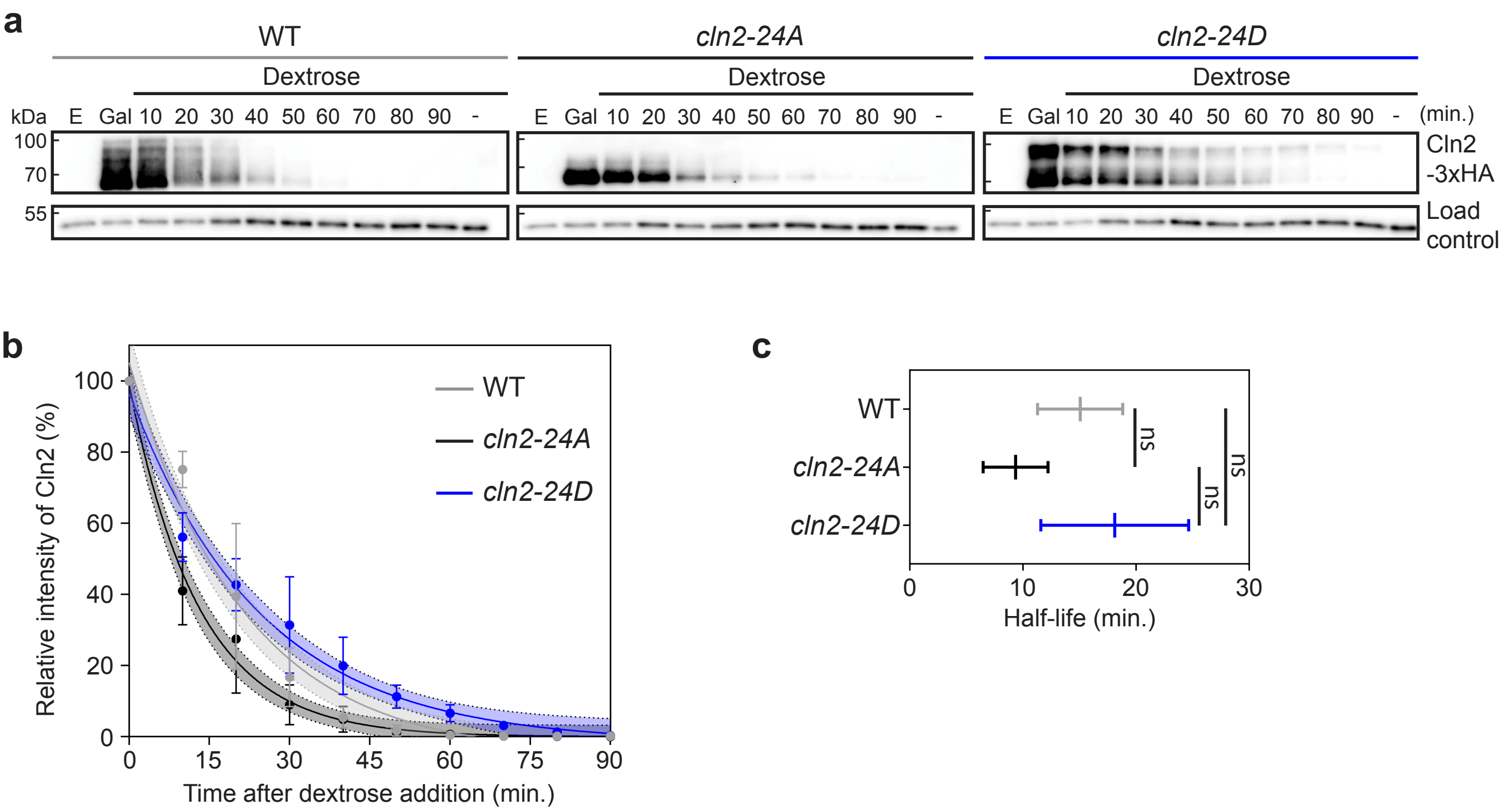

Supplementary Fig. 9

Supplementary Fig. 9. The different G1 cyclin phosphomutants display a similar rate of degradation in a *BEM1* background.

(a) Cln2-3xHA, cln2-24A-3xHA or cln2-24D-3xHA, expressed from the *GAL1* promoter, was induced for 1 hour and then repressed by dextrose addition. Samples were collected at the indicated times for western blotting. "E": uninduced sample grown in YEP + ethanol + glycerol. "Gal": induced sample after 1 hour in galactose medium. This experiment was repeated 3 times, with similar results. **(b)** Relative abundance of Cln2 protein was quantified from western blots. Lines correspond to nonlinear regression fitting (one phase decay, R^2 of 0.95 for WT; R^2 of 0.97 for *BEM1 cln2-24A*; R^2 of 0.96 for *BEM1 cln2-24D*). Confidence intervals of 95 % of fits are shown (dashed lines). Dots and bars display experimental mean +/- SD for each time point. **(c)** Half-life of Cln2 protein in WT (n = 3), *BEM1 cln2-24A* (n = 3) and *BEM1 cln2-24D* cells (n = 3) was extracted from (b). Bars correspond to mean +/- SD. Mann-Whitney tests (two-tailed unpaired) were performed (ns = not significant). Source data and exact p values are provided as a Source Data file.

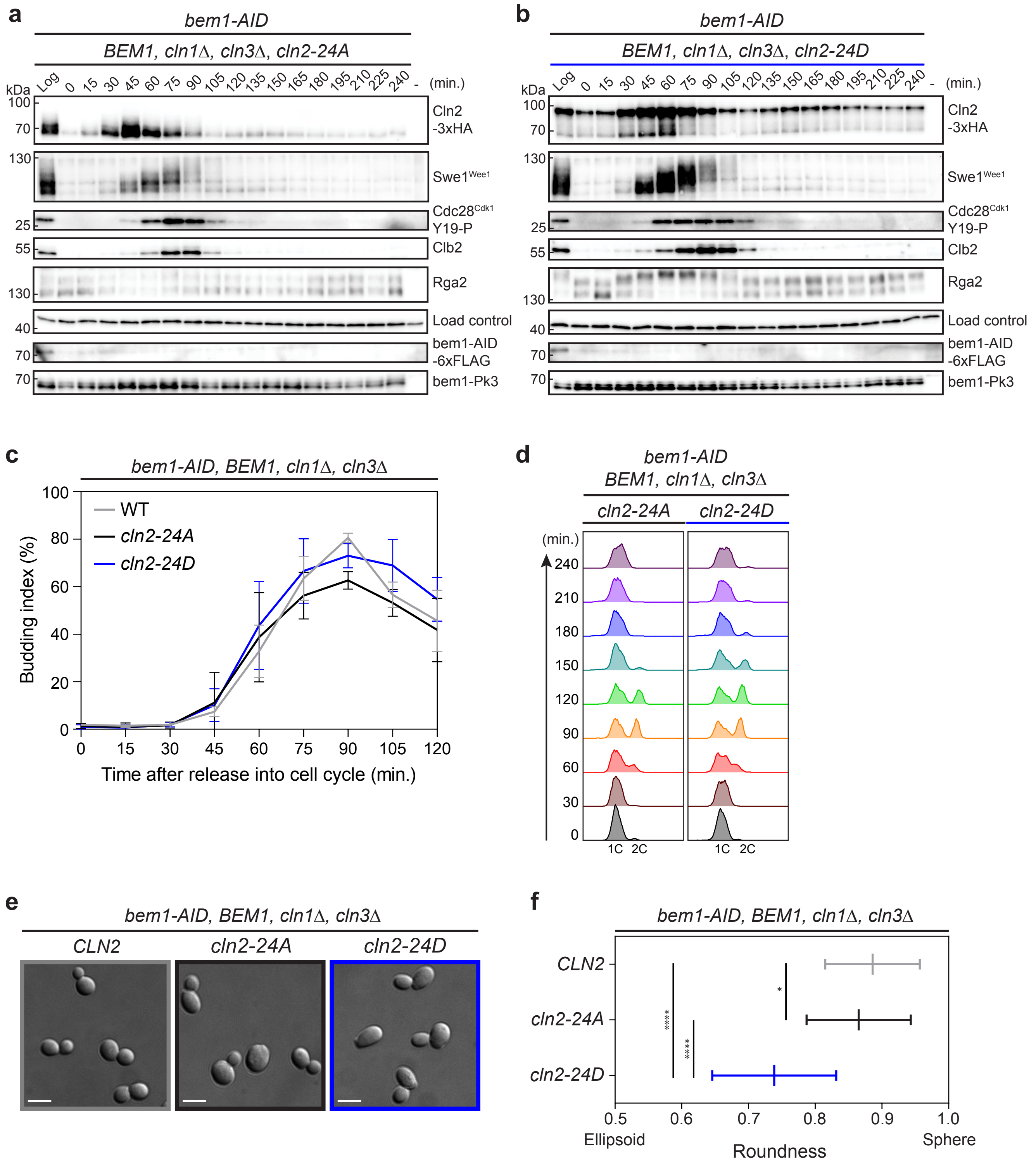

Supplementary Fig. 10

Supplementary Fig. 10. The phosphomimetic mutant *cln2-24D* in the *BEM1* background displays elevated cytoplasmic G1 cyclin-Cdc28^{Cdk1} activity, resulting in cell elongation.

BEM1 cln2-24A and *BEM1 cln2-24D* cells were arrested in G1, treated with auxin for the final 30 minutes of alpha-factor arrest to degrade bem1-AID, then released into the cell cycle. Samples were collected at the indicated times and processed for western blotting, flow cytometry and budding index. **(a)** Western blots showing cell cycle progression in *BEM1 cln2-24A* cells. **(b)** Western blots showing cell cycle progression in *BEM1 cln2-24D* cells. In (**a, b**), images are representative of multiple biological replicates ($N = 3$ for each condition). **(c)** Frequency of budded cells after release into the cell cycle. The budding index of *BEM1 CLN2* was also analyzed. Lines and bars correspond to mean +/- SD ($n > 100$ cells per time point, exact n values are provided as a Source Data file). The experiment was repeated 3 times. **(d)** Flow-cytometry analysis of DNA content. Time 0 corresponds to G1 arrest. **(e)** DIC images of *BEM1 CLN2*, *BEM1 cln2-24A* and *BEM1 cln2-24D* cells. Scale bars, 5 µm. **(f)** Quantification of the roundness of *BEM1 CLN2* (n = 109 cells), *BEM1 cln2-24A* (n = 110 cells) and *BEM1 cln2-24D* cells (n = 106 cells). The measured cells are unbudded cells from asynchronous cultures. Bars correspond to mean +/- SD. Two-tailed unpaired t-tests with Welch's correction were performed (*$p < 0.05$; ****$p < 0.0001$). Source data and exact p values are provided as a Source Data file.

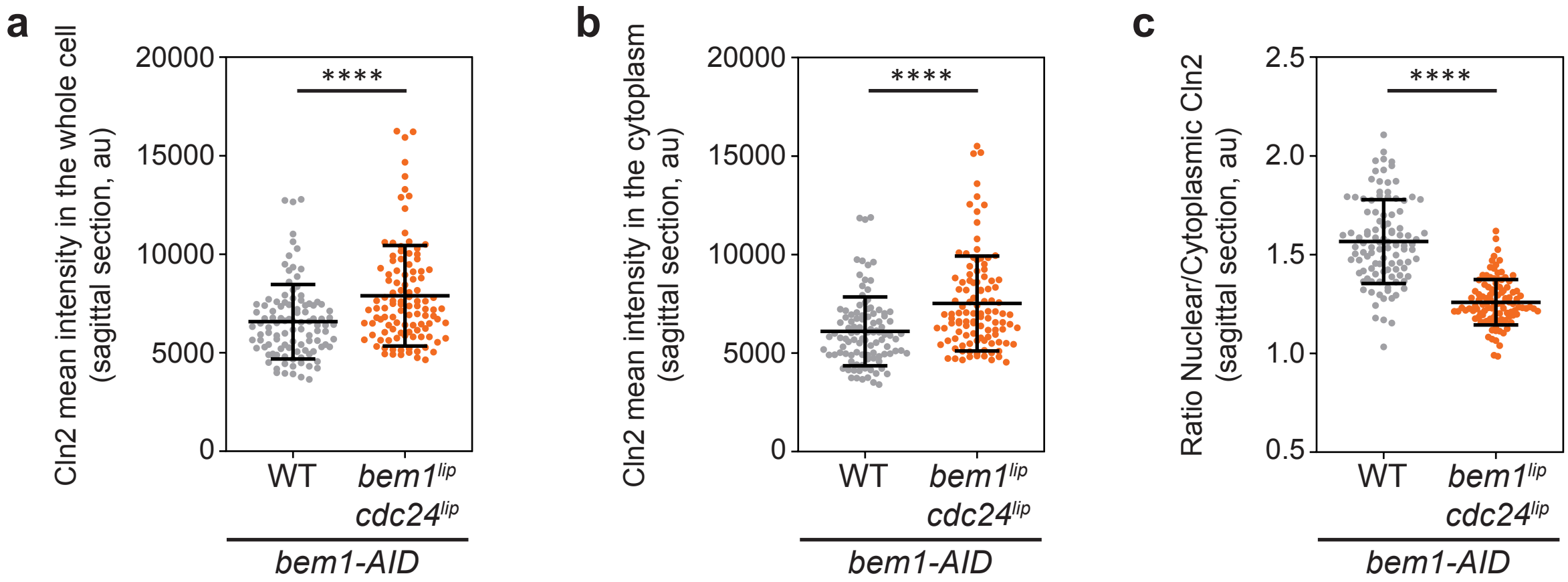

Supplementary Fig. 11

Supplementary Fig. 11. The *bem1^{lip} cdc24^{lip}* mutant displays higher levels of Cln2 in the cytoplasm.

1-NAA (0.5 mM) was added to asynchronous cultures for 5 hours to degrade bem1-AID. Cln2-sfGFP and Pus1-mRuby2 were imaged in WT (*n* = 104) or *bem1^{lip} cdc24^{lip}* mutant cells (*n* = 108). Cln2 mean fluorescence intensity was extracted from a sagittal section of single cell, from which the nuclear and cytoplasmic signal was measured using the Pus1 signal to delineate the nucleoplasm. **(a)** Scatter dot plot showing Cln2 mean fluorescence intensity in the whole cell for the indicated genotype. Lines and bars correspond to mean +/- SD. A two-tailed unpaired *t*-test with Welch's correction was performed (****p = 2.85e^{-5}). **(b)** Scatter dot plot showing Cln2 mean fluorescence intensity in the cytoplasm. Bars correspond to mean +/- SD. A two-tailed unpaired *t*-test with Welch's correction was performed (****p = 2.00e^{-6}). **(c)** Scatter dot plot showing the ratio of nuclear to cytoplasmic Cln2 mean fluorescence intensity. Bars correspond to mean +/- SD. A two-tailed unpaired *t*-test with Welch's correction was performed (****p < 1e^{-15}). Source data are provided as a Source Data file.

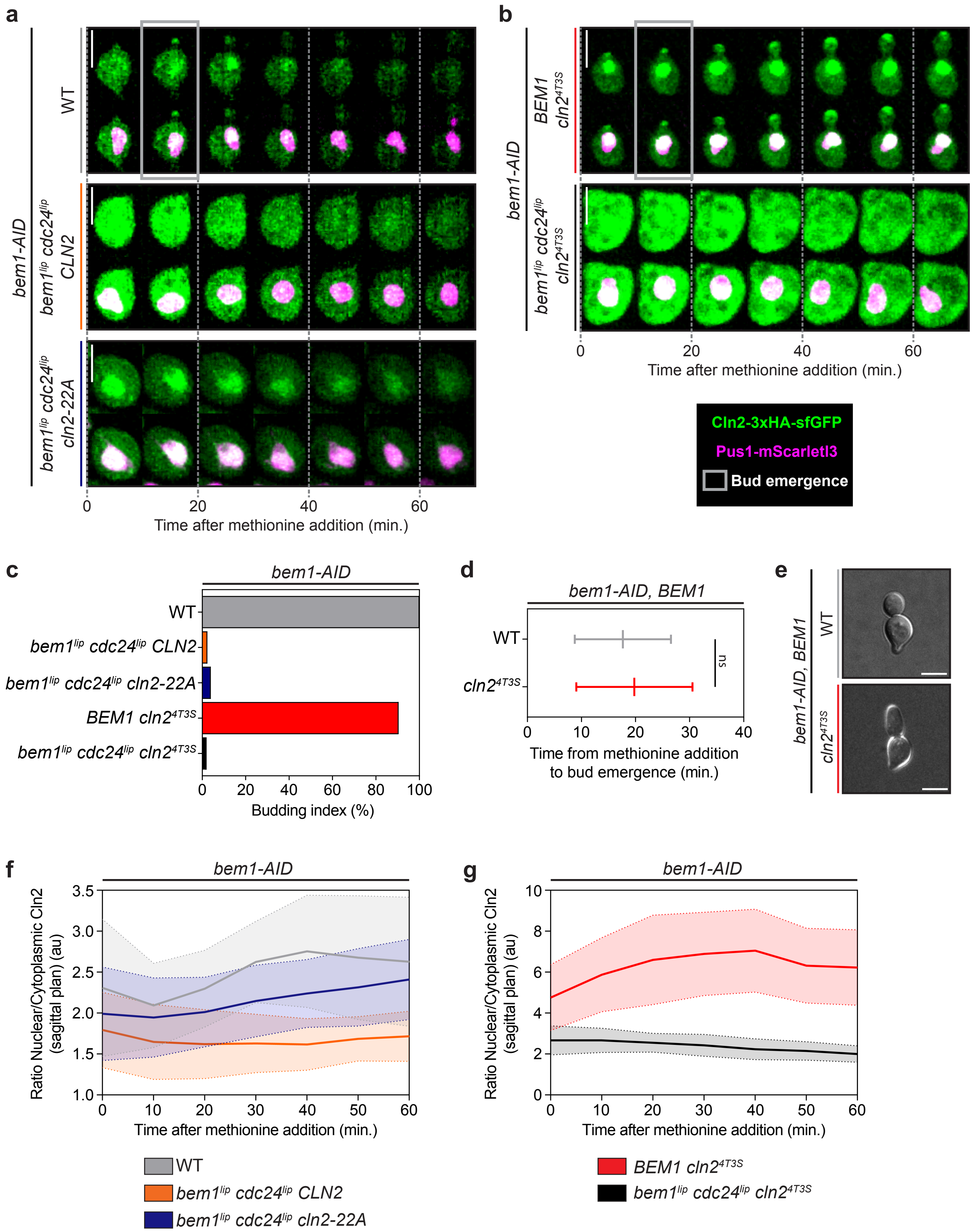

Supplementary Fig. 12

Supplementary Fig. 12. Phosphorylation of Cln2 on specific sites augments cytoplasmic G1 cyclin levels in the *bem1^{lip} cdc24^{lip}* mutant.

Cells expressing Cln2-3xHA-sfGFP, cln2-22A-3xHA-sfGFP or cln2^{4T3S}-3xHA-sfGFP under control of the *MET25* promoter were arrested in G1 and treated with auxin for the final 30 minutes of the alpha-factor arrest to degrade bem1-AID. These cells were then released into the cell cycle and grown for 70 minutes in methionine-free media to express cln2. Finally, cells were mounted on agarose pads containing methionine to repress *cln2* transcription then imaged. This experiment was repeated 3 times for each genotype, with similar results. **(a-b)** Time-lapse images of the G1 cyclin (green) and Pus1-mScarletl3 (magenta) dynamics after methionine addition (used as reference time 0) for the indicated genotype. Images are maximum-intensity projected z-stacks where bud emergence (grey rectangle) is indicated. Scale bars, 5 µm. **(c)** Frequency of budded cells after methionine addition. **(d)** Time from methionine addition to bud emergence in WT cells (n = 47) and *cln2^{4T3S}* cells that bud (n = 47). Bars correspond to mean +/- SD. A two-tailed unpaired *t*-test with Welch's correction was performed (p = 0.30). Cells from the 3 biological replicates were pooled to generate the graph. **(e)** DIC images of a WT cell and *cln2^{4T3S}* cell just before cell separation. Scale bars, 5 µm. **(f-g)** Cln2 mean fluorescence intensity was extracted from a sagittal section of the cell, from which the nuclear and cytoplasmic signal was measured using the Pus1-mScarletl3 signal to delineate the nucleoplasm. The ratio of nuclear to cytoplasmic Cln2 is displayed. Solid and dashed lines correspond to mean +/- SD. In (**c, f, g**), cells from the 3 biological replicates were pooled to generate the graphs (n = 47 cells for WT; n = 43 cells for *bem1^{lip} cdc24^{lip} CLN2*; n = 51 cells for *bem1^{lip} cdc24^{lip} cln2-22A*; n = 52 cells for *cln2^{4T3S}*; n = 51 cells for *bem1^{lip} cdc24^{lip} cln2^{4T3S}*). Source data are provided as a Source Data file.

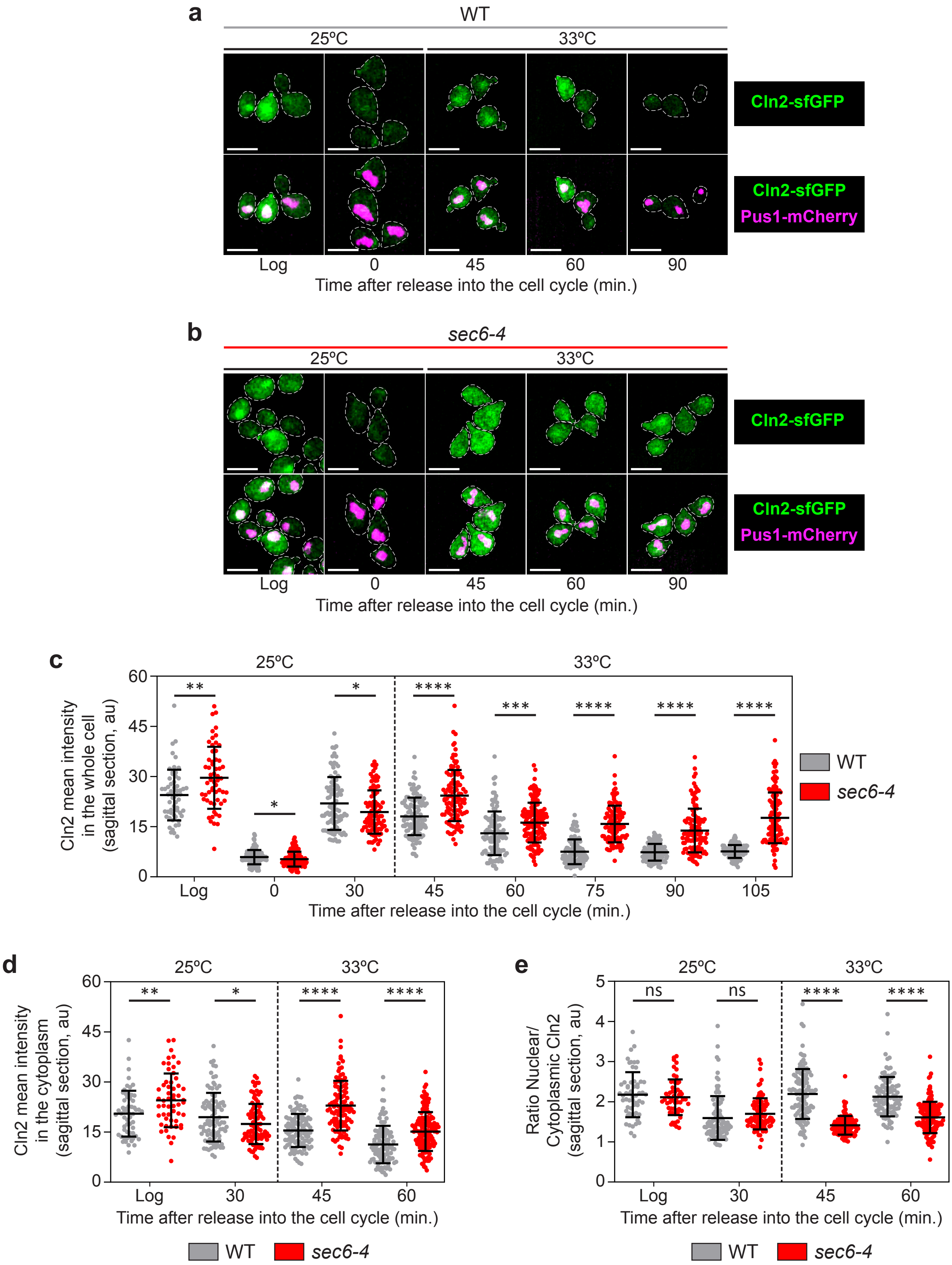

Supplementary Fig. 13

Supplementary Fig. 13. A self-correcting mechanism that augments cytoplasmic G1 cyclin levels is triggered in the *sec6-4* mutant.

WT and *sec6-4* cells were arrested in G1 with alpha-factor, then released into the cell cycle at 25°C. 30 min after the release into the cell cycle, cells were shifted to the restrictive temperature (33°C) and fixed every 15 minutes to measure Cln2 mean fluorescence intensity. Log sample was taken before alpha-factor treatment. **(a-b)** Still images of fixed WT and *sec6-4* mutant cells expressing Cln2-sfGFP (green) and Pus1-mCherry (magenta) for the indicated time. Images are maximum-intensity projected z-stacks. Scale bars, 5 µm. **(c)** Scatter dot plot showing Cln2 mean fluorescence intensity in the whole cell in WT and *sec6-4* mutant cells (n > 50 for Log time point, n > 100 cells for other time points). **(d)** Scatter dot plot showing the Cln2 mean fluorescence intensity in the cytoplasm in WT and *sec6-4* mutant cells (n > 50 for Log time point, n > 100 cells for other time points). **(e)** Scatter dot plot showing the ratio of nuclear to cytoplasmic Cln2 for WT and *sec6-4* mutant cells (n > 50 for Log time point, n > 100 cells for other time points). In (**c, d, e**), bars correspond to mean +/- SD. Two-tailed unpaired t-tests with Welch's correction were performed (ns = not significant; * $p < 0.05$; ** $p < 0.01$; *** $p < 0.001$; **** $p < 0.0001$). Source data, exact n and p values are provided as a Source Data file.

Supplementary Table 1. Plasmids used in this study.

Name	Description
pDM364 (Addgene 20754 [1])	pFA6a-*6xGLY-3xFLAG::kanMX6*
pDM443 [2]	pRS303
pDM526 [3]	pFA6a-*AID-6xFLAG::hphNT1*
pDM589 [4]	*pTIR1::LEU2*
pDM996 (derived from pFA6a-*GFP(S65T)::HIS3*) [5]	pFA6a-*GAGAGA-mRuby2::HIS3*
pDM1028 (Addgene 50632 [6])	pFA6a-*HIS3prom::Venus-Tub1+3'UTR::hphNT1*
pDM1045 [7]	pUC19-*Pk3::LEU2*
pDM1046 [8]	pFA6a-*sfGFP::kanMX6*
pDM1052 This study.	pRS303 *BEM1prom-bem1 bc-14E + px (K338M, K348A, R349A & R369A)-Pk3*
pDM1053 This study.	pRS303 *BEM1-Pk3*
pDM1058 This study.	pRS405 *CYC1promV3-PUS1-mRuby2-CYC1term*
pDM1064 (derived from pFA6a-*GFP(S65T)::HIS3*) [5]	pFA6a-*GFP(S65T)::CaURA3*
pDM1065 (derived from pFA6a-*GFP(S65T)::HIS3*) [5]	pFA6a-*GFP(S65T)::natNT2*
pDM1069 (Addgene 44878 [9])	pFA6a-*GAGAGA-yomKate2::CaURA3*
pDM1085 (subcloned from pMT104) [10]	pRS406 *CLN2-3xHA*
pDM1087 This study.	pRS406 *cln2-24A-3xHA* (*S233A, S236A, S239A, T242A, T378A, S380A, T381A, S383A, S385A, S389A, S391A, T392A, S393A, S396A, S399A, S400A, S401A, S403A, T468A, S472A, T476A, S528A, S529A, S530A)*
pDM1090 This study.	pRS406 *cln2-24D-3xHA (S233D, S236D, S239D, T242D, T378D, S380D, T381D, S383D, S385D, S389D, S391D, T392D, S393D, S396D, S399D, S400D, S401D, S403D, T468D, S472D, T476D, S528D, S529D, S530D)*
pDM1091 This study.	pUC57 *CDC28prom-cdc28 Y19F-CDC28term::natNT2*
pDM1092 This study.	pUC57 *CDC28prom-cdc28 Y19F-CDC28term::kanMX6*
pDM1131 (derived from pFA6a-*GFP(S65T)::HIS3*) [5]	pFA6a-*mScarletI3::CaURA3*
pDM1133 (derived from pFA6a-*GFP(S65T)::HIS3*) [5]	pFA6a-*mScarletI3::HIS3*
pDM1140 (derived from pFA6a-*GFP(S65T)::HIS3*) [5]	pFA6a-*mScarletI3-mScarletI3::HIS3*
pDM1149 (Addgene 74636 [11])	pFA6a-*mCherry::natNT2*
pDM1160 This study.	pUCIDT *cln2-4T3S (T311A, T381A, S396A, T405A, S427A, T430A, S518A)-3xHA-sfGFP-CLN2term::kanMX6*
pDM1161 This study.	pUCIDT *cln2-22A (S233A, S236A, S239A, T242A, T378A, S380A, S383A, S385A, S389A, S391A, T392A, S393A, S399A, S400A, S401A, S403A, T468A, S472A, T476A, S528A, S529A, S530A)-3xHA-sfGFP-CLN2term::kanMX6*
pDM1164 [12]	pUCIDT *CLN2-sfGFP-NES (AAALALKLAGLNI)-CLN2term::kanMX6*
pYM21 [13]	pYM-*9MYC::natNT2*
pYM27 [13]	pYM-*eGFP::kanMX6*
pYM-N35 [13]	pYM-*natNT2::MET25prom*
pYM-N36 [13]	pYM-*natNT2::MET25prom-3xHA*

pFA6a-*natNT2* [13]	pFA6a-*natNT2*
[5]	pFA6a-*GFP(S65T)::kanMX6*
[5]	pFA6a-*GFP(S65T)::HIS3*
[5]	pFA6a-*3xHA::kanMX6*
[5]	pFA6a-*kanMX6::GAL1prom*

Supplementary Table 2. Yeast strains used in this study.

DMY	Genotype
255	*MATa, his3Δ11, leu2Δ3,112, trp1Δ1, ura3Δ52, ade2Δ1, can1Δ100, GAL+, bar1, rga2Δ::kanMX6*
552	*MATa, his3Δ11, leu2Δ3,112, trp1Δ1, ura3Δ52, ade2Δ1, can1Δ100, GAL+, bar1, CLN2-3xHA::LEU2*
808	*MATa, his3Δ11, leu2Δ3,112, trp1Δ1, ura3Δ52, ade2Δ1, bar1, cln2Δ::HIS3, LEU2::GAL1prom-CLN2-3xHA*
809	*MATa, his3Δ11, leu2Δ3,112, trp1Δ1, ura3Δ52, ade2Δ1, bar1, cln2Δ::HIS3, LEU2::GAL1prom-*$cln2^{4T3S}$*-3xHA*
996	*MATa, his3Δ11, leu2Δ3, trp1Δ1, ura3Δ52, ade2Δ1, can1Δ100, GAL+,*<br>*HA-cdc28-T18A-Y19F:URA3*
1054	*MATa, his3Δ11, leu2Δ3,112, trp1Δ1, ura3Δ52, ade2Δ1, can1Δ100, GAL+, bar1, sec6-4::HIS3, CLN2-3xHA::LEU2*
2105	*MATa, his3Δ1, leu2Δ0, met15Δ0, ura3Δ0*
2524	*MATa, his3Δ1, leu2Δ0, met15Δ0, ura3Δ0, cdc24 K513A-3xHA::URA3, bem1 bc-14E + px (K338M, K348A, R349A & R369A)*
2578	*MATa, his3Δ1, leu2Δ0, met15Δ0, ura3Δ0, PUS1-GFP::HIS3*
2591	*MATa, his3Δ1, leu2Δ0, met15Δ0, ura3Δ0, cdc24 K513A-3xHA::URA3, bem1 bc-14E + px (K338M, K348A, R349A, R369A), PUS1-GFP::HIS3*
2718	*MATa, his3Δ1, leu2Δ0, met15Δ0, ura3Δ0, swe1Δ::kanMX6*
2722	*MATa, his3Δ1, leu2Δ0, met15Δ0, ura3Δ0, clb2Δ::kanMX6*
2803	*MATa, his3Δ1, leu2Δ0, met15Δ0, ura3Δ0, PUS1-mRuby2::HIS3, WHI5-sfGFP::kanMX6*
2806	*MATa, his3Δ1, leu2Δ0, met15Δ0, ura3Δ0, cdc24 K513A-3xHA::URA3, bem1 bc-14E+px (K338M, K348A, R349A, R369A), PUS1-mRuby2::HIS3, WHI5-sfGFP::kanMX6*
2910	*MATa, his3Δ1, leu2Δ0, met15Δ0, ura3Δ0, SIC1-3xFLAG::kanMX6, CLB5-TAP::HIS3, CLN2-Pk3::LEU2*
2912	*MATa, his3Δ1, leu2Δ0, met15Δ0, ura3Δ0, cdc24 K513A-3xHA::URA3, bem1 bc-14E + px (K338M, K348A, R349A & R369A), SIC1-3xFLAG::kanMX6, CLB5-TAP::HIS3, CLN2-Pk3::LEU2*
2957	*MATa, his3Δ1, leu2Δ0, met15Δ0, ura3Δ0, cdc24 K513A-3xHA::URA3, leu2::LEU2::TIR1, bem1-AID-6xFLAG::hphNT1, his3::HIS3*
2958	*MATa, his3Δ1, leu2Δ0, met15Δ0, ura3Δ0, cdc24 K513A-3xHA::URA3, leu2::LEU2::TIR1, bem1-AID-6xFLAG::hphNT1, his3::BEM1-Pk3::HIS3*
2960	*MATa, his3Δ1, leu2Δ0, met15Δ0, ura3Δ0, cdc24 K513A-3xHA::URA3, leu2::LEU2::TIR1, bem1-AID-6xFLAG::hphNT1, his3::BEM1prom-bem1 bc-14E + px (K338M, K348A, R349A & R369A)-Pk3::HIS3*
2962	*MATa, his3Δ1, leu2Δ0, met15Δ0, ura3Δ0, cdc24 K513A-3xHA::URA3, leu2::LEU2::TIR1, bem1-AID-6xFLAG::hphNT1, PUS1-mRuby2::kanMX6, his::HIS3*
2963	*MATa, his3Δ1, leu2Δ0, met15Δ0, ura3Δ0, cdc24 K513A-3xHA::URA3, leu2::LEU2::TIR1, bem1-AID-6xFLAG::hphNT1, PUS1-mRuby2::kanMX6, his::BEM1-Pk3::HIS3*
2966	*MATa, his3Δ1, leu2Δ0, met15Δ0, ura3Δ0, cdc24 K513A-3xHA::URA3, leu2::LEU2::TIR1, bem1-AID-6xFLAG::hphNT1, PUS1-mRuby2::kanMX6, his::BEM1prom-bem1 bc-14E + px (K338M, K348A, R349A & R369A)-Pk3::HIS3*
2978	*MATa, his3Δ1, leu2Δ0, met15Δ0, ura3Δ0, HIS3prom::Venus-TUB1::hphNT1, leu2::LEU2::CYC1promV3-PUS1-mRuby2-CYC1term, SPC42-eGFP::kanMX6*
2980	*MATa, his3Δ1, leu2Δ0, met15Δ0, ura3Δ0, cdc24 K513A-3xHA::URA3, bem1 bc-14E + px (K338M, K348A, R349A & R369A), HIS3prom::Venus-TUB1::hphNT1, leu2::LEU2::CYC1promV3-PUS1-mRuby2-CYC1term, SPC42-eGFP::kanMX6*
2985	*MATa, his3Δ1, leu2Δ0, met15Δ0, ura3Δ0, leu2::LEU2::CYC1promV3-PUS1-mRuby2-CYC1term, MYO1-GFP::HIS3, SPC42-eGFP::kanMX6,*
2986	*MATa, his3Δ1, leu2Δ0, met15Δ0, ura3Δ0, cdc24 K513A-3xHA::URA3, bem1 bc-14E + px (K338M, K348A, R349A & R369A), leu2::LEU2::CYC1promV3-PUS1-mRuby2-CYC1term, MYO1-GFP::HIS3, SPC42-eGFP::kanMX6*

2996	*MATa, his3Δ1, leu2Δ0, met15Δ0, ura3Δ0, cdc24 K513A, leu2::LEU2::TIR1, bem1-AID-6xFLAG::hphNT1, his::BEM1-Pk3::HIS3*
2998	*MATa, his3Δ1, leu2Δ0, met15Δ0, ura3Δ0, cdc24 K513A, leu2::LEU2::TIR1, bem1-AID-6xFLAG::hphNT1, his3::BEM1prom-bem1 bc-14E + px (K338M, K348A, R349A & R369A)-Pk3::HIS3*
3000	*MATa, his3Δ1, leu2Δ0, met15Δ0, ura3Δ0, CLB2-sfGFP::kanMX6, SPC42-yomKate2::CaURA3*
3005	*MATa, his3Δ1, leu2Δ0, met15Δ0, ura3Δ0, cdc24 K513A-3xHA::URA3, bem1 bc-14E + px (K338M, K348A, R349A & R369A), CLB2-sfGFP::kanMX6, SPC42-yomKate2::CaURA3*
3042	*MATa, his3Δ1, leu2Δ0, met15Δ0, ura3Δ0, SEC3-GFP::kanMX6*
3044	*MATa, his3Δ1, leu2Δ0, met15Δ0, ura3Δ0, cdc24 K513A-3xHA::URA3, bem1 bc-14E + px (K338M, K348A, R349A & R369A), SEC3-GFP::kanMX6*
3071	*MATa, his3Δ1, leu2Δ0, met15Δ0, ura3Δ0, cdc24 K513A, leu2::LEU2::TIR1, bem1-AID-6xFLAG::hphNT1, his3::BEM1prom-bem1 bc-14E + px (K338M, K348A, R349A & R369A)-Pk3::HIS3, CLN2-3HA::kanMX6*
3081	*MATa, his3Δ1, leu2Δ0, met15Δ0, ura3Δ0, cdc24 K513A, leu2::LEU2::TIR1, bem1-AID-6xFLAG::hphNT1, his::BEM1-Pk3::HIS3, CLN2-3HA::kanMX6, LTE1-9MYC::natNT2*
3083	*MATa, his3Δ1, leu2Δ0, met15Δ0, ura3Δ0, cdc24 K513A, leu2::LEU2::TIR1, bem1-AID-6xFLAG::hphNT1, his3::BEM1prom-bem1 bc-14E + px (K338M, K348A, R349A & R369A)-Pk3::HIS3, CLN2-3HA::kanMX6, LTE1-9MYC::natNT2*
3145	*MATa, his3Δ1, leu2Δ0, met15Δ0, ura3Δ0, cdc24 K513A-3xHA::URA3, bem1 bc-14E + px (K338M, K348A, R349A & R369A), HIS3prom::Venus-TUB1::hphNT1, leu2::LEU2::CYC1promV3-PUS1-mRuby2-CYC1term, SEC3-GFP::kanMX6*
3176	*MATa, his3Δ1, leu2Δ0, met15Δ0, ura3Δ0, cdc24 K513A, leu2::LEU2::TIR1, bem1-AID-6xFLAG::hphNT1, his3::BEM1prom-bem1 bc-14E + px (K338M, K348A, R349A & R369A)-Pk3::HIS3, URA3::CLN2-3xHA, cln1Δ::kanMX6, cln3Δ::natNT2*
3178	*MATa, his3Δ1, leu2Δ0, met15Δ0, ura3Δ0, cdc24 K513A, leu2::LEU2::TIR1, bem1-AID-6xFLAG::hphNT1, his3::BEM1prom-bem1 bc-14E + px (K338M, K348A, R349A & R369A)-Pk3::HIS3, URA3::cln2-24A-3xHA (S233A, S236A, S239A, T242A, T378A, S380A, T381A, S383A, S385A, S389A, S391A, T392A, S393A, S396A, S399A, S400A, S401A, S403A, T468A, S472A, T476A, S528A, S529A, S530A), cln1Δ::kanMX6, cln3Δ::natNT2*
3180	*MATa, his3Δ1, leu2Δ0, met15Δ0, ura3Δ0, cdc24 K513A, leu2::LEU2::TIR1, bem1-AID-6xFLAG::hphNT1, his3::BEM1prom-bem1 bc-14E + px (K338M, K348A, R349A & R369A)-Pk3::HIS3, URA3::cln2-24D-3xHA (S233D, S236D, S239D, T242D, T378D, S380D, T381D, S383D, S385D, S389D, S391D, T392D, S393D, S396D, S399D, S400D, S401D, S403D, T468D, S472D, T476D, S528D, S529D, S530D), cln1Δ::kanMX6, cln3Δ::natNT2*
3185	*MATa, his3Δ1, leu2Δ0, met15Δ0, ura3Δ0, HIS3prom::Venus-TUB1::hphNT1, leu2::LEU2::CYC1promV3-PUS1-mRuby2-CYC1term, SEC3-GFP::kanMX6*
3202	*MATa, his3Δ1, leu2Δ0, met15Δ0, ura3Δ0, cdc24 K513A, leu2::LEU2::TIR1, bem1-AID-6xFLAG::hphNT1, his::BEM1-Pk3::HIS3, URA3::CLN2-3xHA, cln1Δ::kanMX6, cln3Δ::natNT2*
3204	*MATa, his3Δ1, leu2Δ0, met15Δ0, ura3Δ0, cdc24 K513A, leu2::LEU2::TIR1, bem1-AID-6xFLAG::hphNT1, his::BEM1-Pk3::HIS3, URA3::cln2-24A-3xHA (S233A, S236A, S239A, T242A, T378A, S380A, T381A, S383A, S385A, S389A, S391A, T392A, S393A, S396A, S399A, S400A, S401A, S403A, T468A, S472A, T476A, S528A, S529A, S530A), cln1Δ::kanMX6, cln3Δ::natNT2*
3206	*MATa, his3Δ, leu2Δ0, met15Δ0, ura3Δ0, cdc24 K513A, leu2::LEU2::TIR1, bem1-AID-6xFLAG::hphNT1, his::BEM1-Pk3::HIS3, SEC3-GFP::KanMX6, SPC42-yomKate2::CaURA3*
3207	*MATa, his3Δ1, leu2Δ0, met15Δ0, ura3Δ0, cdc24 K513A, leu2::LEU2::TIR1, bem1-AID-6xFLAG::hphNT1, his3::BEM1prom-bem1 bc-14E + px (K338M, K348A, R349A & R369A)-Pk3::HIS3, SEC3-GFP::KanMX6, SPC42-yomKate2::CaURA3*
3212	*MATa, his3Δ1, leu2Δ0, met15Δ0, ura3Δ0, cdc24 K513A, leu2::LEU2::TIR1, bem1-AID-6xFLAG::hphNT1, his::BEM1-Pk3::HIS3, CLB2-sfGFP::kanMX6, SPC42-yomKate2::CaURA3*
3214	*MATa, his3Δ1, leu2Δ0, met15Δ0, ura3Δ0, cdc24 K513A, leu2::LEU2::TIR1, bem1-AID-6xFLAG::hphNT1, his3::BEM1prom-bem1 bc-14E + px (K338M, K348A, R349A & R369A)-Pk3::HIS3,*

	CLB2-sfGFP::kanMX6, SPC42-yomKate2::CaURA3
3221	*MATa, his3Δ1, leu2Δ0, met15Δ0, ura3Δ0, cdc24 K513A, leu2::LEU2::TIR1, bem1-AID-6xFLAG::hphNT1, his::BEM1-Pk3::HIS3, URA3::cln2-24D-3xHA (S233D, S236D, S239D, T242D, T378D, S380D, T381D, S383D, S385D, S389D, S391D, T392D, S393D, S396D, S399D, S400D, S401D, S403D, T468D, S472D, T476D, S528D, S529D, S530D), cln1Δ::kanMX6, cln3Δ::natNT2*
3230	*MATa, his3Δ1, leu2Δ0, met15Δ0, ura3Δ0, cdc24 K513A, leu2::LEU2::TIR1, bem1-AID-6xFLAG::hphNT1, his3::BEM1prom-bem1 bc-14E + px (K338M, K348A, R349A & R369A)-Pk3::HIS3, PUS1-GFP::CaURA3*
3234	*MATa, his3Δ1, leu2Δ0, met15Δ0, ura3Δ0, cdc24 K513A, leu2::LEU2::TIR1, bem1-AID-6xFLAG::hphNT1, his3::BEM1prom-bem1 bc-14E + px (K338M, K348A, R349A & R369A)-Pk3::HIS3, swe1Δ::kanMX6, PUS1-GFP::CaURA3*
3326	*MATa, his3Δ1, leu2Δ0, met15Δ0, ura3Δ0, cdc24 K513A, leu2::LEU2::TIR1, bem1-AID-6xFLAG::hphNT1, his3::BEM1prom-bem1 bc-14E + px (K338M, K348A, R349A & R369A)-Pk3::HIS3, CDC28prom-cdc28 Y19F-CDC28term::kanMX6, PUS1-GFP::CaURA3*
3333	*MATa, his3Δ1, leu2Δ0, met15Δ0, ura3Δ0, cdc24 K513A, leu2::LEU2::TIR1, bem1-AID-6xFLAG::hphNT1, his3::BEM1prom-bem1 bc-14E + px (K338M, K348A, R349A & R369A)-Pk3::HIS3, CDC28prom-cdc28 Y19F-CDC28term::kanMX6, URA3::CLN2-3xHA*
3338	*MATa, his3Δ1, leu2Δ0, met15Δ0, ura3Δ0, cdc24 K513A, leu2::LEU2::TIR1, bem1-AID-6xFLAG::hphNT1, his3::BEM1prom-bem1 bc-14E + px (K338M, K348A, R349A & R369A)-Pk3::HIS3, CDC28prom-cdc28 Y19F-CDC28term::kanMX6, SPC42-yomKate2::CaURA3, SEC3-GFP::natNT2*
3350	*MATa, his3Δ1, leu2Δ0, met15Δ0, ura3Δ0, CLB5-sfGFP::kanMX6, CLB2-mScarletI3-mScarletI3::HIS3*
3352	*MATa, his3Δ1, leu2Δ0, met15Δ0, ura3Δ0, cdc24 K513A-3xHA::URA3, bem1 bc-14E + px (K338M, K348A, R349A & R369A), CLB5-sfGFP::kanMX6, CLB2-mScarletI3-mScarletI3::HIS3*
3370	*MATa, his3Δ1, leu2Δ0, met15Δ0, ura3Δ0, cdc24 K513A, leu2::LEU2::TIR1, bem1-AID-6xFLAG::hphNT1, his3::BEM1prom-bem1 bc-14E + px (K338M, K348A, R349A & R369A)-Pk3::HIS3, URA3::CLN2-3xHA, cln1Δ::kanMX6, SPC42-GFP::natNT2*
3372	*MATa, his3Δ1, leu2Δ0, met15Δ0, ura3Δ0, cdc24 K513A, leu2::LEU2::TIR1, bem1-AID-6xFLAG::hphNT1, his3::BEM1prom-bem1 bc-14E + px (K338M, K348A, R349A & R369A)-Pk3::HIS3, URA3::cln2-24A-3xHA (S233A, S236A, S239A, T242A, T378A, S380A, T381A, S383A, S385A, S389A, S391A, T392A, S393A, S396A, S399A, S400A, S401A, S403A, T468A, S472A, T476A, S528A, S529A, S530A), cln1Δ::kanMX6, SPC42-GFP::natNT2*
3374	*MATa, his3Δ1, leu2Δ0, met15Δ0, ura3Δ0, cdc24 K513A, leu2::LEU2::TIR1, bem1-AID-6xFLAG::hphNT1, his3::BEM1prom-bem1 bc-14E + px (K338M, K348A, R349A & R369A)-Pk3::HIS3, URA3::cln2-24D-3xHA (S233D, S236D, S239D, T242D, T378D, S380D, T381D, S383D, S385D, S389D, S391D, T392D, S393D, S396D, S399D, S400D, S401D, S403D, T468D, S472D, T476D, S528D, S529D, S530D), cln1Δ::kanMX6, SPC42-GFP::natNT2*
3382	*MATa, his3Δ1, leu2Δ0, ura3Δ0, bnr1Δ::kanMX6, bni1-FH2::HIS3, URA3::CLN2-3xHA*
3392	*MATa, his3Δ1, leu2Δ0, met15Δ0, ura3Δ0, URA3::kanMX6::GAL1prom-CLN2-3xHA*
3394	*MATa, his3Δ1, leu2Δ0, met15Δ0, ura3Δ0, URA3::kanMX6::GAL1prom-cln2-24D-3xHA (S233D, S236D, S239D, T242D, T378D, S380D, T381D, S383D, S385D, S389D, S391D, T392D, S393D, S396D, S399D, S400D, S401D, S403D, T468D, S472D, T476D, S528D, S529D, S530D)*
3396	*MATa, his3Δ1, leu2Δ0, met15Δ0, ura3Δ0, URA3::kanMX6::GAL1prom-cln2-24A-3xHA (S233A, S236A, S239A, T242A, T378A, S380A, T381A, S383A, S385A, S389A, S391A, T392A, S393A, S396A, S399A, S400A, S401A, S403A, T468A, S472A, T476A, S528A, S529A, S530A)*
3443	*MATa, his3Δ1, leu2Δ0, met15Δ0, ura3Δ0, cdc24 K513A, leu2::LEU2::TIR1, bem1-AID-6xFLAG::hphNT1, his::BEM1-Pk3::HIS3, CLN2-sfGFP-CLN2term::kanMX6, PUS1-mCherry::natNT2*
3445	*MATa, his3Δ1, leu2Δ0, met15Δ0, ura3Δ0, cdc24 K513A, leu2::LEU2::TIR1, bem1-AID-6xFLAG::hphNT1, his3::BEM1prom-bem1 bc-14E + px (K338M, K348A, R349A & R369A)-Pk3::HIS3, CLN2-sfGFP-CLN2term::kanMX6, PUS1-mCherry::natNT2*
3461	*MATa, his3Δ11, leu2Δ3,112, trp1Δ1, ura3Δ52, ade2Δ1, can1Δ100, GAL+, bar1, sec6-4::HIS3, CLN2-3xHA::LEU2, CLN2-sfGFP-CLN2term::kanMX6, PUS1-mCherry::natNT2*

3474	*MATa, his3Δ1, leu2Δ0, met15Δ0, ura3Δ0, cdc24 K513A, leu2::LEU2::TIR1, bem1-AID-6xFLAG::hphNT1, his::BEM1-Pk3::HIS3, natNT2::MET25prom-3xHA-CLN2*
3477	*MATa, his3Δ1, leu2Δ0, met15Δ0, ura3Δ0, cdc24 K513A, leu2::LEU2::TIR1, bem1-AID-6xFLAG::hphNT1, his3::BEM1prom-bem1 bc-14E + px (K338M, K348A, R349A & R369A)-Pk3::HIS3, natNT2::MET25prom-3xHA-CLN2*
3482	*MATa, his3Δ1, leu2Δ0, met15Δ0, ura3Δ0, cdc24 K513A, leu2::LEU2::TIR1, bem1-AID-6xFLAG::hphNT1, his3::BEM1prom-bem1 bc-14E + px (K338M, K348A, R349A & R369A)-Pk3::HIS3, URA3::cln2-24A (S233A, S236A, S239A, T242A, T378A, S380A, T381A, S383A, S385A, S389A, S391A, T392A, S393A, S396A, S399A, S400A, S401A, S403A, T468A, S472A, T476A, S528A, S529A, S530A)-sfGFP-CLN2term::kanMX6, PUS1-mCherry::natNT2*
3484	*MATa, his3Δ1, leu2Δ0, met15Δ0, ura3Δ0, cdc24 K513A, leu2::LEU2::TIR1, bem1-AID-6xFLAG::hphNT1, his3::BEM1prom-bem1 bc-14E + px (K338M, K348A, R349A & R369A)-Pk3::HIS3, URA3::cln2-24D (S233D, S236D, S239D, T242D, T378D, S380D, T381D, S383D, S385D, S389D, S391D, T392D, S393D, S396D, S399D, S400D, S401D, S403D, T468D, S472D, T476D, S528D, S529D, S530D)-sfGFP-CLN2term::kanMX6, PUS1-mCherry::natNT2*
3525	*MATa, his3Δ1, leu2Δ0, met15Δ0, ura3Δ0, cdc24 K513A, leu2::LEU2::TIR1, bem1-AID-6xFLAG::hphNT1, his3::BEM1prom-bem1 bc-14E + px (K338M, K348A, R349A & R369A)-Pk3::HIS3, swe1Δ::kanMX6, URA3::CLN2-3xHA*
3539	*MATa, his3Δ1, leu2Δ0, met15Δ0, ura3Δ0, cdc24 K513A, leu2::LEU2::TIR1, bem1-AID-6xFLAG::hphNT1, his::BEM1-Pk3::HIS3, PUS1-mScarletI3::CaURA3, natNT2::MET25prom-CLN2-3xHA-sfGFP::kanMX6*
3541	*MATa, his3Δ1, leu2Δ0, met15Δ0, ura3Δ0, cdc24 K513A, leu2::LEU2::TIR1, bem1-AID-6xFLAG::hphNT1, his3::BEM1prom-bem1 bc-14E + px (K338M, K348A, R349A & R369A)-Pk3::HIS3, PUS1-mScarletI3::CaURA3, natNT2::MET25prom-CLN2-3xHA-sfGFP::kanMX6*
3543	*MATa, his3Δ1, leu2Δ0, met15Δ0, ura3Δ0, cdc24 K513A, leu2::LEU2::TIR1, bem1-AID-6xFLAG::hphNT1, his3::BEM1prom-bem1 bc-14E + px (K338M, K348A, R349A & R369A)-Pk3::HIS3, PUS1-mScarletI3::CaURA3, natNT2::MET25prom-cln2-4T3S (T311A, T381A, S396A, T405A, S427A, T430A, S518A)-3xHA-sfGFP::kanMX6*
3554	*MATa, his3Δ1, leu2Δ0, met15Δ0, ura3Δ0, cdc24 K513A, leu2::LEU2::TIR1, bem1-AID-6xFLAG::hphNT1, his::BEM1-Pk3::HIS3, PUS1-mScarletI3::CaURA3, natNT2::MET25prom-cln2-4T3S (T311A, T381A, S396A, T405A, S427A, T430A, S518A)-3xHA-sfGFP::kanMX6*
3560	*MATa, his3Δ1, leu2Δ0, met15Δ0, ura3Δ0, cdc24 K513A, leu2::LEU2::TIR1, bem1-AID-6xFLAG::hphNT1, his3::BEM1prom-bem1 bc-14E + px (K338M, K348A, R349A & R369A)-Pk3::HIS3, PUS1-mCherry::natNT2, URA3::cln2-24A (S233A, S236A, S239A, T242A, T378A, S380A, T381A, S383A, S385A, S389A, S391A, T392A, S393A, S396A, S399A, S400A, S401A, S403A, T468A, S472A, T476A, S528A, S529A, S530A)-sfGFP-NES::kanMX6*
3563	*MATa, his3Δ1, leu2Δ0, met15Δ0, ura3Δ0, cdc24 K513A, leu2::LEU2::TIR1, bem1-AID-6xFLAG::hphNT1, his3::BEM1prom-bem1 bc-14E + px (K338M, K348A, R349A & R369A)-Pk3::HIS3, PUS1-mScarletI3::CaURA3, natNT2::MET25prom-cln2-22A (S233A, S236A, S239A, T242A, T378A, S380A, S383A, S385A, S389A, S391A, T392A, S393A, S399A, S400A, S401A, S403A, T468A, S472A, T476A, S528A, S529A, S530A)-3xHA-sfGFP::kanMX6*

References

1. Funakoshi, M. & Hochstrasser, M. Small epitope-linker modules for PCR-based C-terminal tagging in Saccharomyces cerevisiae. *Yeast.* **26**, 185–192 (2009).
2. Sikorski, R. S. & Hieter, P. A system of shuttle vectors and yeast host strains designed for efficient manipulation of DNA in Saccharomyces cerevisiae. *Genetics* **122**, 19–27 (1989).
3. Morawska, M. & Ulrich, H. D. An expanded tool kit for the auxin-inducible degron system in budding yeast: A tool kit for the AID system. *Yeast* **30**, 341–351 (2013).
4. Eng, T., Guacci, V. & Koshland, D. ROCC, a conserved region in cohesin's Mcd1 subunit, is essential for the proper regulation of the maintenance of cohesion and establishment of condensation. *Mol. Biol. Cell* **25**, 2351–2364 (2014).
5. Longtine, M. S. *et al.* Additional modules for versatile and economical PCR-based gene deletion and modification in Saccharomyces cerevisiae. *Yeast* **14**, 953–961 (1998).
6. Markus, S. M., Omer, S., Baranowski, K. & Lee, W.-L. Improved Plasmids for Fluorescent Protein Tagging of Microtubules in Saccharomyces cerevisiae. *Traffic.* **16**, 773–786 (2015).
7. Pirincci Ercan, D. *et al.* Budding yeast relies on G1 cyclin specificity to couple cell cycle progression with morphogenetic development. *Sci. Adv.* **7**, eabg0007 (2021).
8. Garmendia-Torres, C., Tassy, O., Matifas, A., Molina, N. & Charvin, G. Multiple inputs ensure yeast cell size homeostasis during cell cycle progression. *eLife* **7**, e34025 (2018).
9. Lee, S., Lim, W. A. & Thorn, K. S. Improved blue, green, and red fluorescent protein tagging vectors for S. cerevisiae. *PloS One* **8**, e67902 (2013).
10. Tyers, M., Tokiwa, G. & Futcher, B. Comparison of the Saccharomyces cerevisiae G1 cyclins: Cln3 may be an upstream activator of Cln1, Cln2 and other cyclins. *EMBO J.* **12**, 1955–1968 (1993).

11. Malcova, I., Farkasovsky, M., Senohrabkova, L., Vasicova, P. & Hasek, J. New integrative modules for multicolor-protein labeling and live-cell imaging in Saccharomyces cerevisiae. *FEMS Yeast Res.* **16**, fow027 (2016).

12. Hodel, A. E. *et al.* Nuclear Localization Signal Receptor Affinity Correlates with in Vivo Localization in Saccharomyces cerevisiae. *J. Biol. Chem.* **281**, 23545–23556 (2006).

13. Janke, C. *et al.* A versatile toolbox for PCR-based tagging of yeast genes: new fluorescent proteins, more markers and promoter substitution cassettes. *Yeast* **21**, 947–962 (2004).
