# Supplementary material for: A cyclin-polarity feedback network ensures healthy cell proliferation
Source: Nat Commun. 2026 Jun 19;17:7731. doi: 10.1038/s41467-026-74596-7 (PMC13434654; doi:10.1038/s41467-026-74596-7)
Supplement: Supplementary file 4 [file 41467_2026_74596_MOESM2_ESM.pdf]

Description of Additional Supplementary Files

Supplementary Movie 1. Sec3-GFP (exocyst) dynamics in a WT control cell.

Time-lapse images of WT cells expressing Sec3-GFP (green). Images are maximum-intensity projected z-stacks. Time, hours:minutes. Scale bar, 5 µm. The white arrow shows Sec3 polarization that delineates a stable polarity axis. This arrow turns red after bud emergence. The video corresponds to Fig. 1a (upper panel).

Supplementary Movie 2. Sec3-GFP (exocyst) dynamics in a *bem1^{lip} cdc24^{lip}* mutant cell displaying polarity establishment and maintenance defects.

Time-lapse images of *bem1^{lip} cdc24^{lip}* mutant cells expressing Sec3-GFP (green). Images are maximum-intensity projected z-stacks. Time, hours:minutes. Scale bar, 5 µm. The white arrows points to two unsuccessful Sec3 polarization attempts, before a final successful attempt that results in bud emergence (white arrow turns red). The video corresponds to Fig. 1a (lower panel).

Supplementary Movie 3. Whi5-sfGFP dynamics in WT and *bem1^{lip} cdc24^{lip}* mutant cells.

Time-lapse images of WT (**A**) and *bem1^{lip} cdc24^{lip}* mutant cells (**B**) expressing Whi5-sfGFP (green) and Pus1-mRuby2 (nucleoplasm, magenta). Images are maximum-intensity projected z-stacks. Time, hours:minutes. Scale bar, 5 µm. Time 0 represents anaphase onset in the previous cell cycle. Colocalization of Whi5 and Pus1 is shown in white. The video corresponds to Supplementary Fig. 1d.

Supplementary Movie 4. S- and M- phase cyclin expression is prolonged in *bem1^{lip} cdc24^{lip}* mutant cells.

Time-lapse images of WT (**A**) and *bem1^{lip} cdc24^{lip}* mutant cells with mild (**B**) and severe (**C**) polarity defects expressing Clb5-sfGFP (S-phase cyclin, cyan) and Clb2-2xmScarletI3 (M-phase cyclin, magenta). Images are maximum-intensity projected z-stacks. Time, hours:minutes. Scale bar, 5 µm. Colocalization of Clb5 and Clb2 is shown in white. The expressions of S- and M-phase cyclins are confined to brief temporal windows in WT cells (panel A, upper panel in Fig.

1h). In panel B, the cell on the left corresponds to Fig. 1h (middle panel), whereas the cell on the right shows an unbudded cell that displayed prolonged Clb2 expression. Unbudded *bem1^{lip}* *cdc24^{lip}* mutant cells exhibit a mitotic delay and become multinucleate if a daughter cell is not generated (panel C, lower panel in Fig. 1h).

Supplementary Movie 5. Strong polarity defects in *bem1^{lip} cdc24^{lip}* mutant cells impact the ordering of Myo1 polarization and SPB separation events.

Time-lapse images of WT (**A**) and *bem1^{lip} cdc24^{lip}* mutant cells (**B**) expressing Myo1-GFP (white), Spc42-GFP (SPBs, white) and Pus1-mRuby2 (nucleoplasm, red). Images are maximum-intensity projected z-stacks. Time, hours:minutes. Scale bar, 5 µm. Time 0 represents anaphase onset in the previous cell cycle. The cyan arrow shows Myo1 localization whereas the magenta arrow highlights SPB separation. The video corresponds to Supplementary Fig. 3a. In panel B, the cell on the right corresponds to the mother cell presented in Supplementary Fig. 3a (lower panel), in which polarity establishment is severely delayed, whereas the cell on the left corresponds to the daughter cell, in which polarity establishment is mildly delayed and correct cell cycle ordering is maintained.

Supplementary Movie 6. SPB separation and Clb2 expression can be observed before bud emergence in *bem1^{lip} cdc24^{lip}* mutant cells.

Time-lapse images of WT (**A**) and *bem1^{lip} cdc24^{lip}* mutant cells with mild (**B**) and severe (**C**) polarity defects expressing Clb2-sfGFP (M-phase cyclin, magenta) and Spc42-yomKate2 (SPBs, white). Images are maximum-intensity projected z-stacks. Time, hours:minutes. Scale bar, 5 µm. In WT, Clb2 expression occurs only after bud emergence and SPB separation (panel A, upper panel in Supplementary Fig. 3d). The cell in the middle of panel B corresponds to Supplementary Fig. 3d (middle panel) and displays polarity defects that result in SPB separation and Clb2 expression occurring before bud emergence. The cell in the right in panel C corresponds to Supplementary Fig. 3d (lower panel) and displays polarity defects that result in appearance of a multinucleate cell.

Supplementary Movie 7. Misordering of cell cycle events and the appearance of multinucleate cells in the *bem1*^{lip} *cdc24*^{lip} mutant.

Time-lapse images of a WT (**A**) and a *bem1*^{lip} *cdc24*^{lip} mutant cells (**B**) expressing Venus-Tub1 (microtubules, white), Spc42-GFP (SPBs, white) and Pus1-mRuby2 (nucleoplasm, red). Images are maximum-intensity projected z-stacks. Time, hours:minutes. Scale bar, 5 µm. In contrast to WT cells, where ordering of cell cycle events is invariant, mitotic spindle formation and nuclear division can occur before bud emergence in *bem1*^{lip} *cdc24*^{lip} mutant cells. As a consequence, these types of cells become multinucleate. The video corresponds to Supplementary Fig. 3e.

Supplementary Movie 8. Sec3 and SPBs dynamics in the AID system.

Time-lapse images of WT cells (**A**), *bem1*^{lip} *cdc24*^{lip} mutant cells with no (**B**) mild (**C**) and severe (**D**) polarity defects and *bem1*^{lip} *cdc24*^{lip} *cdc28*^{cdk1} *Y19F* mutant cells (**E**) in the AID system expressing Sec3-GFP (exocyst, green) and Spc42-yomKate2 (SPBs, white). Images are maximum-intensity projected z-stacks. Time, hours:minutes. Scale bar, 5 µm. The cells were synchronized in G1 phase, bem1-AID was degraded and cells were released into the cell cycle. The cell in the middle of panel A corresponds to Fig. 2b (first panel). As observed in WT cells, Sec3 in panel B remains at the same site on the plasma membrane until bud emergence (second panel in Fig. 2b). The cell in panel C (third panel in Fig. 2b) shows one unsuccessful Sec3 polarization attempt that results in delay of bud emergence. The time between SPB separation and anaphase onset is increased in comparison to *bem1*^{lip} *cdc24*^{lip} mutant cells that present a stable pole. The cell in panel D (fourth panel in Fig. 2b) shows two unsuccessful Sec3 polarization attempts and does not bud. The time between SPB separation and anaphase onset is increased in comparison to *bem1*^{lip} *cdc24*^{lip} mutant cells that are budding. In panel E (fifth panel in Fig. 2b), the cell shows unstable Sec3 polarization and become multinucleate. The time between SPB separation and anaphase onset is similar to WT timing.

Supplementary Movie 9. The time from SPB separation to anaphase onset is reduced in *bem1*^{lip} *cdc24*^{lip} *cln2-24A*, while being delayed in *bem1*^{lip} *cdc24*^{lip} *cln2-24D* mutant cells (AID system).

Time-lapse images of *bem1*^{lip} *cdc24*^{lip} (**A**), *bem1*^{lip} *cdc24*^{lip} *cln2-24A* (**B**) and *bem1*^{lip} *cdc24*^{lip} *cln2-24D* (**C**) mutant cells in the AID system expressing Spc42-GFP (SPBs, white). Images are maximum-intensity projected z-stacks. Time, hours:minutes. Scale bar, 5 µm. The

cells were synchronized in G1 phase, bem1-AID was degraded and cells were released into the cell cycle. The video corresponds to Fig. 4g.

Supplementary Movie 10. Cln2 dynamics and localization in the AID system.

Time-lapse images of WT cells (**A**) and *bem1^{lip} cdc24^{lip}* mutant cells with mild (**B**) and severe (**C**) polarity defects in the AID system expressing Cln2-sfGFP (G1-phase cyclin, green) and Pus1-mCherry (nucleoplasm, magenta). Images are maximum-intensity projected z-stacks. Time, hours:minutes. Scale bar, 5 µm. Colocalization of Cln2 and Pus1 is shown in white. The cells were synchronized in G1 phase, bem1-AID was degraded and cells were released into the cell cycle. Panel A corresponds to Fig. 5a (upper panel) and shows that Cln2 prominently localizes to the nucleus in WT cells. The cell in panel B displays prominent cytoplasmic localization of Cln2 which is rapidly degraded after bud emergence (middle panel in Fig. 5a), whereas the unbudded cell from panel C (lower panel in Fig. 5a) displays enrichment and stabilization of cytoplasmic Cln2.

Supplementary Movie 11. Cln2 prominently localizes to the nucleus in unbudded *bem1^{lip} cdc24^{lip} cln2-24A* cells whereas it is enriched in the cytoplasm in *bem1^{lip} cdc24^{lip} cln2-24D* cells (AID system).

Time-lapse images of *bem1^{lip} cdc24^{lip} cln2-24A* mutant cells (**A**) and *bem1^{lip} cdc24^{lip} cln2-24D* mutant cells (**B**) in the AID system. The green signal corresponds to cln2-24A-sfGFP (**A**) or cln2-24D-sfGFP (**B**) and the magenta signal corresponds to Pus1-mCherry (nucleoplasm, magenta). Images are maximum-intensity projected z-stacks. Time, hours:minutes. Scale bar, 5 µm. Colocalization of cln2 and Pus1 is shown in white. The cells were synchronized in G1 phase, bem1-AID was degraded and cells were released into the cell cycle. The video corresponds to Fig. 5c (first and second panel respectively).

Supplementary Movie 12. The addition of NES sequence increases the percentage of budded cells in the *bem1^{lip} cdc24^{lip} cln2-24A* mutant (AID system).

Time-lapse images of *bem1^{lip} cdc24^{lip} cln2-24A-NES* (Nuclear Export Signal) mutant cells in the AID system. The green signal corresponds to cln2-24A-sfGFP-NES (G1-phase cyclin) and the magenta signal corresponds to Pus1-mCherry (nucleoplasm, magenta). Images are maximum-intensity projected z-stacks. Time, hours:minutes. Scale bar, 5 µm. The cells were synchronized

in G1 phase, bem1-AID was degraded and cells were released into the cell cycle. The video corresponds to Fig. 5c (third and last panel respectively).

Supplementary Movie 13. Cln2 prominently localizes to the nucleus in unbudded *$bem1^{lip}$ $cdc24^{lip}$ cln2-22A* mutant cells (AID system).

Time-lapse images WT cells (**A**), *$bem1^{lip}$ $cdc24^{lip}$* cells (**B**) and *$bem1^{lip}$ $cdc24^{lip}$ cln2-22A* cells (**C**) in the AID system. The magenta signal corresponds to Pus1-mCherry (nucleoplasm, magenta) and the green signal corresponds to Cln2-3xHA-sfGFP (**A**, **B**) or cln2-22A-3xHA-sfGFP (**C**) under the control of the *MET25* promoter. Images are maximum-intensity projected z-stacks. Time, hours:minutes. Scale bar, 5 µm. Cells were arrested in G1 and treated with auxin for the final 30 minutes of alpha-factor arrest. These cells were then released into the cell cycle and grown for 70 minutes in a methionine depleted medium to express the G1 cyclin. Finally, cells were mounted on a gel containing methionine then imaged. Colocalization of Cln2 and Pus1 is shown in white. The video corresponds to Supplementary Figs. 12a.

Supplementary Movie 14. Unbudded *$bem1^{lip}$ $cdc24^{lip}$ $cln2^{4T3S}$* mutant cells display enrichment of cytoplasmic $cln2^{4T3S}$ (AID system).

Time-lapse images of *BEM1 $cln2^{4T3S}$* cells (**A**) and *$bem1^{lip}$ $cdc24^{lip}$ $cln2^{4T3S}$* cells (**B**) in the AID system expressing $cln2^{4T3S}$-3xHA-sfGFP (G1-phase cyclin, green) under the control of the *MET25* promoter and Pus1-mScarletI3 (nucleoplasm, magenta). Images are maximum-intensity projected z-stacks. Time, hours:minutes. Scale bar, 5 µm. The cells were arrested in G1 and treated with auxin for the final 30 minutes of alpha-factor arrest. These cells were then released into the cell cycle and grown for 70 minutes in a methionine depleted medium to express $cln2^{4T3S}$. Finally, cells were mounted on a gel containing methionine then imaged. Colocalization of Cln2 and Pus1 is shown in white. The video corresponds to Supplementary Figs. 12b.
